# COVID-19 hospitalizations forecasts using internet search data

**DOI:** 10.1038/s41598-022-13162-9

**Published:** 2022-06-11

**Authors:** Tao Wang, Simin Ma, Soobin Baek, Shihao Yang

**Affiliations:** grid.213917.f 0000 0001 2097 4943 H. Milton Stewart School of Industrial and Systems Engineering, Georgia Institute of Technology, Atlanta, GA 30309 USA

**Keywords:** Statistics, Epidemiology, Computational models

## Abstract

As the COVID-19 spread over the globe and new variants of COVID-19 keep occurring, reliable real-time forecasts of COVID-19 hospitalizations are critical for public health decisions on medical resources allocations. This paper aims to forecast future 2 weeks national and state-level COVID-19 new hospital admissions in the United States. Our method is inspired by the strong association between public search behavior and hospitalization admissions and is extended from a previously-proposed influenza tracking model, AutoRegression with GOogle search data (ARGO). Our LASSO-penalized linear regression method efficiently combines Google search information and COVID-19 related time series information with dynamic training and rolling window prediction. Compared to other publicly available models collected from COVID-19 forecast hub, our method achieves substantial error reduction in a retrospective out-of-sample evaluation from Jan 4, 2021, to Dec 27, 2021. Overall, we showed that our method is flexible, self-correcting, robust, accurate, and interpretable, making it a potentially powerful tool to assist healthcare officials and decision making for the current and future infectious disease outbreaks.

## Introduction

COVID-19 (SARS-CoV-19), an acute respiratory syndrome disease caused by a coronavirus, has spread worldwide causing over 120,695,785 reported cases and 4,987,755 reported deaths^1^. During the continuous spread of COVID-19, many variants (alpha, delta, omicron, etc.) of COVID-19 emerge, leading to drastic surges in hospital admissions and shortages in health care resources^2^. Therefore, an accurate hospital admissions forecasting model is crucial to assist hospitals and policymakers with the possibilities and the timings of rapid changes, so as to further respond to and prepare for future COVID-19 spread.

The Centers for Disease Control and Prevention (CDC)^3^ has been collecting hospitalizations predictions from various research teams and making ensemble and baseline predictions since May 2020. According to the weekly COVID-19 forecasts submissions compiled by CDC^3^, machine learning^4,5^ and compartmental models^6,7^ are the most popular forecasting approaches^4,5^. For example, Rodríguez et al.^4^ use a neural network architecture incorporating COVID-19 time series, and mobility information as inputs, whereas Jin et al.^5^ utilize attention and transformer models to compare and combine past COVID-19 trends for future forecasts. On the other hand, Vespignani et al.^6^ and Kinsey el al.^7^ adapt SEIR (compartmental model) as the baseline structure and combine different exogenous variables including spatial-temporal and mobility information to build more sophisticated models to capture COVID-19 disease dynamics and forecast hospitalizations. Meanwhile, statistical and data-driven models, taking advantage of COVID-19 public search information for hospitalizations predictions, have not drawn much attention.

For the last decade, numerous studies have suggested online search data could be a valuable component to monitor and forecast infectious diseases, such as influenza^8–13^, HIV/AIDS^14^, dengue^15^, etc. For instance, Google Flu Trend (GFT)^8^, a digital disease detection system that uses the volume of selected Google search terms to estimate current influenza-like illnesses (ILI) activity, was the first among many studies to demonstrate how big data and public search behavior compliment traditional statistical predictive analysis. Later, many studies proposed different methodologies to improve upon GFT and provide more robust and accurate real-time forecast estimates of influenza activity in the US, including machine learning models^10^, statistical models^9^, ensemble models^11^. Online search information from other sources, such as Yahoo^12^ and Baidu^13^, have demonstrated their predictive power for influenza tracking as well.

On the other hand, to best of our knowledge, the only hospitalizations prediction model that is based on the Internet search data is a vector error correction model (VECM) proposed by Turk et al.^16^. VECM combines Google search data and healthcare chatbot scores and produces COVID-19 hospitalizations predictions in Greater Charlotte market area, where the Google search queries are selected based on existing literature, and filtered through hand-craft procedures. Yet, their selection approach highly depends on literature considered and healthcare chatbot scores can be limited in-access across different geographical areas. So far, none of the existing Internet-search-based methods provide robust weeks-ahead hospitalisation forecasts for different geographical areas in the United States that fully utilize COVID-19 related online search queries and account for their sparsity and noisiness.

In this paper, we propose the ARGO inspired model (ARGO), which combines online search data and lagged COVID-19 related information in a L1-norm penalized linear regression model to produce real-time 1–2 weeks ahead national and state-level hospitalizations predictions over 12-month out-of-sample evaluation period in the United States. Our results show that ARGO, leveraging predictive information in the selected interpretable Google search queries, is able to largely outperform baseline models and all other public available models from CDC’s COVID-19 forecast hub, which implies potential in ARGO to help with urgent public health decisions on health resources allocations.

### Our contribution

We propose a LASSO-penalized linear regression model, efficiently combining Google search information and COVID-19 related time series information, for United States national and state-level 1 and 2-week-ahead COVID-19 hospitalization forecasts, with dynamic training and rolling window prediction. We identified 11 important query terms from the initial large pool of relevant Google search queries from previous studies, and calculated the optimal lag between search data and COVID-19 hospitalization. The important terms consist of symptom-related terms and general COVID-19 related terms, possessing strong representation of upcoming COVID-19 surges, which enhances the robustness and the interpretability of our forecasts. Finally the selected Google search data, vaccination rate, past COVID-19 cases and hospitalizations are all combined to produce both national and state-level forecasts. Our selection of search queries is systematic and comprehensive, and our method is intentionally straightforward and unified to prevent overfitting. Numerical comparisons show that our method performs competitively with other publicly available COVID-19 forecasts. This study further emphasizes the general applicability and the predictive power of online search data for various tasks in disease surveillance.

## Results

**Figure 1:**
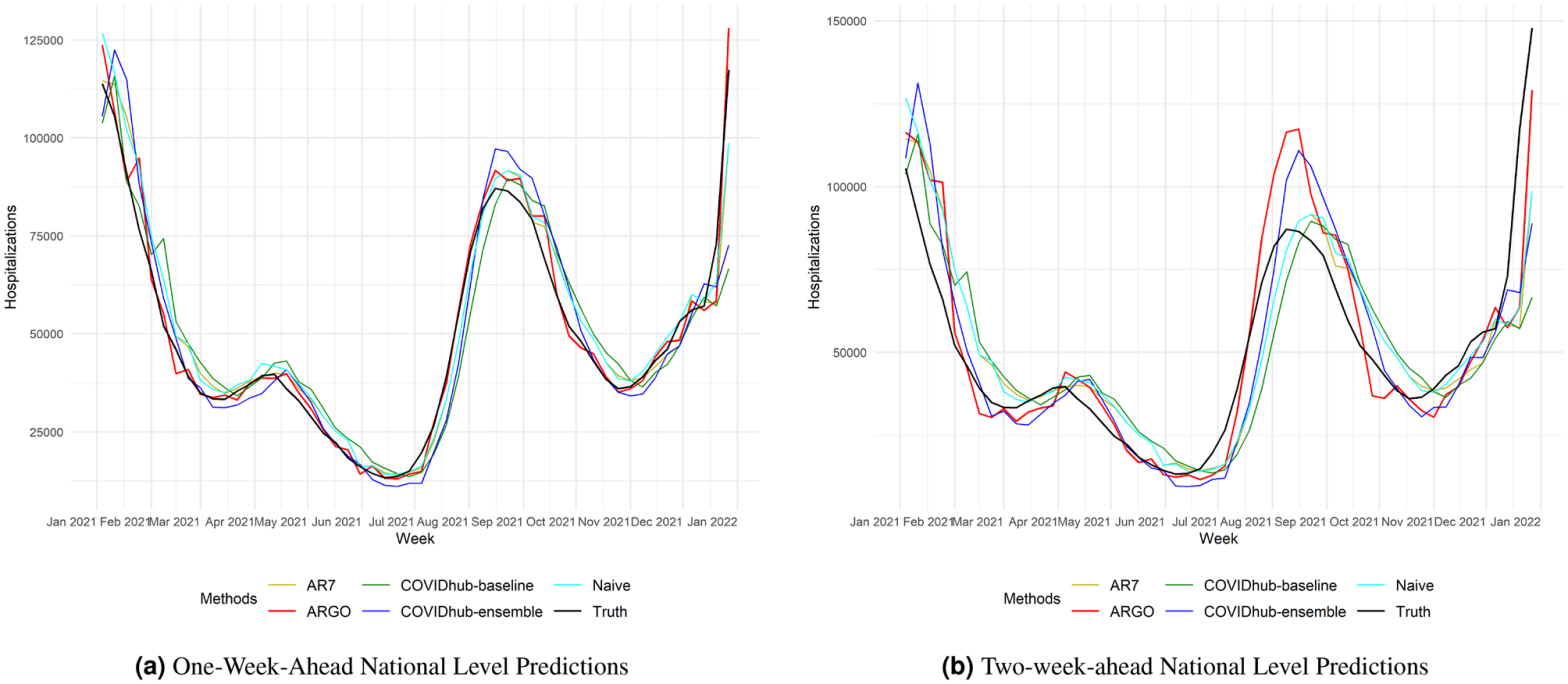
One-week-ahead and two-week-ahead hospitalizations predictions of the 5 compared models. 1 and 2 weeks ahead predictions are compared weekly from 2021-01-04 to 2021-12-27. True new hospital admissions released by HHS are marked in black. Predictions of the 5 models, which consist of ARGO, AR7, naive, COVIDhub-baseline, COVIDhub-ensemble, are marked in red, gold, cyan, green, and blue, respectively.

**Figure 2:**
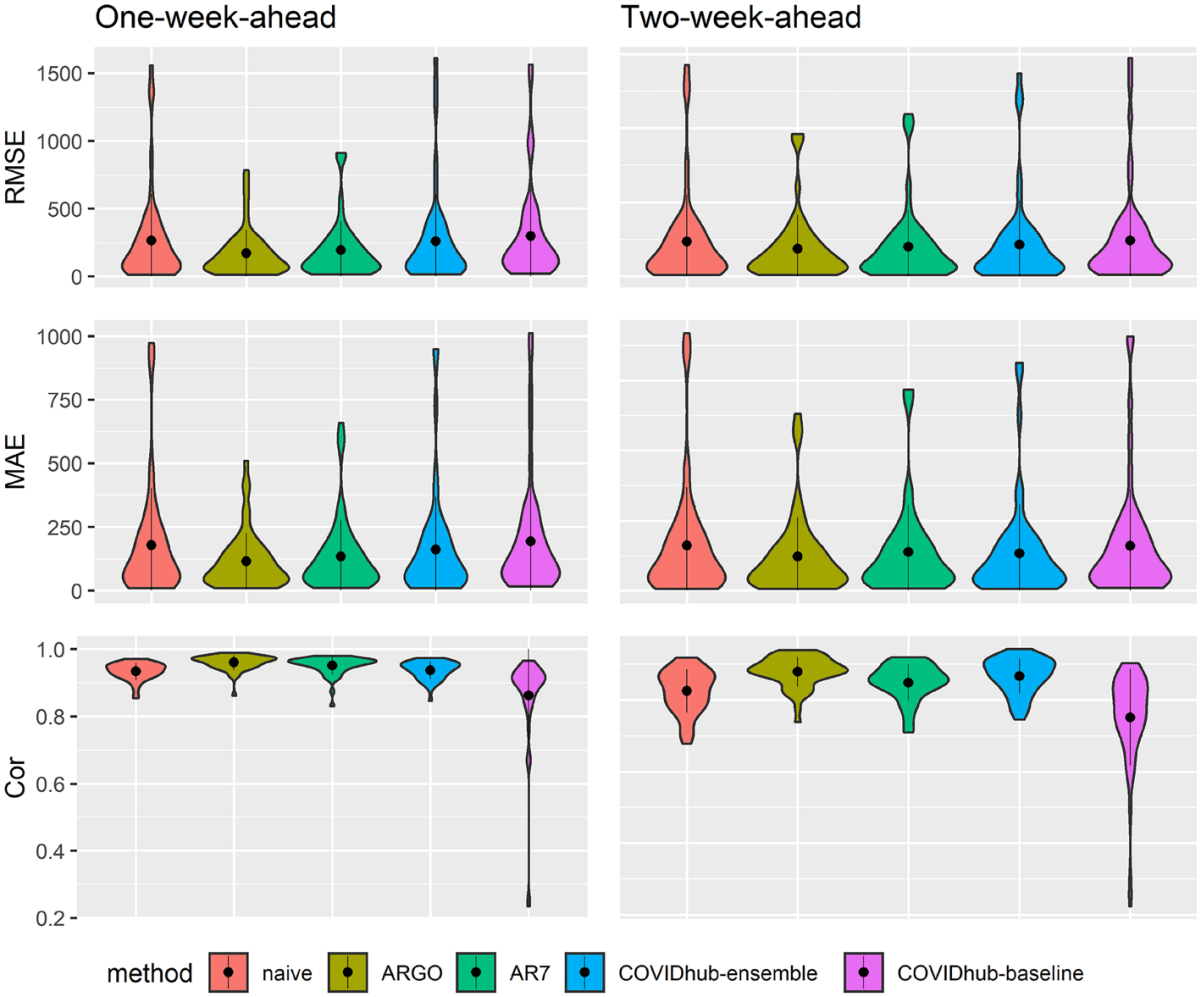
The distributions of error metrics of one-week-ahead and two-week-ahead predictions from 2021-01-04 to 2021-12-27 over the 51 states of the 5 compared models. The embedded black dot indicates mean. The vertical line represents 1 standard deviation range which is trimmed to be inside normal range of RMSE, MAE and correlations coefficients.

We conducted retrospective evaluation of weekly hospital admissions for the period between January 4, 2021 and December 27, 2021, on both national and state level. To evaluate prediction performance, we calculated the root mean square error (RMSE), the mean absolute error (MAE) and the Pearson correlation coefficient (Cor) of one-week-ahead and two-week-ahead predictions (detailed in the Methods section). All comparisons are based on the original scale of the ground truth of new hospital admissions released by US Department of Health and Human Services (HHS)^17^ (Figs. 1, 2).

### Descriptive analysis of the input data

**Figure 3:**
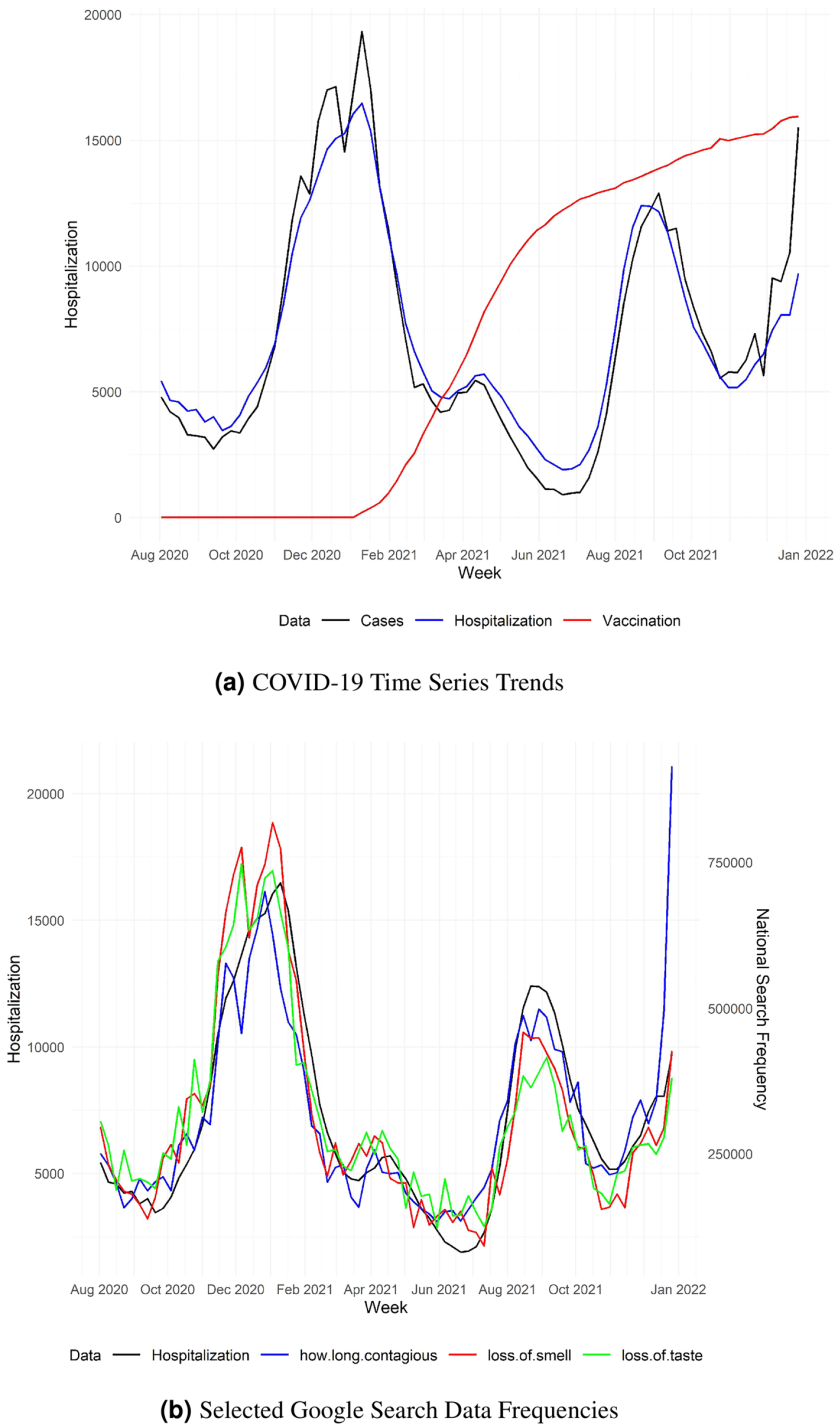
Left: National weekly COVID-19 new hospitalizations (black), national weekly COVID-19 confirmed Cases (blue) and national percentage of vaccinated population (red), scaled accordingly. Right: COVID-19 weekly new hospitalizations (red) and the top 3 Google queries (optimal lagged) that have largest correlation coefficients with hospitalizationsm: “how long contagious” (blue), “loss of smell” (red) and “loss of taste” (green).

Figure 3a visualizes the three COVID-19 related data through time series plots. It can be observed that national COVID-19 confirmed cases have similar up-and-down trend to national COVID-19 hospitalizations with no obvious lagging, confirming the intuition that cases lead to hospitalizations. On the other hand, the percentages of vaccinated population increase fast between February 2021 and June 2021 but slow down around July 2021, exhibiting a strong negative correlation with hospitalization and indicating vaccination potentially leads to drop in hospitalization.

Figure 3b displays the top 3 most correlated Google search queries’ frequencies and hospitalization, further illustrating the strong prediction power of related public search information on future hospitalization trends. Such prediction power persists throughout the entire period as the pandemic progresses. We also note that seemingly similar query terms can have slightly different search frequencies, due to subtle differences in search preference. For example, “loss of smell” and “loss of taste” tend to happen together, but around mid-November 2020, a spike occurs on the curve of query “loss of taste” while a similar spike occurs a few days later on the curve of query “loss of smell”. Even though the symptoms can occur together, the search pattern could still be different due to disparities in linguistic and information-seeking behavior, or the inherent noise of Google search data.

### Comparison methods

To further demonstrate the prediction accuracy and robustness of ARGO, we collected hospitalizations predictions of the two benchmark methods (COVIDhub-baseline and COVIDhub-ensemble) from COVID-19 forecast hub^3^, the official CDC COVID-19 Forecasting page. COVID-19 Forecast hub collects predictions from all contributing teams that submit hospitalization forecasts since January 15th, 2020. The COVIDhub-baseline is a persistent method that uses latest daily observation as future daily predictions^3^. The COVIDhub-ensemble uses medians of hospitalizations predictions submitted to the COVID-19 forecast hub as its “ensemble” forecasts. We also consider the top 5 CDC published teams for prediction comparison, among over 100 teams submitted to CDC after filtering out missing values in reporting periods and/or states. Due to the space limit, we only include COVIDhub-baseline and COVIDhub-ensemble in the main text, leaving the full comparison of the top individual teams from CDC’s COVID-19 forecast hub in Supplementary Information (Table S3, S4).

### Comparisons of national COVID-19 hospitalizations predictions

National one-week-ahead and two-week-ahead predictions of new hospitalizations were generated using (i) ARGO inspired model, (ii) persistence (naive) model and (iii) AR7 model. The naive method simply uses past week’s hospitalizations as current week’s forecasts, without any modeling effort. AR7 is an autoregressive model of lag 7 (implemented in R package forecast^18^). For fair comparisons, all models were trained on a 56-day rolling window basis. Retrospective out-of-sample predictions of daily national hospitalizations were made every week from January 4, 2021 to December 27, 2021 by the three models and were then aggregated into weekly new hospitalizations.

Table 1 summarizes the national 1-week-ahead and 2-week-ahead predictions performance from January 4, 2021 to December 27, 2021. During this period, ARGO outperforms all the benchmark models in every error metric for both one and two weeks ahead predictions. Specifically, for the national 1-week-ahead predictions, ARGO performs better than the best alternative method by around 27% in RMSE, 39% in MAE and 1.5% in Cor. The 2-week-ahead ARGO forecasts have slightly lower error reduction in RMSE and MAE, and higher increase in Cor. SI Table S3 has additional national level comparison results with more recent state-of-the-art methods. The results demonstrate that ARGO is able to produce accurate and robust retrospective out-of-sample national 1-week-ahead and 2-week-ahead hospitalizations predictions during the evaluation period.

Figure 1 displays the 5 compared methods’ estimations. During the 12-month comparison period, ARGO can accurately capture the overall trend as well as local fluctuations (the spike between April, 2021 and May, 2021) in new hospitalizations. All of the time series forecasting methods exhibit some delaying behaviors to various degree, due to the input feature of the lagged information. Fortunately, by utilizing Google search information, ARGO is able to overcome such delayed behavior and is the only method that captures the hospital admission peaks around April 2021 and September 2021 as well as the surge around December 2021 possibly caused by omicron for both 1 and 2 weeks ahead predictions. Moreover, by leveraging time series information and the Internet search information, ARGO is able to avoid sudden spikes and drops in the prediction. ARGO is also self-correcting and can quickly recover from the over-shooting behavior which can be observed in Fig. 1b from August, 2021 to October, 2021 when ARGO corrects itself from the prediction peak in September 2021.

### Comparisons of state-level COVID-19 hospitalizations

We also conducted retrospective out-of-sample 1 and 2 weeks ahead predictions for the 51 states in the US (including Washington DC) during the same period of January 4, 2021 to December 27, 2021.

Table 2 summarizes the average error metrics of the comparing methods’ state-level predictions from January 4, 2021 to December 27, 2021. For the 1-week-ahead predictions, ARGO is able to achieve uniformly best performance in all error metrics. Compared with the two benchmark models from COVID-19 forecast hub, ARGO yields roughly 35% error reduction in RMSE, around 30% error reduction in MAE and around 2% increase in Pearson correlation coefficient. For the 2-week-ahead predictions, ARGO achieves around 12% error reduction in RMSE, approximate 8% error reduction in MAE and around 1% increase in Pearson correlation coefficient compared with COVID-19 forecast hub benchmark methods (Table 2). The full comparison of state-level hospitalizations predictions is shown in SI Table S4, and the comparison tables and figures for each individual state are available in SI Table S6-S56 and Figure S3-S53. Overall, ARGO gives the leading performance in state-level forecasts compared with the benchmark models and other state-of-the-art methods (Table S4), by efficiently utilizing relevant public search information and incorporating cross-state cross-region information as model features. Figure 2 further demonstrates the accuracy and robustness of ARGO in state-level hospitalizations predictions. The violin charts, which present the distributions of each model’s predictions errors in all three error metrics, show that the 1 standard deviation ranges of ARGO are the smallest in RMSE and MAE, and are the second best in Cor.

### Sensitivity analysis

To further analyze the importance of four features used in ARGO (hospitalizations, COVID-19 confirmed cases, percentages of vaccinated population and Google search queries), we conduct sensitivity analysis by excluding one feature at a time in state-level predictions. Table S5 shows the comparison between ARGO (full model) and each sub-model (excluding one feature) in the three error metrics for 1 and 2 weeks-ahead state-level predictions. Our full model ARGO outperforms all other simplified models throughout the forecasting horizon. In particular, the model that excludes Google search terms has relatively higher MSE and MAE compared against other submodels, suggesting the importance of Google search terms in the method. Also, Google search information alone is not predictive enough, as all other submodels (with Google search information) cannot beat ARGO (full model) in any of the error metrics, indicating the importance of information combination.

## Discussion

The ARGO inspired model combines autoregressive COVID-19 related information and online search data. It is able to produce accurate, reliable real-time hospitalizations predictions at both national and state-level for 1–2 weeks ahead predictions. The state-level real-time hospitalizations predictions made by ARGO could help local public health officials make timely allocation decisions of healthcare resources, such as ventilator, ICU beds, personal protective equipment, personnel, etc, as well as account and promptly prepare for future surges of COVID-19 pandemics caused by new virus variants. Moreover, ARGO allows us to transparently understand how Google search information and historical COVID-19 information complement one another. If any of the COVID-19-related information and Google search information is missing, the error of ARGO’s prediction will increase as shown in Table S5 in SI, indicating all features help improve predictions’ accuracy. Past week’s COVID-19 hospitalization contributes positively to the current national COVID-19 hospitalization trend predictions (Figure S2a and S2b), while the effect of past week’s vaccination rate is negative, which indicates that the current trend is likely to follow from the past week’s growth/drop, and vaccination rates potentially lead to drops in hospitalization. Figure S2a and S2b indicate a time-varying relationship between COVID-19 hospitalization trend and people’s search behavior for COVID-19 related terms since coefficients of Google search queries are changing over time. Furthermore, our ARGO hospitalization prediction model is a straightforward adaptation from the original ARGO model for influenza^9^, which reduces the chance of overfitting and again demonstrates ARGO’s robustness and flexibility.

Although ARGO shows strong performance in hospitalizations forecasts, its accuracy is controlled by the reliability of the inputs. Google search data can be noisy due to the instability of Google Trends’ sampling approach and public fear. Especially for state-level Google search data, the lack of search intensity can make the search data unrepresentative of the real interest of the people. Luckily, the IQR filter^19^ and moving average smoothing applied to Google search data are able to minimize the risk caused by such noisiness, and help ARGO produce robust output. To further account for the instability in the state-level Google search queries, the query terms are identified using the national level data where the search frequencies are more representative with lower noise and higher stability. ARGO selects the most representative search queries according to their Pearson correlation coefficients with hospital admission. In addition, the national level search frequency is directly used as input features for state-level predictions. An optimal delay between the search data and the hospital admissions is also identified for each query term. All together, ARGO is able to overcome the sparsity issues of Google search queries and produce robust future estimations (Fig. 1) while avoiding over-fitting.

Another challenge in using online search data to estimate hospitalizations is that the predictive information in Google search data die down as forecasts horizon expands (shown in Tables 1 and 2). In our results of COVID-19 hospitalizations predictions, the optimal lags (delays) of some Google search terms are relative small shown in Table 3 which indicates those Google search queries are more effective for short-term prediction of hospitalizations. Nevertheless, by leveraging COVID-19 related time series information, ARGO is able to adjust the focus between the time series and the Internet search information features when forecast horizon extends, thanks to the L1-norm penalty and the dynamic training that selects the most relevant Google search terms.

By effectively combining the Google search data and COVID-19 related time series information, ARGO has a stable model structure that is able to make accurate and robust national and state level 1–2 weeks ahead hospitalizations predictions. The simple structure of ARGO makes it universal adaptable to other COVID-19 related forecasts. With its simplicity and strengthened accuracy over other benchmark methods, ARGO could help public health decision making for the local monitoring and control of COVID-19 to better prepare for future surges of hospitalizations, patients in ICU, deaths and cases caused by new COVID-19 variants such as alpha, delta, omicron, etc.

### Concluding remarks

In this paper, we demonstrate that influenza prediction methods using online search data^9^ can be adapted for COVID-19 prediction, demonstrating their robustness and strong applicability. Specifically, by combining Google search information and COVID-19 related time-series information, we are able to accurately predict 1 and 2-week-ahead national and state-level hospitalization, achieving competitive performance against the best alternative state-of-the-art methods submitted to CDC. The combination of COVID-19 cases, vaccination rates, hospitalization, and optimally delayed Google search information are the key factors to ARGO’s accuracy and robustness in national and state-level predictions, demonstrating great potential to predict future surges and help with healthcare interventions.

## Data and methods

We focused on national hospital admission predictions and state-level predictions of 51 states in the United States, including Washington DC. The inputs consist of confirmed incremental cases, percentage of vaccinated population, confirmed new hospital admissions, and Google search query frequencies. Both state-level data and national data were directly obtained from respective data sources outlined in this section. Our prediction method is inspired by ARGO^9^, with details presented in this section as well.

### Data/code availability

All data used in this study are publicly available from respective data sources outlined here. For completeness, the datasets analysed during the current study and the code are also deposited in the Harvard dataverse repository, DOI: 10.7910/DVN/S7HOTD. All analyses, including the generation of all figures, were performed with the R statistical software^20^, version 4.1.1 (https://www.R-project.org/).

### COVID-19 related data

We used reported COVID-19 confirmed incremental cases from JHU CSSE data^21^, percentage of fully vaccinated population from Centers for Disease Control and Prevention (CDC)^22^ which is averaged among percentages of fully vaccinated population in all states and COVID-19 confirmed new hospital admissions from HHS^17^. The data sets were collected from July 15, 2020 to January 15, 2021.

**Table 1:** National level comparison error metrics.

Methods	RMSE		MAE		Cor	
	1 week ahead	2 weeks ahead	1 week ahead	2 weeks ahead	1 week ahead	2 weeks ahead
ARGO	**4667.552**	**14010.648**	**2896.857**	**9435.759**	**0.988**	**0.906**
AR7	6408.431	15750.873	4726.405	10957.369	0.973	0.834
COVIDhub-ensemble^3^	9578.408	16327.146	6301.981	10856.115	0.942	0.851
COVIDhub-baseline^3^	10442.092	19210.623	7167.538	13183.231	0.916	0.738
Naive	10528.225	19831.180	7033.212	13633.750	0.918	0.732

Error metrics of national 1-week-ahead and 2-week-ahead new hospitalizations predictions. The best scores are highlighted with boldface. All comparisons are based on the original scale of hospitalizations released by HHS. Methods are sorted by their average RMSE of 1-week-ahead and 2-week-ahead predictions. On average, the ARGO model outperforms the best alternative methods by approximately 18% in RMSE, 25% in MAE and 4% in Cor. Overall, ARGO has better predictions than all the benchmark methods during our comparison period.

**Table 2:** State-level comparison error metrics.

Methods	RMSE		MAE		Cor	
	1 week ahead	2 weeks ahead	1 week ahead	2 weeks ahead	1 week ahead	2 weeks ahead
ARGO	**170.960**	**374.330**	**114.98**	**243.765**	**0.960**	**0.879**
AR7	193.424	399.253	134.454	276.255	0.951	0.849
COVIDhub-ensemble^3^	259.348	427.926	161.523	265.042	0.937	0.867
Naive	265.789	469.206	179.084	322.021	0.934	0.825
COVIDhub-baseline^3^	296.963	482.160	193.150	320.048	0.862	0.751

Error metrics of state-level 1-week-ahead and 2-week-ahead new hospitalizations predictions, averaging across the states. The best scores are highlighted with boldface. All comparisons are based on the original scale of hospitalizations released by HHS. Methods are sorted based on their average RMSE of 1-week-ahead and 2-week-ahead predictions. ARGO outperforms the best alternative method by approximately 8% in RMSE, 12% in MAE and 1% in Cor. Overall, ARGO is the best-performing prediction model compared with other listed models.

### Google search data

Google Trends provides estimated Google search frequency for the specified query term^23^. We obtained online search data from Google Trends^23^ for the period from July 15, 2020 to January 15, 2021. To retrieve the time series search frequencies of a desired query, one needs to specify the query’s geographical information and time frame on Google Trends. The returned frequency from Google Trends is obtained by sampling all raw Google search frequencies that contain this query^23^. The detailed data collection procedure and subsequent data pre-processing (introduced in sections below) are illustrated in the flowchart (Figure S1). In step 1 (green highlighted boxes in Figure S1), to curate the pool of potentially predictive queries, we first started with 129 influenza (flu) related queries based on prior studies^9,24,25^. Then, we changed “Influnza” and “Flu” keywords to “Coronavirus” and “COVID-19”, respectively. We also added in additional COVID-19 specific search terms from Google Trends Coronavirus Story page^26^. Lastly, for each query, we also included its top “related queries and topics” based on Google Trends website^23^. Finally, we end up with 256 COVID-19 related queries (Table S1). Next two sections will describe the subsequent data cleaning and pre-processing in detail, illustrated in step 2 and step 3 of Figure S1.

#### Inter-quantile range (IQR) filter and optimal lags for Google search data

The raw Google search frequencies obtained from Google Trends^23^ are observed to be unstable and sparse^19^. Such instability and sparsity can negatively affect prediction performance of linear regression models which are sensitive to outliers. To deal with such outliers in Google search data, we used an IQR filter^19^ to remove and correct outliers on a rolling window basis. The search data that is beyond 3 standard deviation from the past 7-day average are examined and removed^19^, which is also shown in step 2 (orange highlighted box) first sub-step in Figure S1.

**Figure 4:**
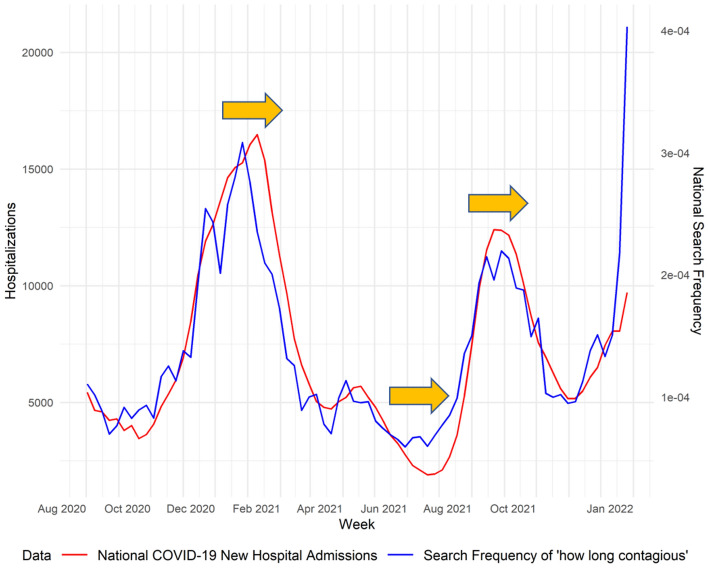
Google search query “how long contagious” and COVID-19 weekly new hospitalizations Illustration of delay in peak between Google search query search frequencies (how long contagious in blue) and COVID-19 national level weekly new hospitalizations (red). Y-axis are adjusted accordingly.

The trends of Google search frequencies are often a few days ahead of hospitalizations, indicating that the search data might contain predictive information about hospitalizations. Figure 4 demonstrates the delay behavior between Google search query frequencies and national hospitalizations. To fully utilize the predictive information in national Google search terms, we found and applied optimal lags^19^ to filtered Google search frequencies to match the trends of national hospitalizations. For each query, a linear regression of COVID-19 new hospitalizations is fitted against lagged Google search frequency, considering a range of lags (from 4 to 35). The lag results in lowest mean square error is selected as the optimal lag for that query. The data used to find optimal lags are from August 1, 2020 to December 31, 2020, and this is shown in the second sub-step in step 2 (Figure S1).

#### Selection of google search terms

After applying optimal lags to the 256 COVID-19 related terms, we further selected the queries that have correlation coefficients with national COVID-19 hospitalizations larger than 0.5 for the period from August 1, 2020 to December 31, 2020. We applied 7-day moving average to further smooth out weekly fluctuations in the selected Google search queries. All three procedures above are shown in step 3 in Figure S1, serving as the final step of the entire data pre-processing procedure. Table 3 shows the 11 selected important terms as well as their optimal lags. Table S2 displays the correlation coefficients of the optimally delayed 11 important Google search queries. Table 3 supports the intuition that when people get infected, they would search for general query like “symptoms of the covid-19” first as this query has relatively large optimal lag. After the symptoms develop, people might begin to search for specific symptoms such as “loss of smell” which has relatively smaller optimal lag. Seemingly similar query terms can have slightly different search patterns, and thus different optimal lags, as discussed in the “Descriptive analysis of the input data” section.

**Table 3:** Optimal lags of selected important terms.

Selected Google search term	Optimal lag (in days)
How long covid-19	4
Contagious coronavirus	4
Coronavirus vaccine	4
How long contagious	4
Loss of smell	4
Cough	7
Pneumonia	7
Covid-19 vaccine	7
Symptoms of the covid-19	7
Loss of taste	9
Sinus	21

### ARGO inspired prediction

Let ${\hat{y}}_{t,r}$ be the daily hospital admissions of region *r* on day *t*; $X_{k,t}$ be the Google search data of term *k* on day *t*; $c_{t,r}$ be the JHU COVID-19 incremental confirmed cases on day *t* of region *r*; $v_{t,r}$ be the cumulative percent of people who get vaccinated by day *t* of region *r*; ${\mathbb {I}}_{\{t, d\}}$ be the weekday indicator for day *t* (i.e. ${\mathbb {I}}_{\{t, 1\}}$ indicates day *t* being Monday). Standing on day T, to predict *l*-day-ahead hospital admission of state *r*, ${\hat{y}}_{T+l,r}$, we used penalized linear estimator as following:

1 $$\begin{aligned} \begin{aligned} {\hat{y}}_{T+l,r} = {\hat{\mu }}_{y,r}+\sum ^{I}_{i=0}{\hat{\alpha }}_{i,r}y_{T-i,r} + \sum _{j\in {J}}{\hat{\beta }}_{j,r}c_{T+l-j,r}+ \sum _{m\in {M}_{r}}{\hat{\gamma }}_{m,r}y_{T,m}+ \sum _{q\in {Q}}{\hat{\phi }}_{q,r}v_{T+l-q,r}+ \sum ^{K}_{k=1}{\hat{\delta }}_{k,r}X_{k,T+l-{\hat{O}}_k} + \sum ^6_{d=1}{\hat{\tau }}_{d,r}{\mathbb {I}}_{\{T+l, d\}} \end{aligned} \end{aligned}$$

where $I=6$ considering consecutive one week lagged daily hospital admissions; $J=\max \left( \{7,28\},l\right)$, considering lagged confirmed cases; ${M}_{r}$ is the set of geographical neighboring states of state *r*; $Q=\max \left( 7,l\right)$, considering vaccination data lagged by one week; ${\hat{O}}_k=\max \left( O_k,l\right)$ is the adjusted optimal lag for term *k*; $K=11$, considering 11 selected Google search terms. The coefficients for *l*-day-ahead predictions of region *r*, $\{\mu _{y,r},\varvec{\alpha }=(\alpha _{1,r},\ldots ,\alpha _{6,r}), \varvec{\beta }=(\beta _{1,r}, \ldots , \beta _{|J|,r}), \varvec{\gamma }=(\gamma _{1,r},\ldots ,\gamma _{|{{M}_{r}}|,r}), \varvec{\phi }=\phi _{max(7,l),r}, \varvec{\delta }=(\delta _{1,r},\ldots ,\delta _{11,r}), \varvec{\tau }=(\tau _{1,r}, \ldots , \tau _{6,r})\}$, were computed by

2 $$\begin{aligned} \begin{aligned} \underset{\mu _{y,r},\varvec{\alpha },\varvec{\beta },\varvec{\gamma },\varvec{\phi },\varvec{\delta },\varvec{\tau },\varvec{\lambda }}{\mathrm {argmin}} \sum _{t=T-M-l+1}^{T-l}&\omega ^{T-l-t+1}\Bigg ( y_{t+l,r}-\mu _{y,r} - \sum ^{6}_{i=0}{\alpha }_{i,r}y_{t-i,r}-\sum _{j\in {J}}{\hat{\beta }}_{j,r}c_{t+l-j,r}-\sum _{m\in {M}_{r}}{\hat{\gamma }}_{m,r}y_{t,m}\\ \;\;\;&- \sum _{q\in {Q}}{\hat{\phi }}_{q,r}v_{t+l-q,r} -\sum ^{5}_{k=1}{\hat{\delta }}_{k,r}X_{k,t+l-{\hat{O}}_k} - \sum ^6_{d=1}{\hat{\tau }}_{d,r}{\mathbb {I}}_{\{t+l, d\}}\Bigg )^2\\ \;\;\;&+ \lambda _\alpha \Vert \varvec{\alpha }\Vert _1+\lambda _\beta \Vert \varvec{\beta }\Vert _1+\lambda _\gamma \Vert \varvec{\gamma }\Vert _1+ \lambda _\phi \Vert \varvec{\phi }\Vert _1+\lambda _\delta \Vert \varvec{\delta }\Vert _1 +\lambda _\tau \Vert \varvec{\tau }\Vert _1 \end{aligned} \end{aligned}$$

M = 56 which is the length of our training period; $\omega = 0.8$ is the exponentially time-decaying weight which assigns higher weight on more recent observation. Region $\varvec{r}$ consists of U.S. and its 51 states, including Washington DC. For US national level training, the hospitalizations of neighboring states, $y_{t,m}$, and their coefficients, $\varvec{\gamma }$, are excluded. To address the sparsity of Google search data, we used penalty of L1-norm. For simplicity, the hyperparameters $\varvec{\lambda }=(\lambda _{\alpha },\lambda _{\beta },\lambda _{\gamma },\lambda _{\phi },\lambda _{\delta },\lambda _{\tau })$ for L1-norm penalty were set to be equal and obtained via 10-folds cross-validation.

With the formulation above, on each Monday from January 4, 2021 to December 27, 2021, we iteratively trained our model and made national and state-level retrospective out-of-sample hospitalizations predictions up to 14 days into future. We then aggregated daily predictions into one-week-ahead and two-week-ahead predictions. For example, ${\hat{y}}_{T+1:T+7,r}=\sum ^7_{i=1}{\hat{y}}_{T+i,r}$ and ${\hat{y}}_{T+8:T+14,r}=\sum ^{14}_{i=8}{\hat{y}}_{T+i,r}$ are the 1-week-ahead prediction and 2-week-ahead prediction on day *T* of region *r*, respectively.

### Evaluation metrics

Root Mean Squared Error (RMSE) between a hospitalization estimate ${\hat{y}}_t$ and the true value $y_t$ over period $t=1,\ldots , T$ is $\sqrt{\frac{1}{T}\sum _{t=1}^T \left( {\hat{y}}_t - y_t\right) ^2}$. Mean Absolute Error (MAE) between an estimate ${\hat{y}}_t$ and the true value $y_t$ over period $t=1,\ldots , T$ is $\frac{1}{T}\sum _{t=1}^T \left| {\hat{y}}_t - y_t\right|$. Correlation is the Pearson correlation coefficient between $\hat{\varvec{y}}=({\hat{y}}_1, \dots , {\hat{y}}_T)$ and $\varvec{y}=(y_1,\dots , y_T)$. All estimates ${\hat{y}}_t$ and the true value $y_t$ were weekly aggregated before calculating RMSE, MAE and Cor.

### Ethics approval and consent to participate

This study did not involve human participants, data, or tissue. It was conducted using only aggregated and anonymized data. Institutional review board approval was not required. All methods were carried out in accordance with relevant guidelines and regulations.

## Supplementary Information

Supplementary Information.
