# Supplementary material for: COVID-19 hospitalizations forecasts using internet search data
Source: Sci Rep. 2022 Jun 11;12:9661. doi: 10.1038/s41598-022-13162-9 (PMC9188562; doi:10.1038/s41598-022-13162-9)
Supplement: Supplementary file 1 — Supplementary Information. [file 41598_2022_13162_MOESM1_ESM.pdf]

Supplementary Information

Flowchart of Google Search Data Cleaning

Figure S1 below illustrates the entire data pre-processing procedure for Google search data. In step 1, we curate the pool of potentially predictive Google search queries for COVID-19 hospitalization. First, we started with 129 influenza (flu) related queries based on prior influenza tracking studies[9,24,25]. Then, we changed "Influnza" and "Flu" keywords to "Coronavirus" and "COVID-19", respectively, and additionally added COVID-19 specific search terms from Google Trends Coronavirus Story page[26]. Lastly, for each query, we also included its top "related queries and topics" based on Google Trends website[23], if not already included, and eventually end up with 256 COVID-19 related queries (Table S1).

In step 2, we further de-noise all 256 queries by applying the IQR filter[19], which replaces outliers with previous days' moving average on a rolling window basis. Additionally, we find each search query's optimal lag from hospitalization trend to fully utilize their predictive power. The calculation of optimal lag proceeds as follows. First, for each search term, we delay the term of interest by a fixed number of days ranging from 4 to 35, and fit a linear regression of the lagged term (ONLY) against the hospitalization trend. This will result in 31 linear regression fits for each term. Lastly, we select the lag that results in the smallest mean squared error (MSE) fit and denote it as the optimal lag for the term of interest. The linear regression's MSE evaluation period is from August 2020 to December 2020.

In step 3, we further select the important Google search terms, by computing the correlation between optimal lagged search terms and hospitalization trend and select the top 11 terms (greater than 0.5), shown in Table S2. Lastly, we apply a 7-day moving average on all selected search term frequencies to further de-noise the Google search terms.

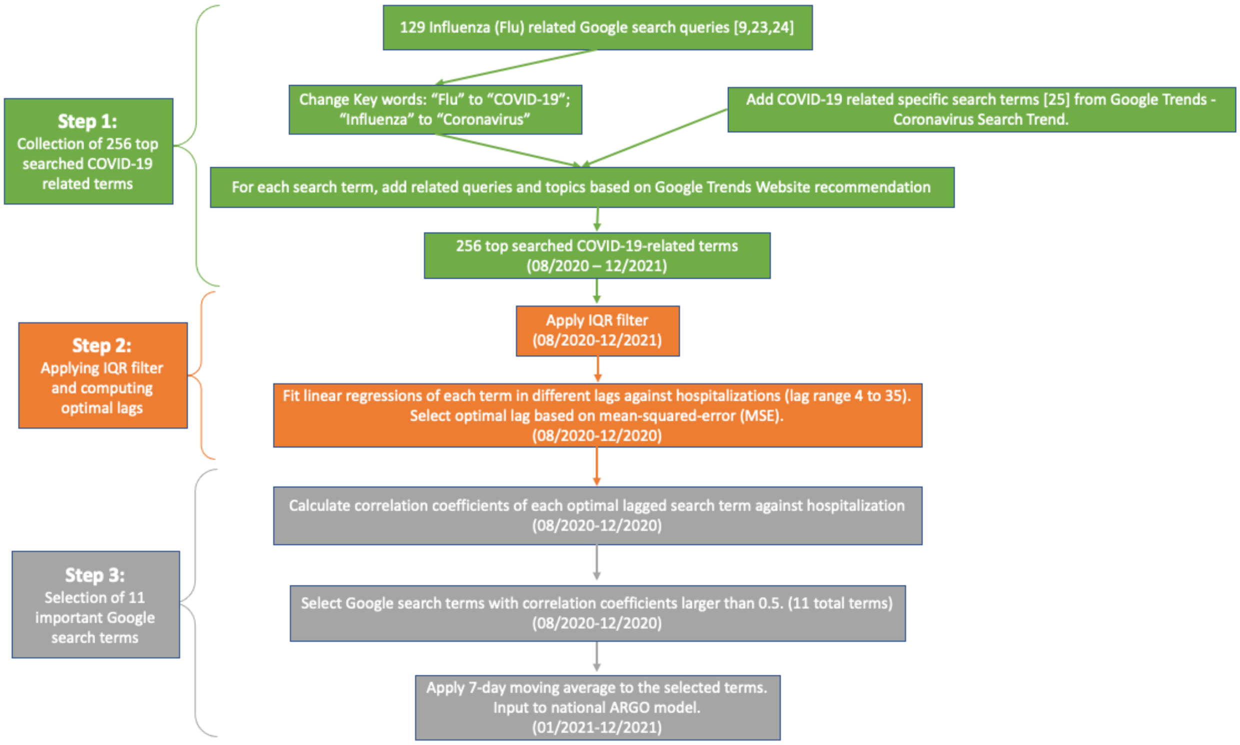

Figure S1. Flowchart of Google Search Data Collection, cleaning, and pre-processing. Illustration of how the initial large pool of Google search data are collected, cleaned (de-noised), and further selected (optimal lag and important terms selection).

256 Google Search Terms

Table S1. The full 256 COVID-19-related Google queries considered. The terms in parentheses are “topics” for Google Trends.

Google Query	Google Query	Google Query	Google Query
how long contagious	coronavirus fever	how to test covid 19	coronavirus where clinic
loss of smell	fever coronavirus	covid19 family	respiratory covid 19
loss of taste	rapid coronavirus	how to treat the coronavirus	covid 19 fever
(Cough)	cough fever	treat coronavirus	fever covid 19
(covid 19 vaccine)	fever cough	signs of the coronavirus	coronavirus contagious period
sinus	covid 19 texas	i have the coronavirus	the covid 19 virus
pneumonia	symptoms of the coronavirus	coronavirus prescription	covid 19 hotline
symptoms of the covid 19	covid 19 cases	having the coronavirus	what to do if you have the coronavirus
bronchitis	coronavirus insurance	coronavirus testing kit	coronavirus lasts
how long covid 19	early covid 19 symptoms	heal coronavirus	how to bring a fever down
contagious coronavirus	upper respiratory	covid19 hotline	incubation period for coronavirus
coronavirus contagious	expectorant	covid 19 hospital	coronavirus test center
coronavirus children	is covid 19 contagious	covid 19 death rate	(Oseltamivir)
coronavirus treatment	shortness of breath	treat the coronavirus	covid19 insurance
rapid covid 19	fever reducer	coronavirus hospitalization	coronavirus self isolation
exposed to coronavirus	covid19 children	do i have the covid 19	medicine for the coronavirus
robitussin	how long is the coronavirus	signs of the covid 19	covid 19 drive thru
coronavirus death	how to test coronavirus	braun thermoscan	(covid 19 season)
(Common cold)	symptoms of pneumonia	how long does the coronavirus last	covid 19 diagnosis
(coronavirus vaccine)	treatment for coronavirus	coronavirus incubation period	how long is the covid 19
the coronavirus	covid 19 symptoms	how long is coronavirus contagious	covid 19 vitamin c
coronavirus texas	coronavirus doctor	covid 19 insurance	coronavirus medical insurance
(Headache)	coronavirus medicine	dangerous fever	treat covid 19
coronavirus cases	(Fever)	treating the coronavirus	cold or coronavirus
(Nasal congestion)	fever	covid19 exposure	coronavirus or cold
(Virus)	covid 19 relief	medicine for coronavirus	incubation period for the coronavirus
contagious covid 19	coronavirus family	coronavirus treatments	respiratory coronavirus
covid 19 contagious	coronavirus exposure	covid 19 care	covid19 doctor
coronavirus in children	(Sore throat)	coronavirus high temperature	covid 19 medicine
strep throat	sore throat	coronavirus length	break a fever
the covid 19	covid 19 what to do	coronavirus medication	do i have the coronavirus
covid 19 death	coronavirus remedy	covid19 death rate	coronavirus test drive thru
(coronavirus)	symptoms of bronchitis	cold vs coronavirus	tussionex
coronavirus	covid 19 treatment	coronavirus vs cold	remedies for coronavirus
cure coronavirus	coronavirus recovery	cure for covid19	covid 19 recovery
strep	cure for coronavirus	covid 19 exposure	human temperature
chest cold	coronavirus runny nose	get rid of the coronavirus	coronavirus diagnosis
covid 19 children	covid19 death	normal body temperature	normal body
coronavirus cough	covid 19 doctor	fight the coronavirus	covid19 vitamin c
oscillococcinum	covid 19 report	cure covid 19	coronavirus duration
coronavirus report	incubation period for covid 19	coronavirus hotline	treatment for covid 19
covid 19 in children	how to treat coronavirus	healing coronavirus	covid19 drive thru
covid 19 test	cure the coronavirus	covid 19 medication	(Rhinorrhea)
walking pneumonia	treatment for the coronavirus	coronavirus incubation	coronavirus call number
coronavirus what to do	coronavirus reports	(Spanish covid 19)	high fever
coronavirus symptoms	sinus infections	covid 19 cough	covid19 medication
coronavirus death rate	coronavirus test kit	ear thermometer	body temperature
coronavirus test	covid 19 incubation	covid 19 hospitalization	covid 19 headache
acute bronchitis	treating coronavirus	covid 19 test drive thru	coronavirus regimen
covid 19 test kit	how long does covid 19 last	over the counter coronavirus	reduce a fever
(covid 19)	coronavirus and fever	fever breaks	how to break a fever
covid 19	is coronavirus contagious	tussin	covid 19 test center
coronavirus how long	how long does coronavirus last	flu or coronavirus	get over the coronavirus
how long coronavirus	thermoscan	how to get rid of the coronavirus	coronavirus online assessment
early coronavirus symptoms	exposed to covid 19	low body	coronavirus temperature fever
coronavirus care	(Nausea)	coronavirus germs	how long is the coronavirus contagious
coronavirus hospital	covid19 hospital	i have the covid 19	coronavirus meds
covid19 cases	covid 19 reports	coronavirus hospitalization cost	coronavirus high temperature fever
coronavirus headache	coronavirus remedies	cure for covid 19	cannot breathe
signs of coronavirus	covid 19 family	how to test covid19	new drug for coronavirus
symptoms of coronavirus	reduce fever	coronavirus vitamin c	taking temperature
symptoms of covid 19	covid 19 runny nose	cold and coronavirus	painful cough
tessalon	covid 19 incubation period	coronavirus and cold	coronavirus running nose
coronavirus relief	signs of covid 19	coronavirus drive thru	covid19 what to do

Correlation Coefficients Table of Selected Google Search Queries

Table S2 shows the selected 11 optimal lagged Google search queries' correlation coefficients with COVID-19 new hospitalization from August 1, 2020 to December 31, 2021.

Table S2. Selected 11 Optimal Lagged Important Terms' Correlation Coefficients with Hospitalizations Ranked from Largest to Smallest

Google Search Query	Correlation Coefficient
how long contagious	0.896
loss of smell	0.886
loss of taste	0.873
Cough	0.775
covid-19 vaccine	0.748
sinus	0.739
pneumonia	0.678
symptoms of the covid-19	0.663
how long covid-19	0.615
contagious coronavirus	0.584
coronavirus vaccine	0.502

Additional Retrospective Evaluations

After filtering out teams with incomplete submissions and miss-aligned prediction dates, we collected predictions of the top 5 models from COVID-19 forecast hub[3] for comparisons.

Table S3. National Level Comparison Error Metrics

Methods	RMSE		MAE		Cor	
	1 Week Ahead	2 Weeks Ahead	1 Week Ahead	2 Weeks Ahead	1 Week Ahead	2 Weeks Ahead
ARGO	**4667.551**	**14182.659**	**2896.857**	**9435.759**	**0.988**	**0.906**
AR7	6408.430	15750.873	4726.405	10957.369	0.973	0.834
MOBS-GLEAM-COVID[6]	8211.132	15909.210	6579.988	11714.361	0.960	0.856
COVIDhub-ensemble[3]	9578.408	16327.146	6301.981	10856.115	0.942	0.851
GT-DeepCOVID[4]	9179.514	17704.803	6481.583	12721.385	0.943	0.815
COVIDhub-baseline[3]	10442.092	19210.623	7167.538	13183.231	0.916	0.738
Naive	10528.225	19831.180	7033.212	13633.750	0.918	0.732
JHUAPL-Bucky[7]	12393.690	18200.825	9121.523	12944.933	0.915	0.877

Error metrics of national one-week-ahead and two-weeks-ahead new hospitalizations predictions. The best scores are highlighted with boldface. All comparisons are based on the original scale of hospitalizations released by HHS. Methods are sorted by their average RMSE of one-week-ahead and two-weeks-ahead predictions. CDC forecast hub collects predictions from every team that submits their weekly predictions, which all have their strengths in different time periods. On average, the ARGO model outperforms the benchmark methods (Naive, AR7, COVIDhub-ensemble,COVIDhub-baseline) by approximately 18% in RMSE, 25% in MAE and 4% in Cor. Overall, ARGO has better predictions than all other models during our comparison period.

Table S4. State-Level Comparison Error Metrics

Methods	RMSE		MAE		Cor	
	1 Week Ahead	2 Weeks Ahead	1 Week Ahead	2 Weeks Ahead	1 Week Ahead	2 Weeks Ahead
ARGO	**170.960**	**374.33**	**114.98**	**243.765**	**0.960**	**0.879**
AR7	193.424	399.253	134.454	276.255	0.951	0.849
COVIDhub-ensemble[3]	259.348	427.926	161.523	265.042	0.937	0.867
Naive	265.789	469.206	179.084	322.021	0.934	0.825
MOBS-GLEAM-COVID[6]	281.464	467.701	195.785	314.702	0.910	0.809
COVIDhub-baseline[3]	296.963	482.16	193.150	320.048	0.862	0.751
GT-DeepCOVID[4]	282.921	497.770	197.385	344.886	0.900	0.778
JHUAPL-Bucky[7]	458.423	636.001	291.534	408.232	0.874	0.811

Error metrics of state-level one-week-ahead and two-weeks-ahead new hospitalizations predictions, averaging across the states. The best scores are highlighted with boldface. All comparisons are based on the original scale of hospitalizations released by HHS. Methods are sorted based on their average RMSE of one-week-ahead and two-weeks-ahead predictions. On average, the ARGO model outperforms the best alternative method by approximately 18% in RMSE, 25% in MAE and 4% in Cor. Overall, ARGO has better prediction performance than other listed methods during our comparison period.

ARGO Model Sensitivity to Each Feature Included

Table S5. State-Level Comparison Error Metrics of Sub-models

Methods	RMSE		MAE		Cor	
	1 Week Ahead	2 Weeks Ahead	1 Week Ahead	2 Weeks Ahead	1 Week Ahead	2 Weeks Ahead
ARGO	**170.960**	**374.330**	**114.98**	**243.765**	**0.960**	**0.879**
ARGO-Excluding-Cases	174.052	374.344	116.843	245.255	0.958	0.871
ARGO-Excluding-Vaccination	173.246	378.599	117.944	252.378	0.958	0.872
ARGO-Excluding-GT	180.536	384.037	118.845	248.010	0.956	0.868
ARGO-Excluding-Hospitalization	212.337	408.397	138.030	263.469	0.945	0.857
Naive	265.789	469.206	179.084	322.021	0.934	0.825

Error metrics of state-level one-week-ahead and two-weeks-ahead new hospitalizations predictions for the period from January 4, 2021 to December 27, 2021 of four sub-models (ARGO-Excluding-Hospitalization, ARGO-Excluding-Vaccination, ARGO-Excluding-Cases and ARGO-Excluding-GT where GT denotes Google search terms), averaging across the states. The best scores are highlighted with boldface. All comparisons are based on the original scale of hospitalizations released by HHS. Methods are sorted based on their average RMSE of one-week-ahead and two-weeks-ahead predictions. The full model (ARGO) gives lowest error in three error metrics compared with four sub-models throughout 1 to 2 weeks ahead predictions, suggesting the four features (hospitalizations, cases, vaccinations and Google search frequencies) used in ARGO contribute positively to ARGO's predictions accuracy. The results also demonstrate the importance of Google search queries and autoregressive information of hospitalizations used in ARGO which can be regarded as two major contributing features in hospitalizations predictions of ARGO since excluding either of them will result in more than 5% error increase in RMSE (ARGO-Excluding-GT and ARGO-Excluding-Hospitalization).

ARGO model parameter heatmaps

Fig S2a and S2b, show ARGO national level 1 to 2 weeks ahead forecasts' model parameters in heatmaps.

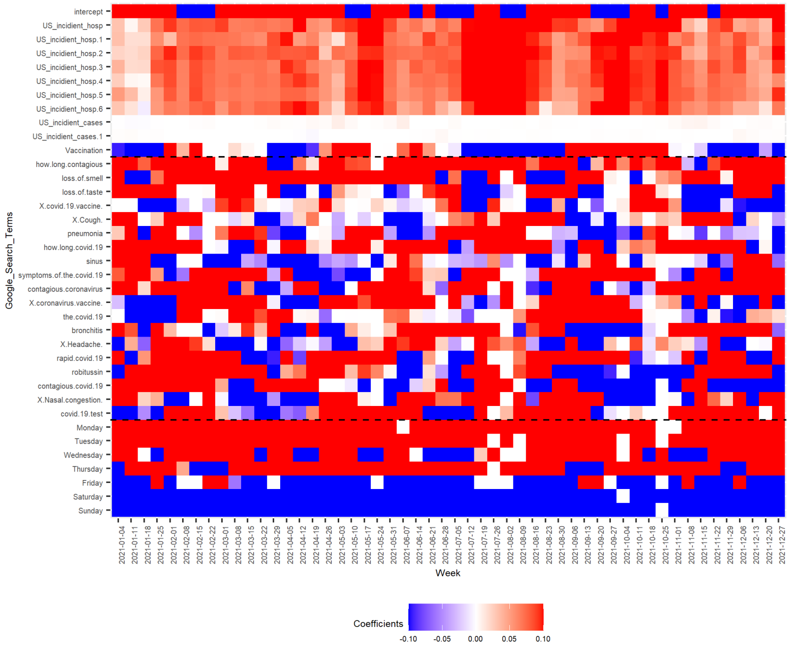

(a) 1 Week Ahead National Level ARGO Coefficients

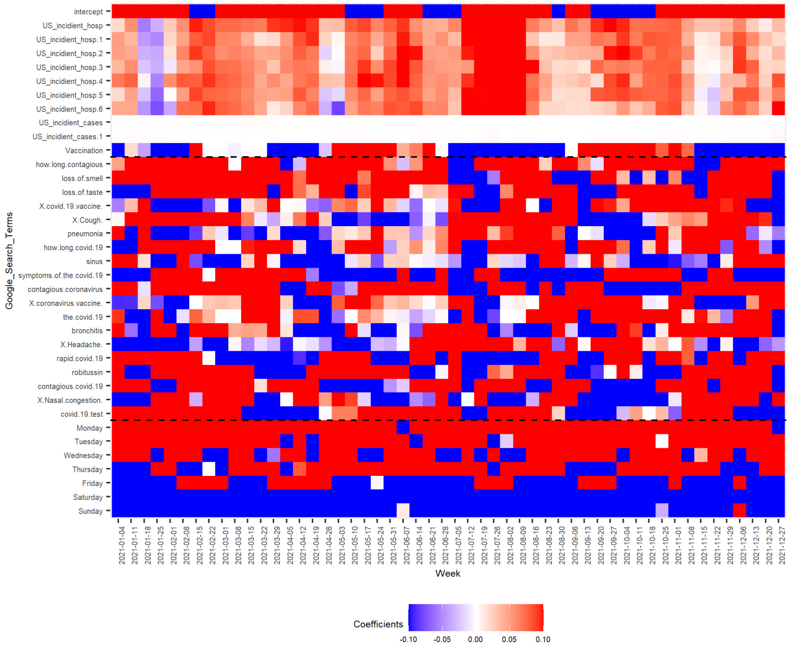

(b) 2 Weeks Ahead National Level ARGO Coefficients

Figure S2. Coefficients heat maps of ARGO national level 1 and 2 weeks ahead predictions. To clearly show magnitude of coefficients, coefficients larger or smaller than 0.1 and -0.1 are scaled to 0.1 and -0.1, respectively. Positive coefficients are filled with red color, negative coefficients are filled with blue color, and zero values are filled with white color. Google queries are separated from COVID-19 related autoregressive terms using black horizontal dashed line. The generation of the figures were performed with the R statistical software, version 4.1.1 (`https://www.R-project.org/`)

Detailed comparison for each state

Methods	RMSE		MAE		Cor	
	1 Week Ahead	2 Week Ahead	1 Week Ahead	2 Week Ahead	1 Week Ahead	2 Week Ahead
ARGO	13.333	18.005	9.921	12.438	0.881	0.799
AR7	17.197	22.752	12.477	15.208	0.802	0.673
COVIDhub-ensemble	15.310	17.608	10.365	**12.288**	0.847	0.815
Naive	**12.996**	**16.775**	**9.635**	**12.288**	**0.883**	**0.826**
MOBS-GLEAM_COVID	18.376	24.910	13.055	16.764	0.840	0.708
COVIDhub-baseline	22.075	26.171	16.558	19.596	0.688	0.597
GT-DeepCOVID	14.265	18.030	11.369	13.202	0.864	0.808
JHUAPL-Bucky	19.169	24.398	14.328	17.772	0.762	0.710

Table S6. Comparison of different methods for state-level COVID-19 1 to 2 weeks ahead hospitalizations predictions in Vermont (VT). The MSE, MAE, and correlation are reported and best performed method is highlighted in boldface.

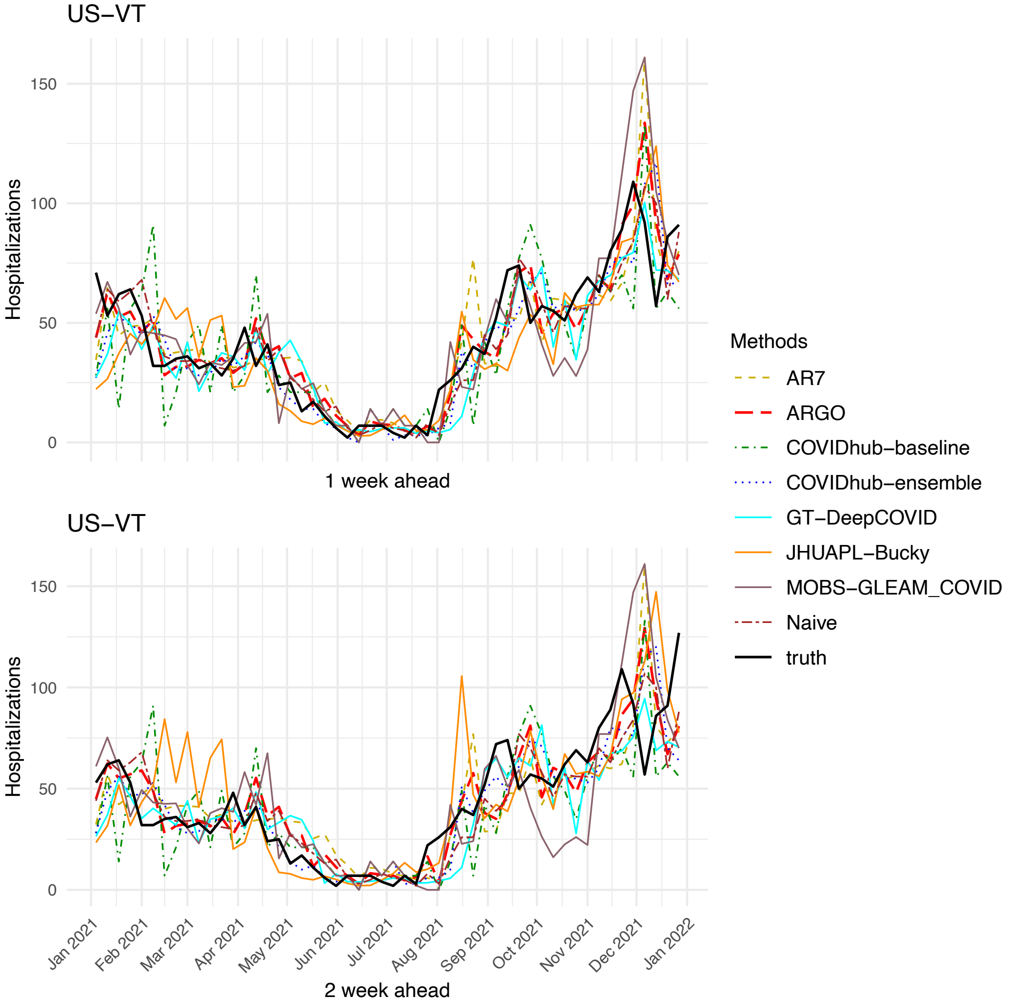

Figure S3. Plots of the COVID-19 hospitalizations 1 week (top), 2 weeks (bottom) ahead estimates of all compared models for Vermont(VT).

Methods	RMSE		MAE		Cor	
	1 Week Ahead	2 Week Ahead	1 Week Ahead	2 Week Ahead	1 Week Ahead	2 Week Ahead
ARGO	**30.019**	**46.728**	**21.430**	**35.221**	**0.958**	**0.910**
AR7	31.614	53.212	23.479	40.973	0.952	0.866
COVIDhub-ensemble	32.954	51.814	24.404	39.231	0.950	0.884
Naive	30.921	48.101	23.404	37.904	0.952	0.888
MOBS-GLEAM_COVID	43.928	60.280	32.330	45.463	0.925	0.854
COVIDhub-baseline	38.770	49.583	31.731	41.154	0.925	0.882
GT-DeepCOVID	48.603	70.530	36.624	55.702	0.880	0.765
JHUAPL-Bucky	63.595	112.865	39.121	64.859	0.911	0.829

Table S7. Comparison of different methods for state-level COVID-19 1 to 2 weeks ahead hospitalizations predictions in New Hampshire (NH). The MSE, MAE, and correlation are reported and best performed method is highlighted in boldface.

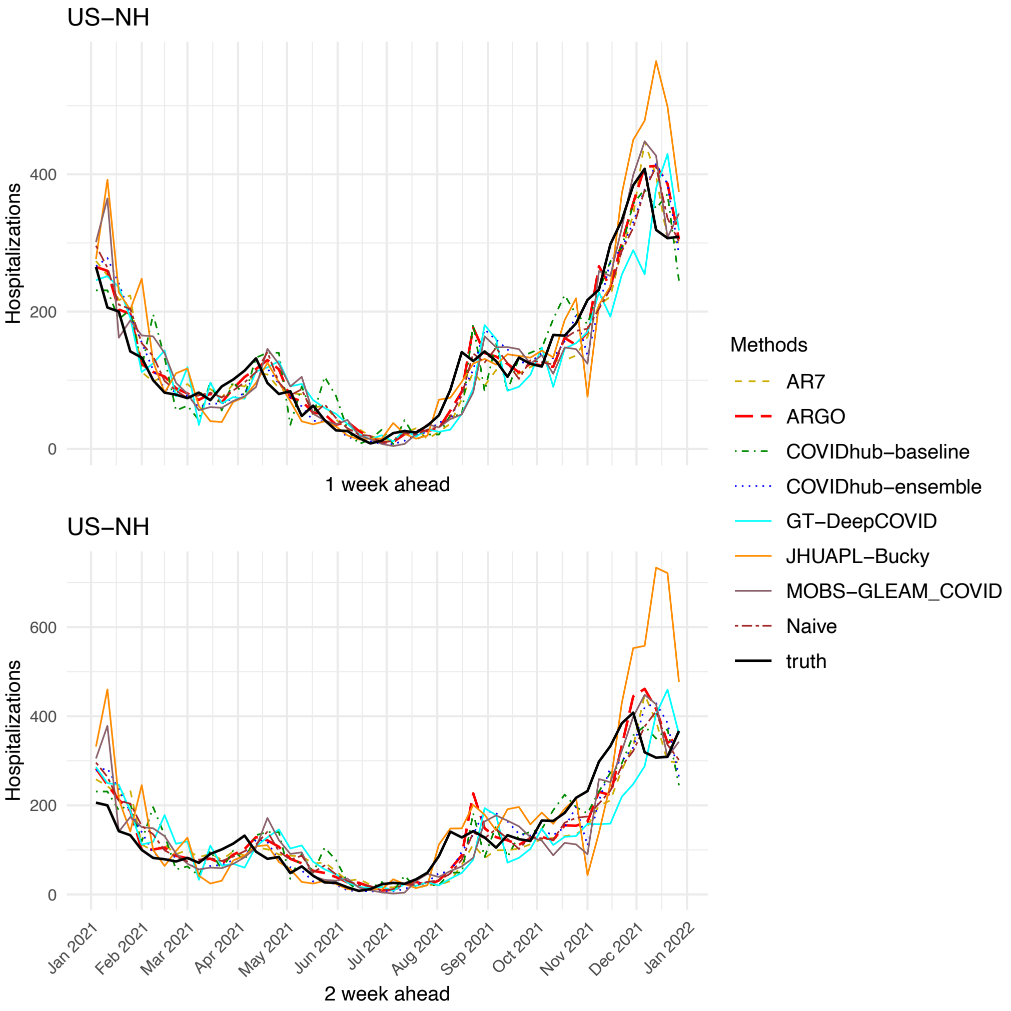

Figure S4. Plots of the COVID-19 hospitalizations 1 week (top), 2 weeks (bottom) ahead estimates of all compared models for New Hampshire (NH).

Methods	RMSE		MAE		Cor	
	1 Week Ahead	2 Week Ahead	1 Week Ahead	2 Week Ahead	1 Week Ahead	2 Week Ahead
ARGO	52.250	75.913	38.046	52.019	0.953	0.899
AR7	51.600	78.175	40.400	58.499	0.949	0.879
COVIDhub-ensemble	49.051	**70.939**	36.288	**50.788**	**0.959**	**0.921**
Naive	**48.420**	78.998	**35.385**	57.827	0.951	0.870
MOBS-GLEAM_COVID	59.286	85.498	42.706	63.731	0.933	0.865
COVIDhub-baseline	63.577	90.055	44.923	67.750	0.940	0.867
GT-DeepCOVID	58.170	86.678	45.178	63.402	0.942	0.884
JHUAPL-Bucky	66.075	126.709	47.948	86.908	0.954	0.920

Table S8. Comparison of different methods for state-level COVID-19 1 to 2 weeks ahead hospitalizations predictions in Idaho (ID). The MSE, MAE, and correlation are reported and best performed method is highlighted in boldface.

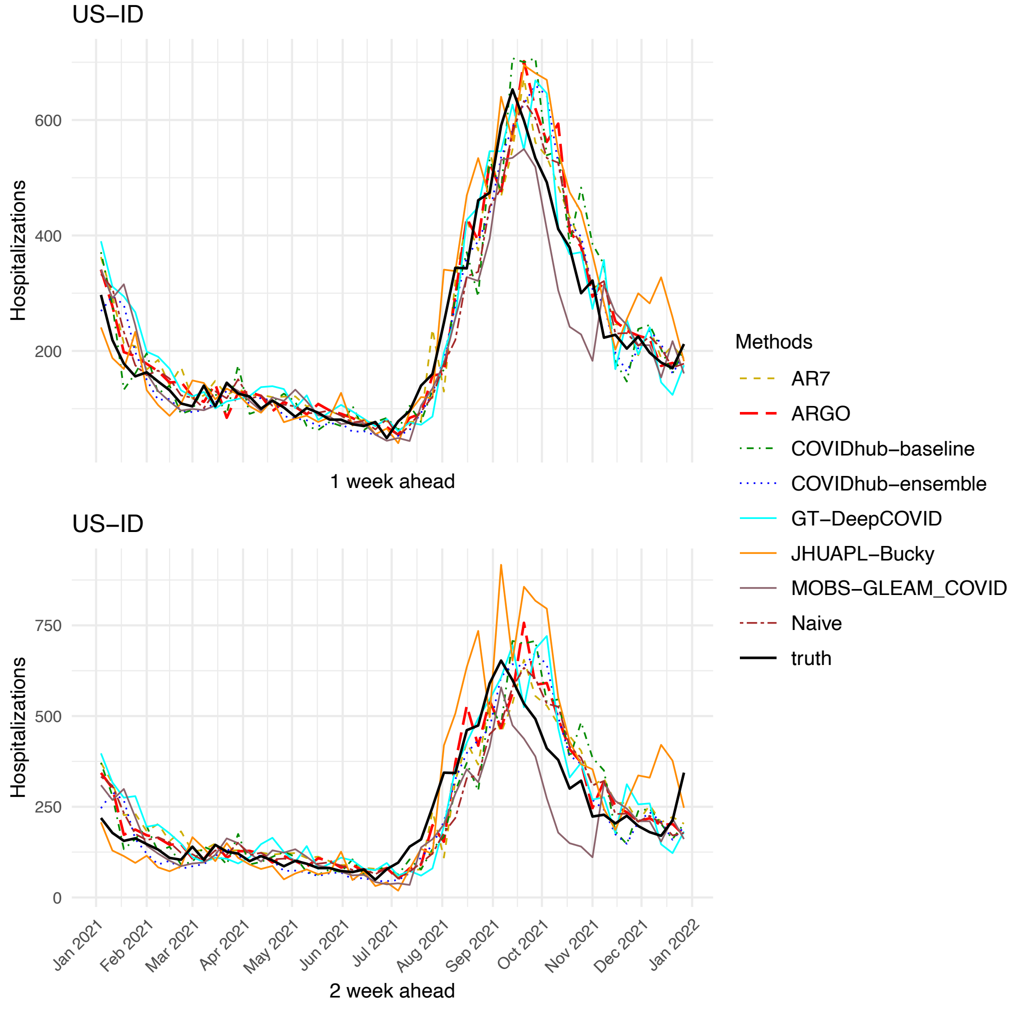

Figure S5. Plots of the COVID-19 hospitalizations 1 week (top), 2 weeks (bottom) ahead estimates of all compared models for New Idaho (ID).

Methods	RMSE		MAE		Cor	
	1 Week Ahead	2 Week Ahead	1 Week Ahead	2 Week Ahead	1 Week Ahead	2 Week Ahead
ARGO	**92.105**	228.777	**68.979**	**152.275**	**0.975**	**0.846**
AR7	98.239	**221.348**	72.824	162.710	**0.975**	0.819
COVIDhub-ensemble	210.292	362.934	125.000	203.173	0.907	0.746
Naive	143.484	272.894	96.250	180.288	0.915	0.696
MOBS-GLEAM_COVID	212.609	352.741	128.139	209.235	0.927	0.819
COVIDhub-baseline	166.796	286.944	107.558	181.442	0.879	0.652
GT-DeepCOVID	201.068	369.295	136.673	250.048	0.891	0.676
JHUAPL-Bucky	312.020	497.312	184.330	262.479	0.839	0.673

Table S9. Comparison of different methods for state-level COVID-19 1 to 2 weeks ahead hospitalizations predictions in Mississippi (MS). The MSE, MAE, and correlation are reported and best performed method is highlighted in boldface.

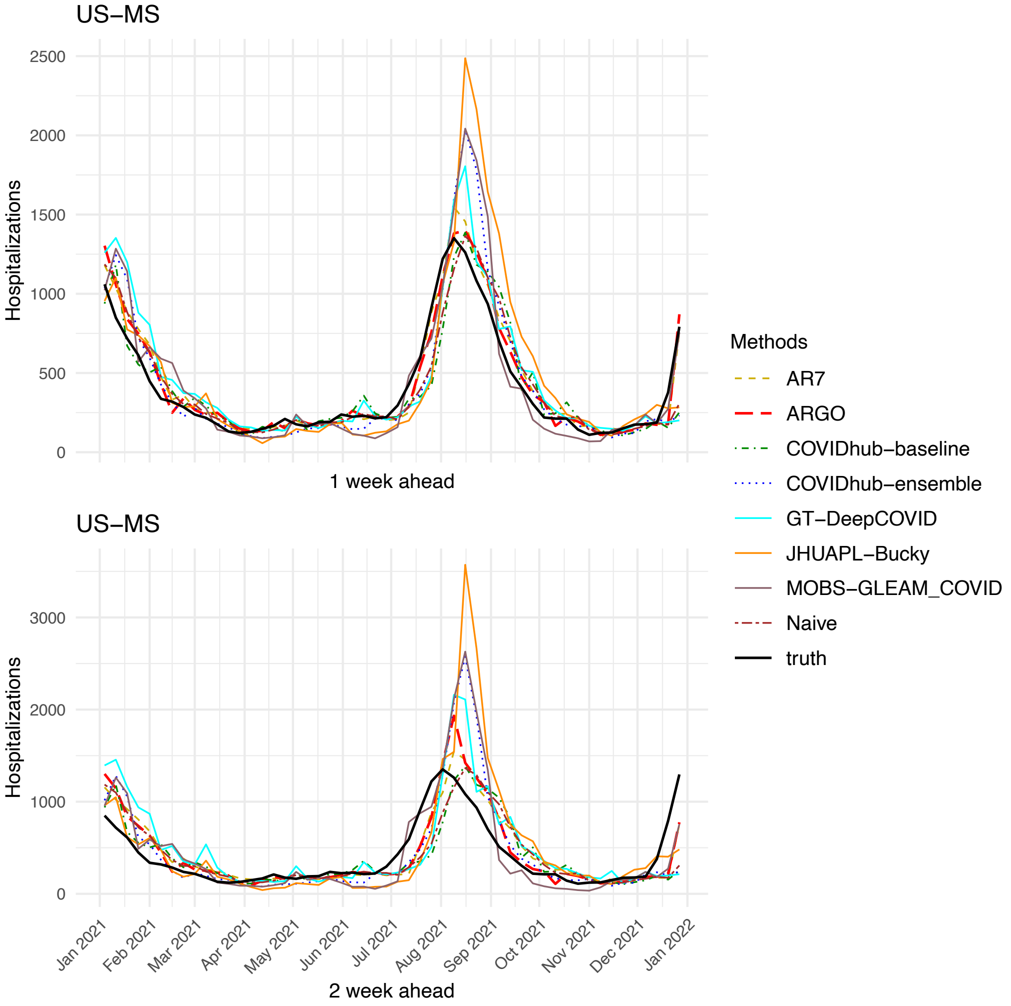

Figure S6. Plots of the COVID-19 hospitalizations 1 week (top), 2 weeks (bottom) ahead estimates of all compared models for New Mississippi (MS).

Methods	RMSE		MAE		Cor	
	1 Week Ahead	2 Week Ahead	1 Week Ahead	2 Week Ahead	1 Week Ahead	2 Week Ahead
ARGO	**27.871**	**59.532**	**19.053**	**36.003**	**0.960**	**0.844**
AR7	36.439	72.537	24.072	43.727	0.931	0.746
COVIDhub-ensemble	48.815	77.182	29.365	45.423	0.890	0.772
Naive	45.194	78.542	28.000	46.077	0.888	0.692
MOBS-GLEAM_COVID	39.931	65.546	26.723	42.401	0.915	0.796
COVIDhub-baseline	87.433	109.850	46.154	61.000	0.667	0.492
GT-DeepCOVID	39.913	69.336	28.192	45.846	0.919	0.771
JHUAPL-Bucky	44.486	70.817	31.108	46.346	0.903	0.826

Table S10. Comparison of different methods for state-level COVID-19 1 to 2 weeks ahead hospitalizations predictions in Hawaii (HI). The MSE, MAE, and correlation are reported and best performed method is highlighted in boldface.

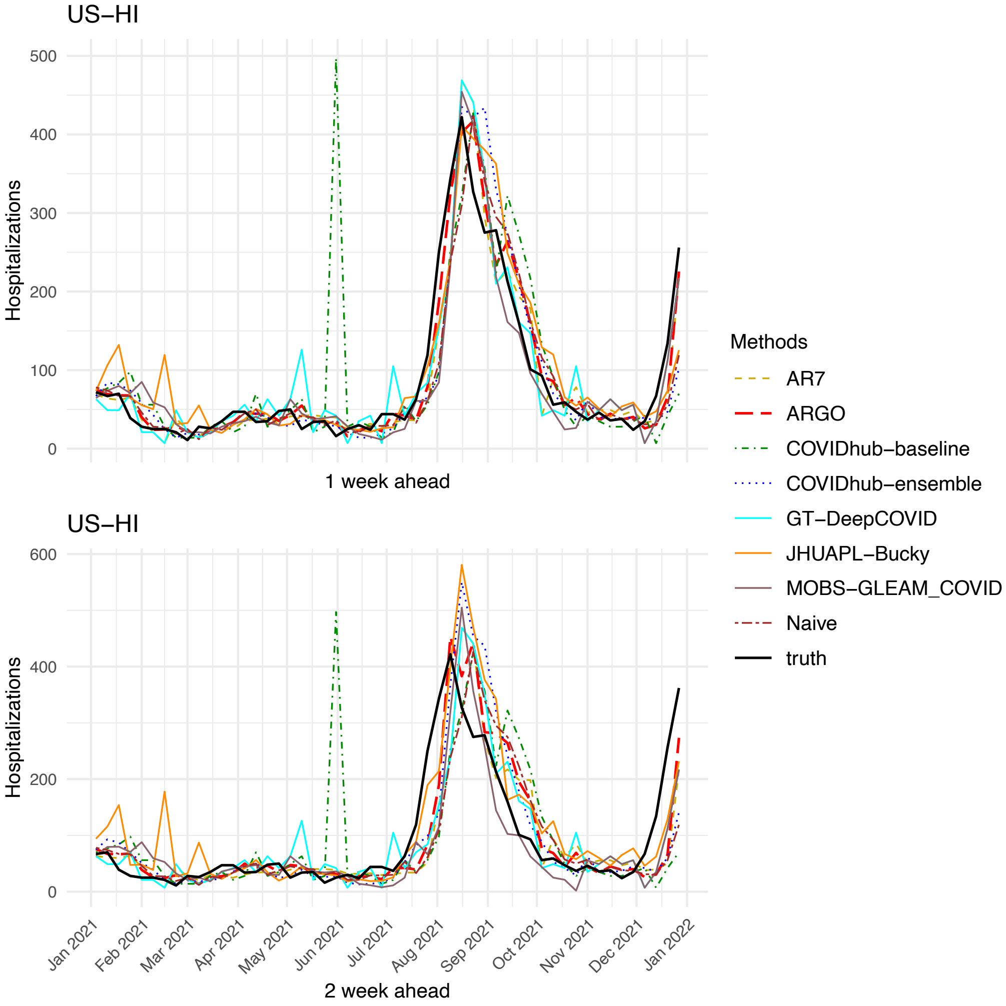

Figure S7. Plots of the COVID-19 hospitalizations 1 week (top), 2 weeks (bottom) ahead estimates of all compared models for New Hawaii (HI).

Methods	RMSE		MAE		Cor	
	1 Week Ahead	2 Week Ahead	1 Week Ahead	2 Week Ahead	1 Week Ahead	2 Week Ahead
ARGO	**84.596**	162.298	**64.685**	124.406	**0.968**	**0.904**
AR7	90.964	**149.828**	68.958	122.481	0.961	0.898
COVIDhub-ensemble	99.377	157.419	75.885	**117.404**	0.957	0.903
Naive	93.028	155.334	75.673	129.962	0.958	0.892
MOBS-GLEAM_COVID	126.640	210.765	95.340	144.185	0.936	0.836
COVIDhub-baseline	132.690	176.805	101.731	144.788	0.920	0.862
GT-DeepCOVID	154.898	247.475	114.148	188.196	0.887	0.732
JHUAPL-Bucky	249.706	421.022	146.733	250.953	0.898	0.810

Table S11. Comparison of different methods for state-level COVID-19 1 to 2 weeks ahead hospitalizations predictions in Minnesota (MN). The MSE, MAE, and correlation are reported and best performed method is highlighted in boldface.

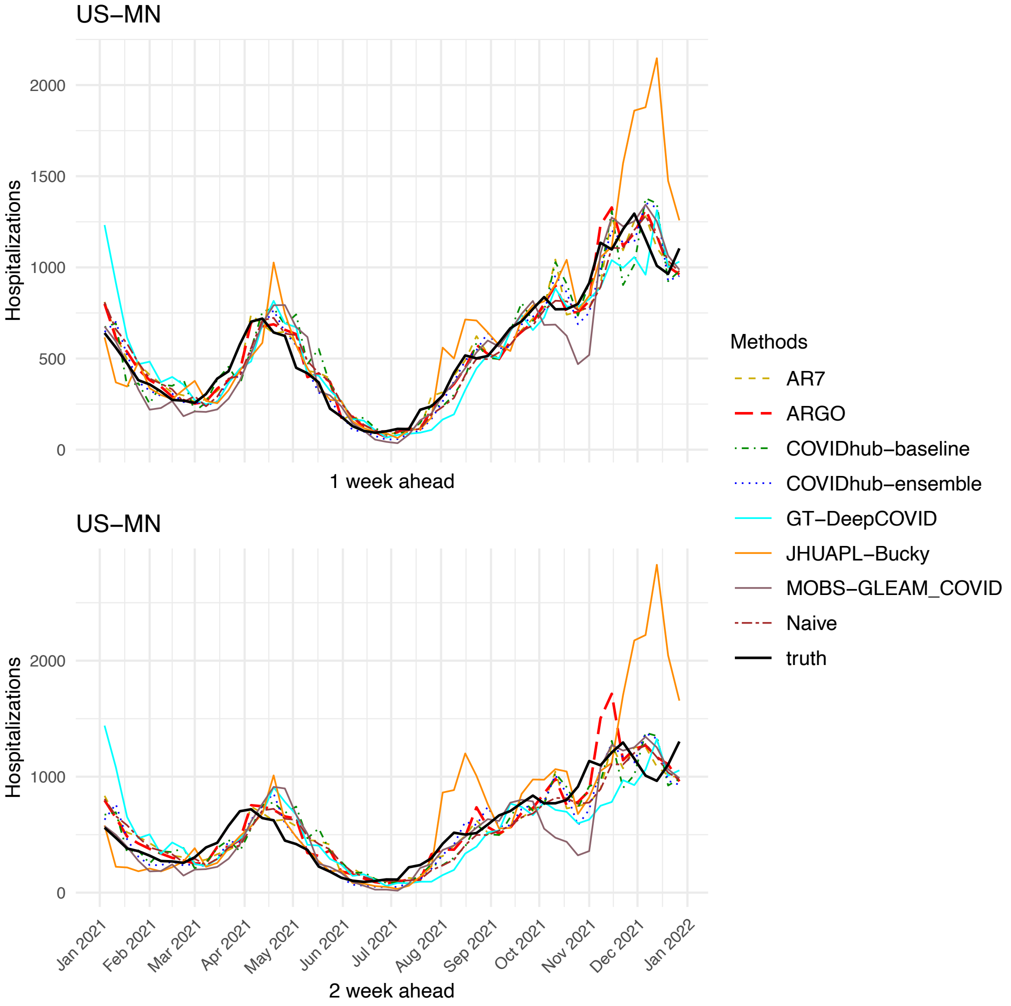

Figure S8. Plots of the COVID-19 hospitalizations 1 week (top), 2 weeks (bottom) ahead estimates of all compared models for New Minnesota (MN).

Methods	RMSE		MAE		Cor	
	1 Week Ahead	2 Week Ahead	1 Week Ahead	2 Week Ahead	1 Week Ahead	2 Week Ahead
ARGO	27.134	**33.196**	**17.813**	**25.948**	0.928	0.895
AR7	26.937	37.586	18.663	29.121	0.927	0.857
COVIDhub-ensemble	**26.631**	36.734	20.423	26.750	**0.943**	**0.898**
Naive	27.142	38.988	19.827	28.365	0.928	0.853
MOBS-GLEAM_COVID	41.629	59.711	31.201	43.233	0.872	0.767
COVIDhub-baseline	37.552	50.556	26.538	35.654	0.873	0.770
GT-DeepCOVID	35.562	51.977	26.549	37.724	0.868	0.735
JHUAPL-Bucky	48.270	73.256	27.672	42.234	0.826	0.725

Table S12. Comparison of different methods for state-level COVID-19 1 to 2 weeks ahead hospitalizations predictions in North Dakota (ND). The MSE, MAE, and correlation are reported and best performed method is highlighted in boldface.

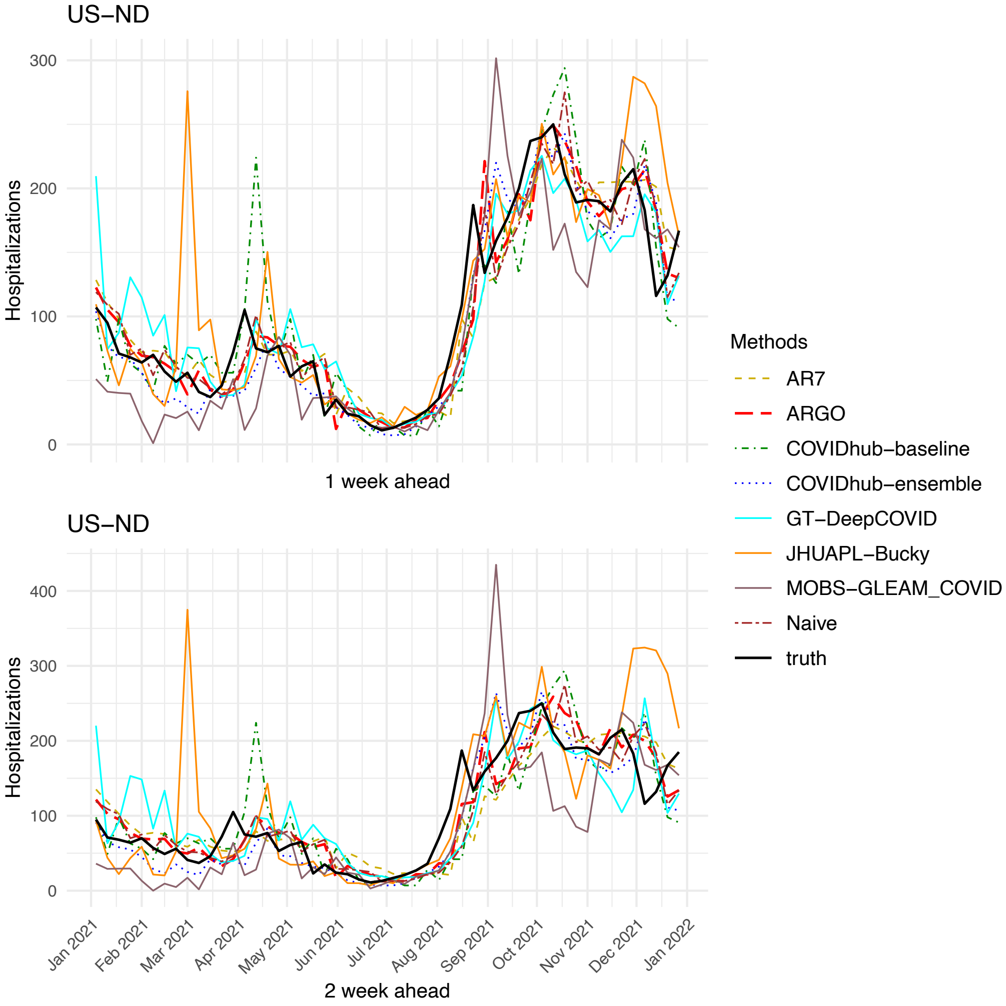

Figure S9. Plots of the COVID-19 hospitalizations 1 week (top), 2 weeks (bottom) ahead estimates of all compared models for New North Dakota (ND).

Methods	RMSE		MAE		Cor	
	1 Week Ahead	2 Week Ahead	1 Week Ahead	2 Week Ahead	1 Week Ahead	2 Week Ahead
ARGO	52.161	90.248	**26.781**	41.176	**0.915**	0.766
AR7	**43.867**	83.042	29.299	46.742	0.909	0.741
COVIDhub-ensemble	54.563	84.071	26.885	**41.038**	0.885	0.760
Naive	60.218	94.871	30.596	47.558	0.834	0.655
MOBS-GLEAM_COVID	53.551	84.700	32.923	45.077	0.870	0.737
COVIDhub-baseline	63.271	98.794	39.096	53.250	0.802	0.608
GT-DeepCOVID	78.457	107.707	38.295	57.312	0.679	0.506
JHUAPL-Bucky	53.965	**72.660**	33.434	50.459	0.863	**0.845**

Table S13. Comparison of different methods for state-level COVID-19 1 to 2 weeks ahead hospitalizations predictions in District of Columbia (DC). The MSE, MAE, and correlation are reported and best performed method is highlighted in boldface.

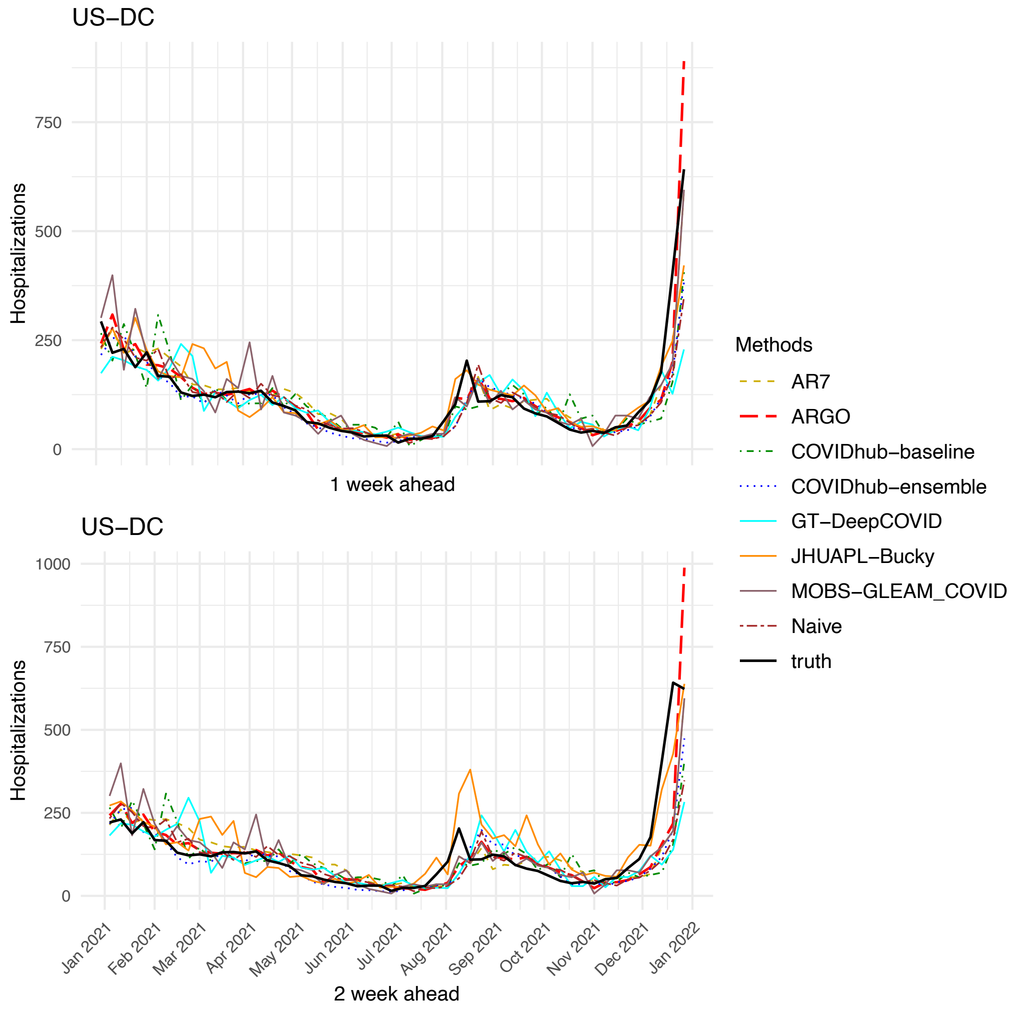

Figure S10. Plots of the COVID-19 hospitalizations 1 week (top), 2 weeks (bottom) ahead estimates of all compared models for New District of Columbia (DC).

Methods	RMSE		MAE		Cor	
	1 Week Ahead	2 Week Ahead	1 Week Ahead	2 Week Ahead	1 Week Ahead	2 Week Ahead
ARGO	**52.704**	86.347	**39.438**	61.805	**0.965**	0.898
AR7	59.496	101.480	42.110	67.705	0.960	0.870
COVIDhub-ensemble	55.654	**75.835**	41.750	**61.558**	0.960	**0.925**
Naive	61.445	98.872	43.788	70.981	0.951	0.867
MOBS-GLEAM_COVID	80.503	119.381	60.808	89.135	0.932	0.849
COVIDhub-baseline	86.486	119.014	65.481	83.942	0.904	0.811
GT-DeepCOVID	91.979	121.802	66.766	87.935	0.903	0.823
JHUAPL-Bucky	130.309	190.096	82.353	115.266	0.788	0.696

Table S14. Comparison of different methods for state-level COVID-19 1 to 2 weeks ahead hospitalizations predictions in New Mexico (NM). The MSE, MAE, and correlation are reported and best performed method is highlighted in boldface.

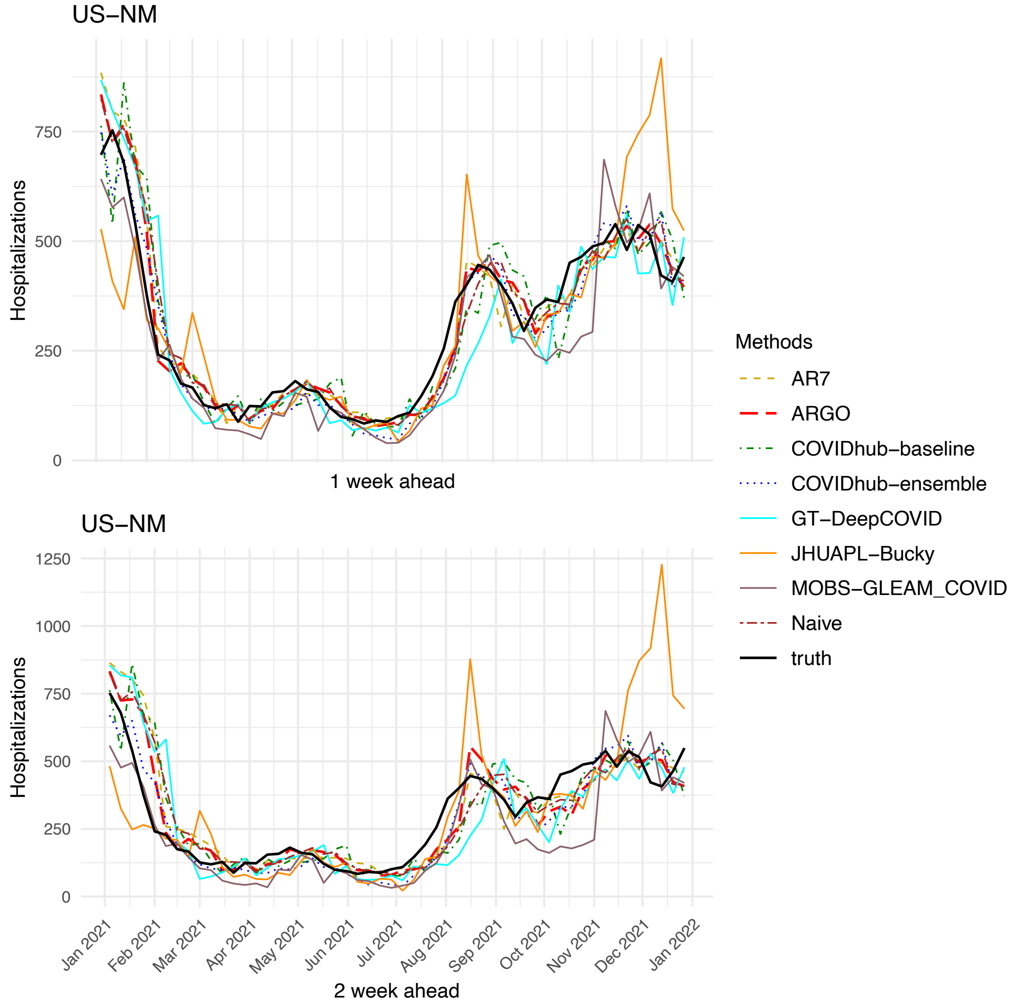

Figure S11. Plots of the COVID-19 hospitalizations 1 week (top), 2 weeks (bottom) ahead estimates of all compared models for New New Mexico (NM).

Methods	RMSE		MAE		Cor	
	1 Week Ahead	2 Week Ahead	1 Week Ahead	2 Week Ahead	1 Week Ahead	2 Week Ahead
ARGO	**292.893**	572.129	**173.725**	**345.365**	**0.958**	0.845
AR7	320.133	623.691	205.600	439.986	0.951	0.801
COVIDhub-ensemble	384.678	**559.940**	232.000	370.038	0.938	**0.882**
Naive	397.855	671.008	253.673	453.885	0.916	0.754
MOBS-GLEAM_COVID	503.154	602.235	311.804	397.714	0.903	0.867
COVIDhub-baseline	480.136	696.934	293.904	466.615	0.887	0.752
GT-DeepCOVID	425.103	771.140	289.004	518.862	0.935	0.819
JHUAPL-Bucky	881.825	889.419	618.246	659.238	0.731	0.678

Table S15. Comparison of different methods for state-level COVID-19 1 to 2 weeks ahead hospitalizations predictions in Kentucky (KY). The MSE, MAE, and correlation are reported and best performed method is highlighted in boldface.

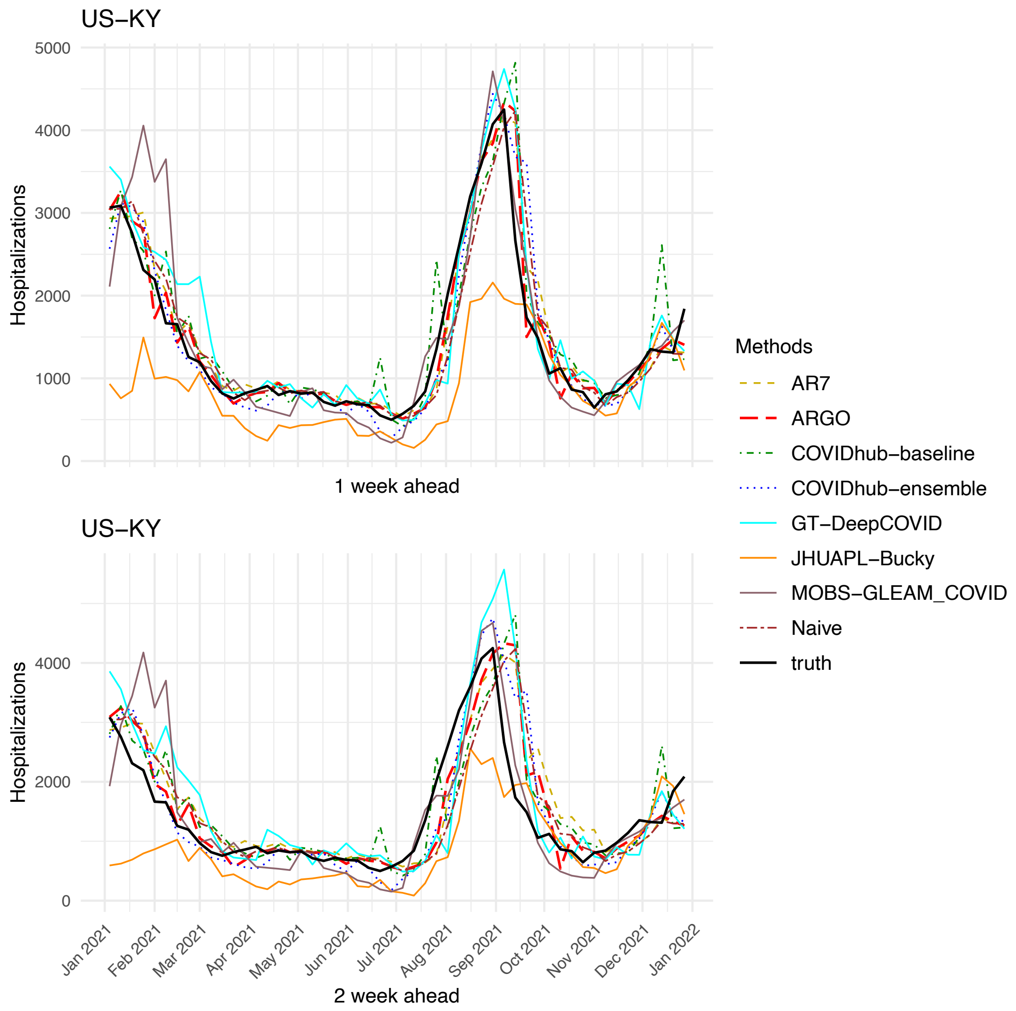

Figure S12. Plots of the COVID-19 hospitalizations 1 week (top), 2 weeks (bottom) ahead estimates of all compared models for New Kentucky (KY).

Methods	RMSE		MAE		Cor	
	1 Week Ahead	2 Week Ahead	1 Week Ahead	2 Week Ahead	1 Week Ahead	2 Week Ahead
ARGO	**26.297**	43.293	**16.800**	26.303	**0.923**	0.823
AR7	33.210	52.850	20.324	34.080	0.876	0.723
COVIDhub-ensemble	30.591	**32.607**	18.962	**22.385**	0.899	**0.905**
Naive	31.985	46.681	19.673	29.731	0.884	0.789
MOBS-GLEAM_COVID	30.318	44.141	21.517	29.541	0.915	0.832
COVIDhub-baseline	156.728	155.376	48.212	52.962	0.258	0.289
GT-DeepCOVID	43.020	56.805	29.981	40.554	0.802	0.712
JHUAPL-Bucky	57.462	89.356	43.875	59.223	0.913	0.865

Table S16. Comparison of different methods for state-level COVID-19 1 to 2 weeks ahead hospitalizations predictions in Rhode Island (RI). The MSE, MAE, and correlation are reported and best performed method is highlighted in boldface.

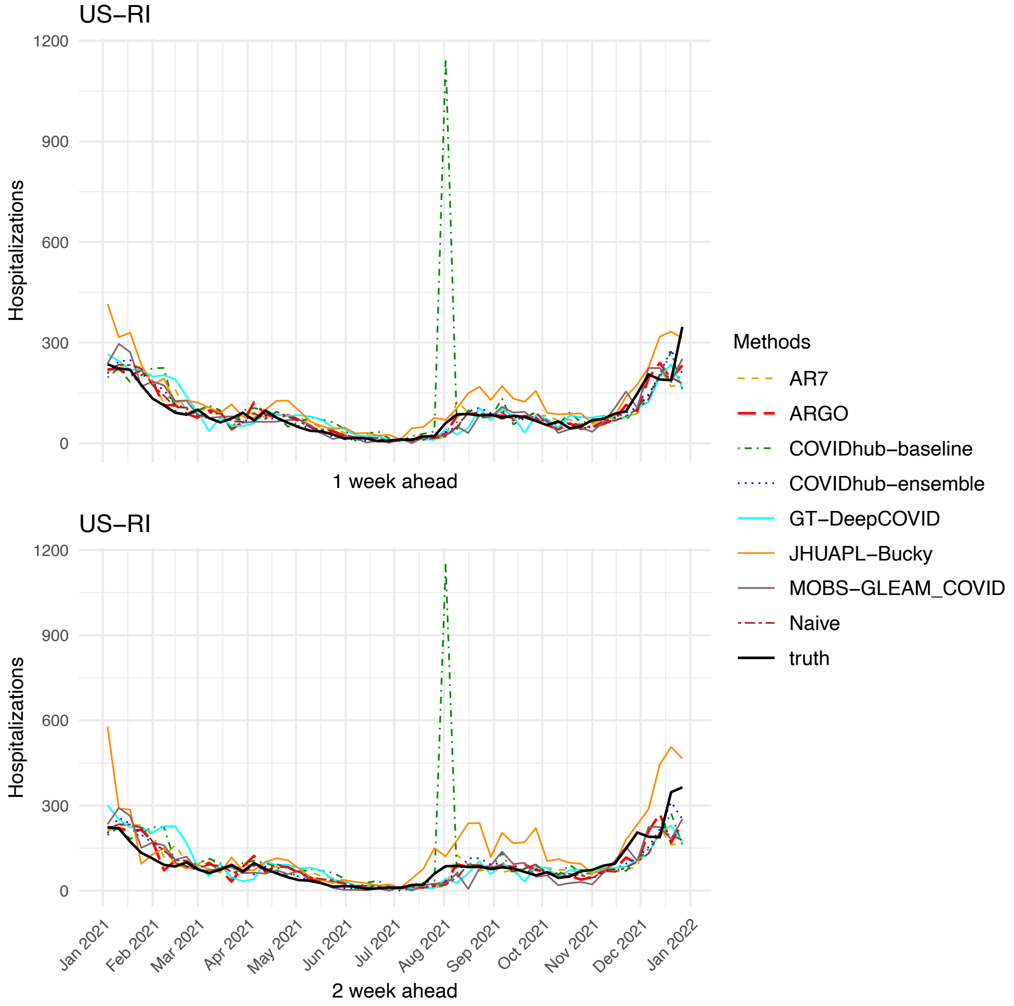

Figure S13. Plots of the COVID-19 hospitalizations 1 week (top), 2 weeks (bottom) ahead estimates of all compared models for New Rhode Island (RI).

Methods	RMSE		MAE		Cor	
	1 Week Ahead	2 Week Ahead	1 Week Ahead	2 Week Ahead	1 Week Ahead	2 Week Ahead
ARGO	**55.085**	**105.777**	**44.320**	**77.583**	**0.976**	**0.918**
AR7	72.486	135.293	56.607	100.931	0.959	0.859
COVIDhub-ensemble	83.651	127.510	63.231	96.231	0.954	0.898
Naive	77.737	138.919	60.135	104.558	0.953	0.853
MOBS-GLEAM_COVID	136.195	178.262	92.409	133.542	0.918	0.836
COVIDhub-baseline	99.115	146.553	80.865	112.135	0.921	0.832
GT-DeepCOVID	126.739	201.046	98.453	150.733	0.926	0.804
JHUAPL-Bucky	143.570	274.272	98.404	172.571	0.875	0.777

Table S17. Comparison of different methods for state-level COVID-19 1 to 2 weeks ahead hospitalizations predictions in Kansas (KS). The MSE, MAE, and correlation are reported and best performed method is highlighted in boldface.

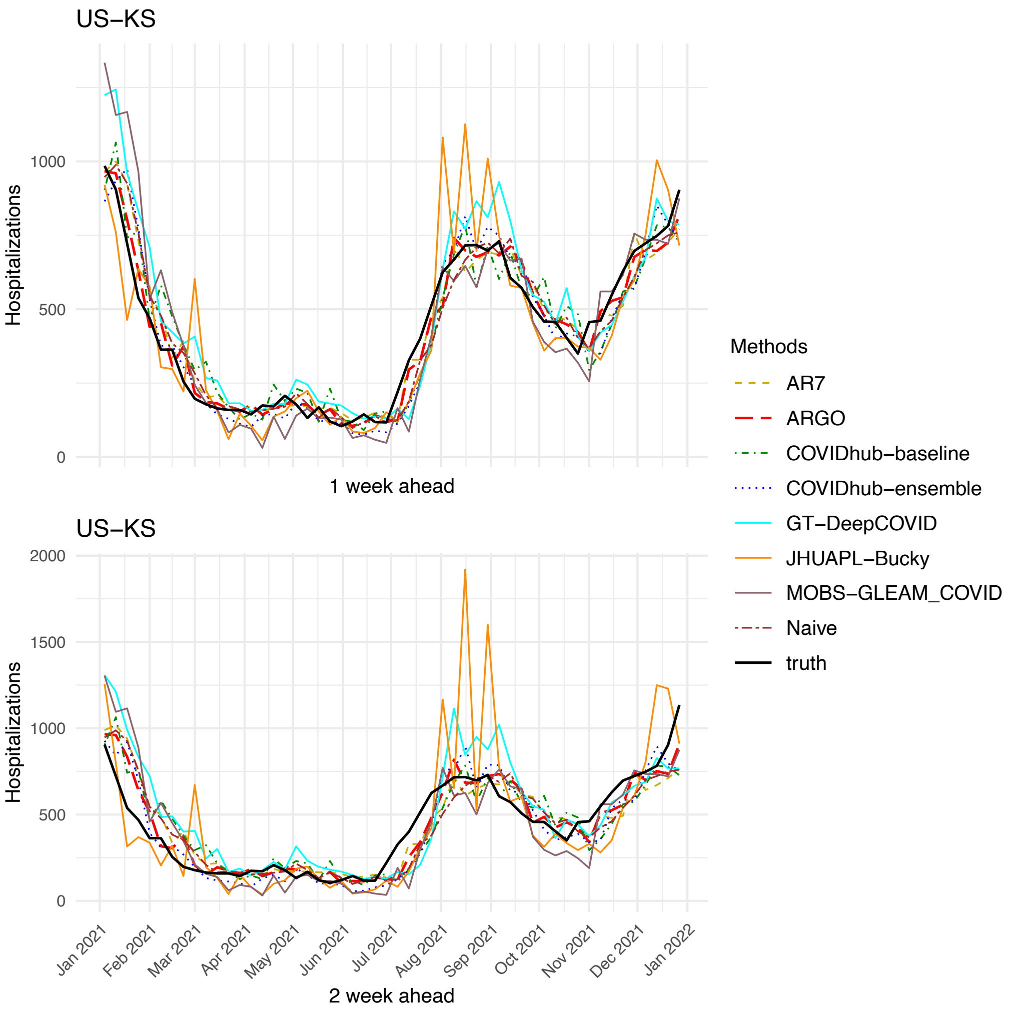

Figure S14. Plots of the COVID-19 hospitalizations 1 week (top), 2 weeks (bottom) ahead estimates of all compared models for New Kansas (KS).

Methods	RMSE		MAE		Cor	
	1 Week Ahead	2 Week Ahead	1 Week Ahead	2 Week Ahead	1 Week Ahead	2 Week Ahead
ARGO	29.409	40.961	**18.659**	29.301	0.919	0.855
AR7	27.686	40.538	21.209	32.168	0.927	0.848
COVIDhub-ensemble	33.475	41.036	23.115	30.019	0.908	0.876
Naive	**26.823**	**36.228**	18.962	**28.115**	**0.932**	**0.881**
MOBS-GLEAM_COVID	72.103	89.302	43.313	51.992	0.701	0.577
COVIDhub-baseline	171.870	172.841	56.981	63.788	0.235	0.224
GT-DeepCOVID	36.435	52.665	27.316	38.952	0.870	0.736
JHUAPL-Bucky	69.894	116.933	43.682	66.638	0.812	0.765

Table S18. Comparison of different methods for state-level COVID-19 1 to 2 weeks ahead hospitalizations predictions in Maine (ME). The MSE, MAE, and correlation are reported and best performed method is highlighted in boldface.

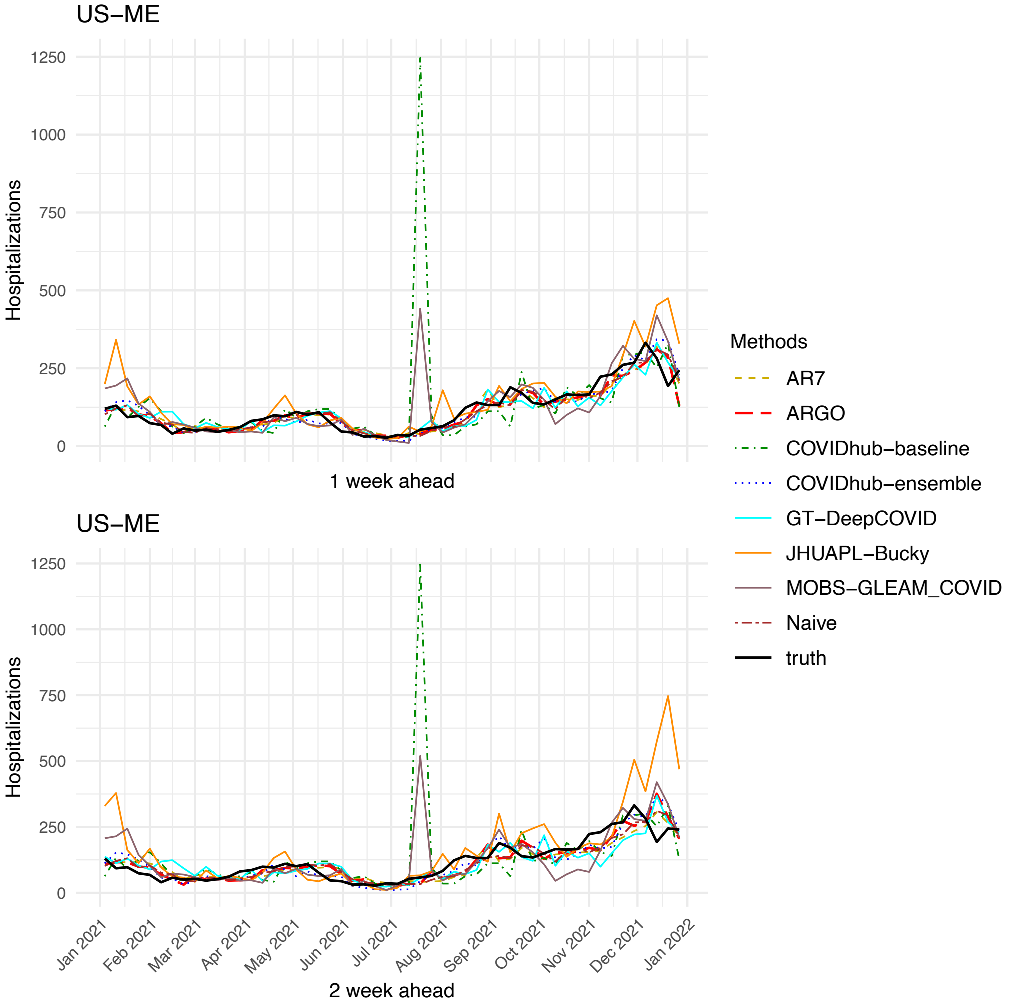

Figure S15. Plots of the COVID-19 hospitalizations 1 week (top), 2 weeks (bottom) ahead estimates of all compared models for New Maine (ME).

Methods	RMSE		MAE		Cor	
	1 Week Ahead	2 Week Ahead	1 Week Ahead	2 Week Ahead	1 Week Ahead	2 Week Ahead
ARGO	**41.129**	**86.389**	**33.519**	**65.204**	**0.984**	**0.934**
AR7	55.254	101.240	43.556	76.814	0.970	0.908
COVIDhub-ensemble	60.696	102.870	50.000	79.885	0.972	0.926
Naive	54.954	101.728	44.154	79.442	0.969	0.910
MOBS-GLEAM_COVID	78.540	114.548	63.017	87.265	0.957	0.908
COVIDhub-baseline	73.244	109.439	58.154	82.981	0.948	0.893
GT-DeepCOVID	106.453	181.291	85.626	132.804	0.904	0.771
JHUAPL-Bucky	183.416	200.434	89.515	141.903	0.748	0.810

Table S19. Comparison of different methods for state-level COVID-19 1 to 2 weeks ahead hospitalizations predictions in Iowa (IA). The MSE, MAE, and correlation are reported and best performed method is highlighted in boldface.

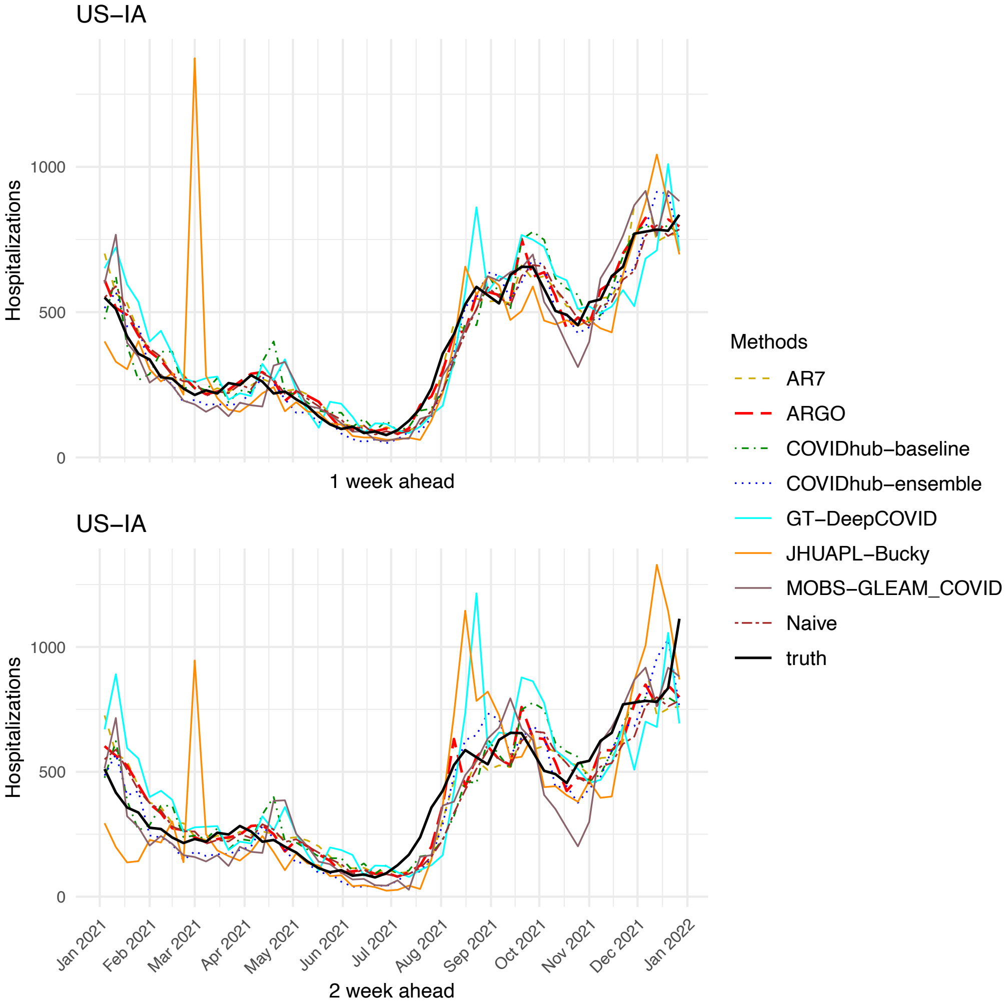

Figure S16. Plots of the COVID-19 hospitalizations 1 week (top), 2 weeks (bottom) ahead estimates of all compared models for New Iowa (IA).

Methods	RMSE		MAE		Cor	
	1 Week Ahead	2 Week Ahead	1 Week Ahead	2 Week Ahead	1 Week Ahead	2 Week Ahead
ARGO	**24.851**	48.109	**19.009**	34.219	**0.957**	0.850
AR7	29.183	46.975	21.934	35.373	0.935	0.840
COVIDhub-ensemble	36.679	55.077	25.288	35.712	0.916	0.843
Naive	29.893	**43.140**	22.962	**30.808**	0.933	**0.869**
MOBS-GLEAM_COVID	56.555	84.251	39.035	53.683	0.858	0.730
COVIDhub-baseline	37.925	48.689	26.923	35.846	0.909	0.849
GT-DeepCOVID	35.867	55.116	27.970	42.391	0.902	0.787
JHUAPL-Bucky	40.575	70.234	28.860	45.971	0.885	0.777

Table S20. Comparison of different methods for state-level COVID-19 1 to 2 weeks ahead hospitalizations predictions in South Dakota (SD). The MSE, MAE, and correlation are reported and best performed method is highlighted in boldface.

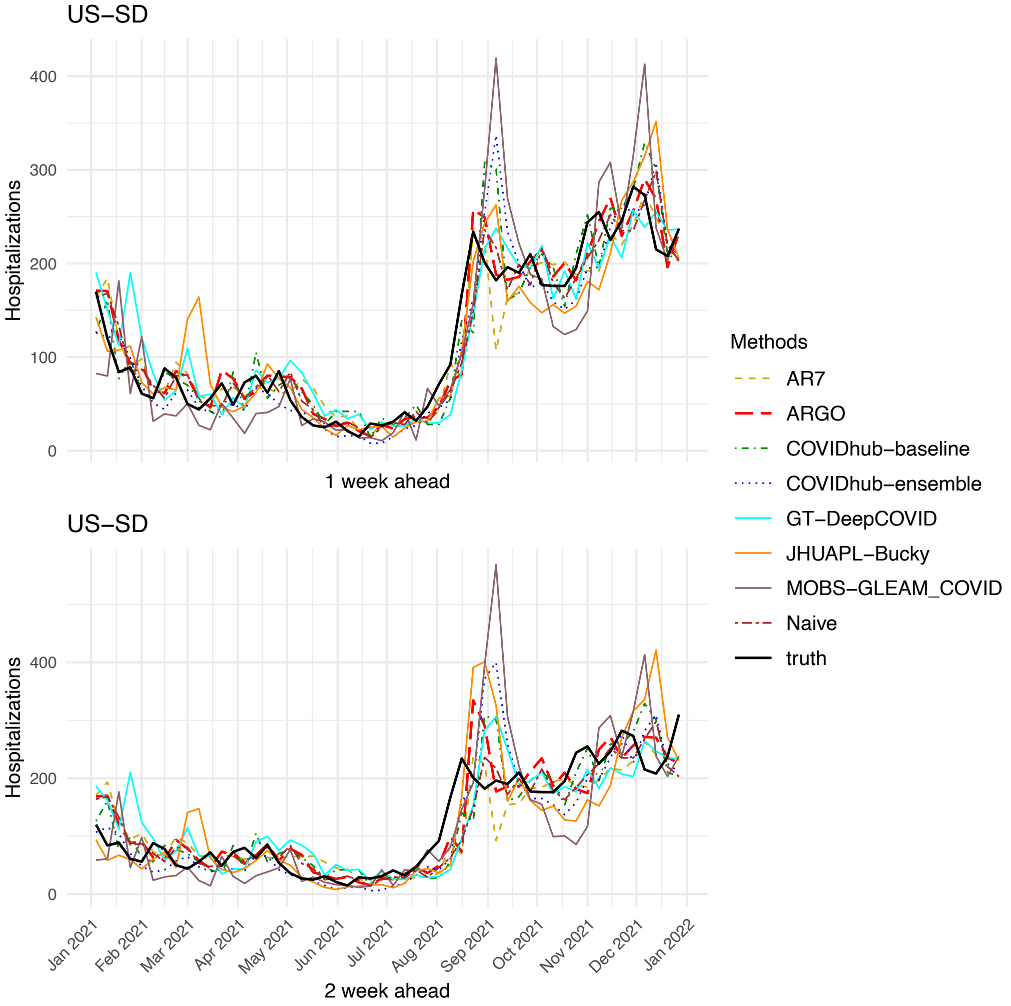

Figure S17. Plots of the COVID-19 hospitalizations 1 week (top), 2 weeks (bottom) ahead estimates of all compared models for New South Dakota (SD).

Methods	RMSE		MAE		Cor	
	1 Week Ahead	2 Week Ahead	1 Week Ahead	2 Week Ahead	1 Week Ahead	2 Week Ahead
ARGO	**155.610**	**327.579**	**112.695**	**241.830**	**0.981**	**0.920**
AR7	181.258	351.439	137.329	267.276	0.972	0.879
COVIDhub-ensemble	220.531	365.955	155.288	256.962	0.967	0.909
Naive	221.351	400.950	161.058	296.923	0.955	0.845
MOBS-GLEAM_COVID	391.987	513.663	235.409	348.162	0.924	0.869
COVIDhub-baseline	225.083	397.098	172.481	303.385	0.952	0.844
GT-DeepCOVID	315.873	530.474	230.539	382.922	0.947	0.852
JHUAPL-Bucky	461.762	484.641	316.105	338.143	0.876	0.808

Table S21. Comparison of different methods for state-level COVID-19 1 to 2 weeks ahead hospitalizations predictions in Oklahoma (OK). The MSE, MAE, and correlation are reported and best performed method is highlighted in boldface.

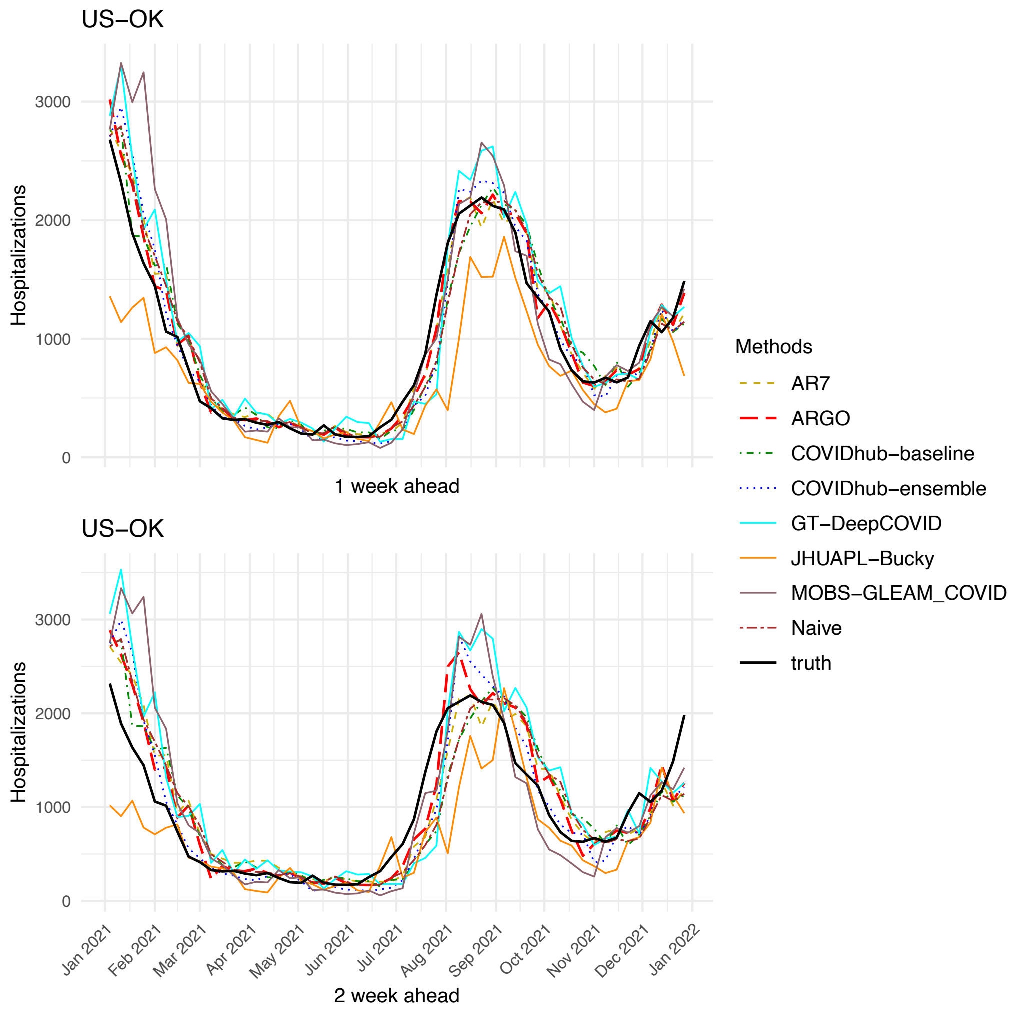

Figure S18. Plots of the COVID-19 hospitalizations 1 week (top), 2 weeks (bottom) ahead estimates of all compared models for New Oklahoma (OK).

Methods	RMSE		MAE		Cor	
	1 Week Ahead	2 Week Ahead	1 Week Ahead	2 Week Ahead	1 Week Ahead	2 Week Ahead
ARGO	**405.668**	**837.908**	291.744	**572.543**	**0.971**	**0.878**
AR7	421.467	917.717	**288.420**	646.876	0.965	0.830
COVIDhub-ensemble	667.843	1073.718	445.942	688.173	0.942	0.860
Naive	635.860	1145.531	431.481	789.596	0.920	0.741
MOBS-GLEAM_COVID	806.039	1428.043	498.694	882.558	0.939	0.844
COVIDhub-baseline	950.898	1298.161	778.288	1073.135	0.892	0.750
GT-DeepCOVID	712.933	1132.578	528.956	849.849	0.914	0.798
JHUAPL-Bucky	977.750	1247.234	654.741	925.129	0.822	0.732

Table S22. Comparison of different methods for state-level COVID-19 1 to 2 weeks ahead hospitalizations predictions in Georgia (GA). The MSE, MAE, and correlation are reported and best performed method is highlighted in boldface.

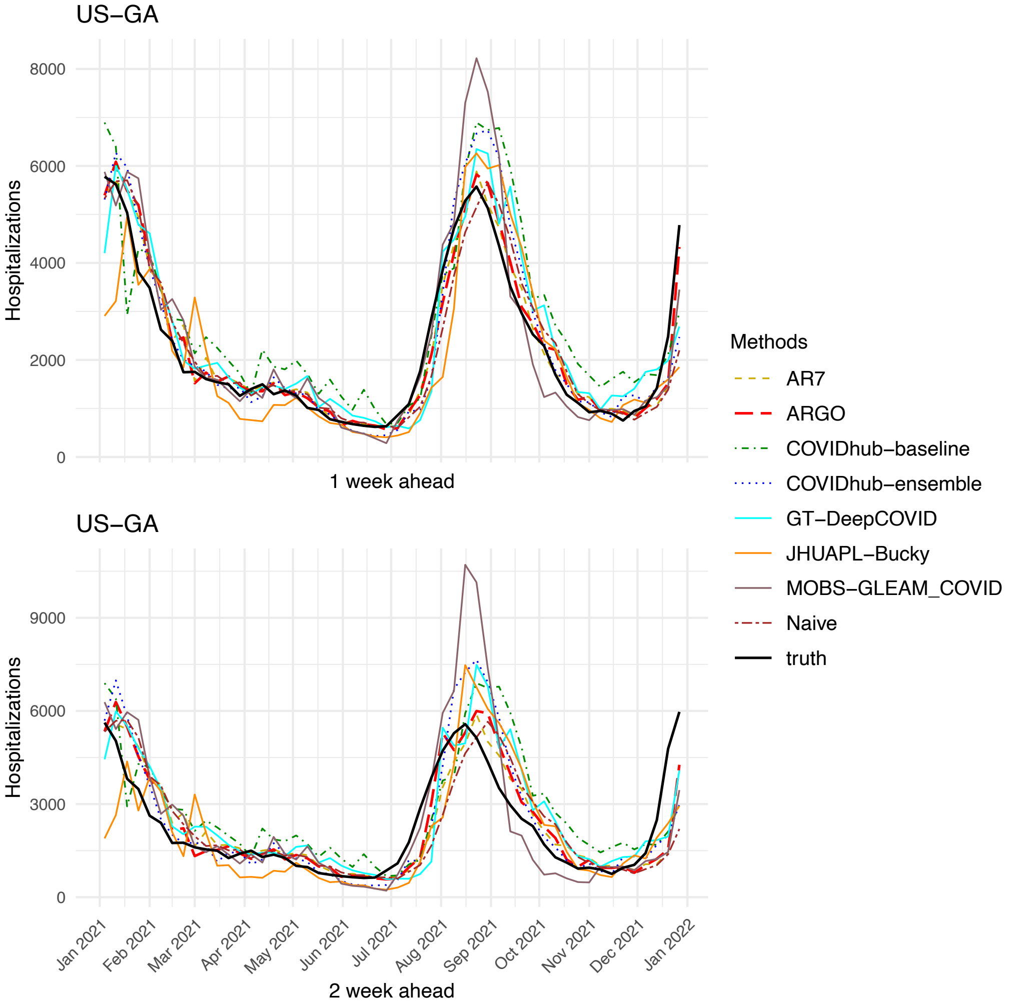

Figure S19. Plots of the COVID-19 hospitalizations 1 week (top), 2 weeks (bottom) ahead estimates of all compared models for New Georgia (GA).

Methods	RMSE		MAE		Cor	
	1 Week Ahead	2 Week Ahead	1 Week Ahead	2 Week Ahead	1 Week Ahead	2 Week Ahead
ARGO	**32.251**	**65.927**	**25.713**	44.635	**0.952**	0.857
AR7	44.365	84.970	32.412	55.567	0.907	0.743
COVIDhub-ensemble	37.879	66.684	26.269	**43.442**	0.937	0.860
Naive	47.366	84.928	31.577	54.404	0.893	0.745
MOBS-GLEAM_COVID	44.520	87.684	32.072	59.179	0.918	0.754
COVIDhub-baseline	56.645	89.753	43.269	64.058	0.852	0.713
GT-DeepCOVID	46.662	84.060	34.262	63.956	0.904	0.761
JHUAPL-Bucky	44.880	78.692	33.632	52.272	0.928	**0.877**

Table S23. Comparison of different methods for state-level COVID-19 1 to 2 weeks ahead hospitalizations predictions in Delaware (DE). The MSE, MAE, and correlation are reported and best performed method is highlighted in boldface.

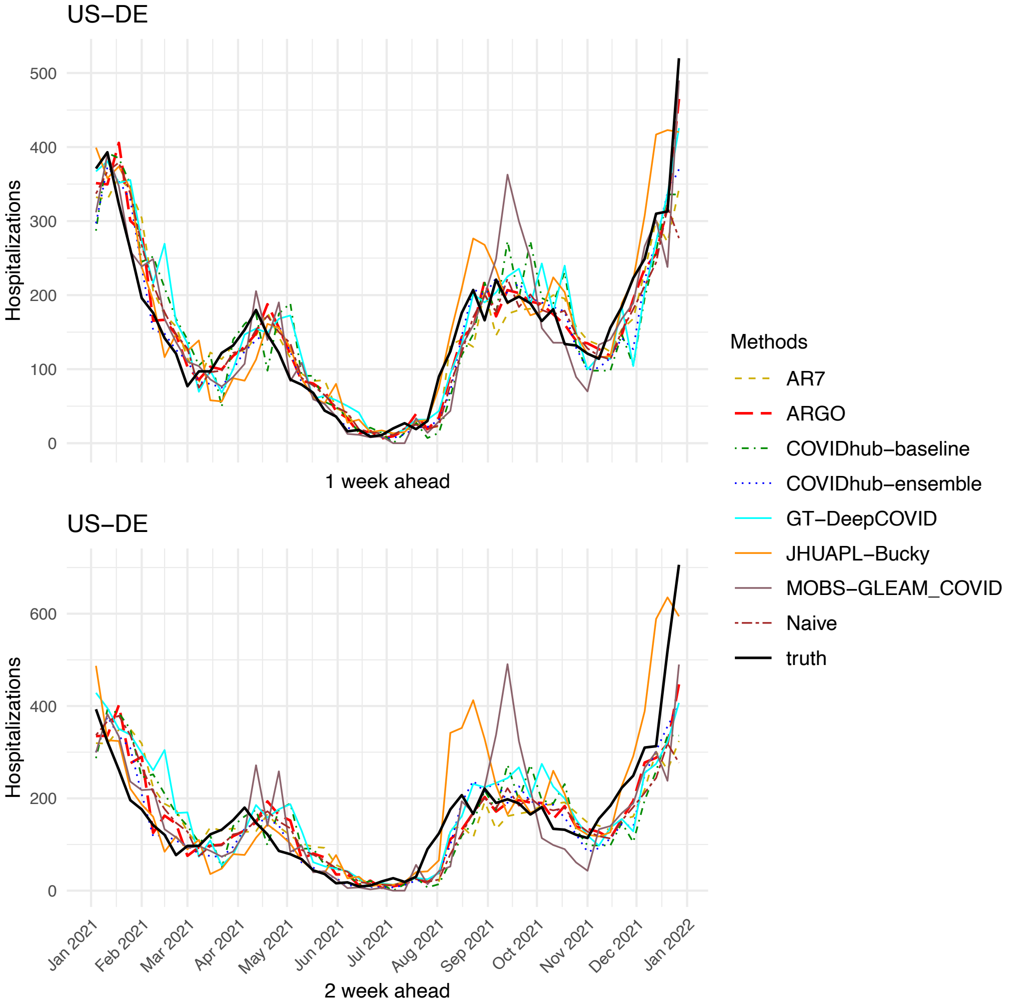

Figure S20. Plots of the COVID-19 hospitalizations 1 week (top), 2 weeks (bottom) ahead estimates of all compared models for New Delaware (DE).

Methods	RMSE		MAE		Cor	
	1 Week Ahead	2 Week Ahead	1 Week Ahead	2 Week Ahead	1 Week Ahead	2 Week Ahead
ARGO	138.307	277.839	**96.234**	181.331	**0.951**	0.826
AR7	**136.376**	**237.028**	98.253	167.551	0.948	0.866
COVIDhub-ensemble	150.712	245.002	100.058	**159.212**	0.943	**0.874**
Naive	156.975	270.490	108.077	188.096	0.930	0.827
MOBS-GLEAM_COVID	237.516	372.914	174.290	265.896	0.878	0.735
COVIDhub-baseline	175.407	279.481	121.385	173.750	0.912	0.814
GT-DeepCOVID	173.893	297.692	129.830	222.592	0.916	0.791
JHUAPL-Bucky	254.007	350.913	183.530	270.130	0.842	0.776

Table S24. Comparison of different methods for state-level COVID-19 1 to 2 weeks ahead hospitalizations predictions in Colorado (CO). The MSE, MAE, and correlation are reported and best performed method is highlighted in boldface.

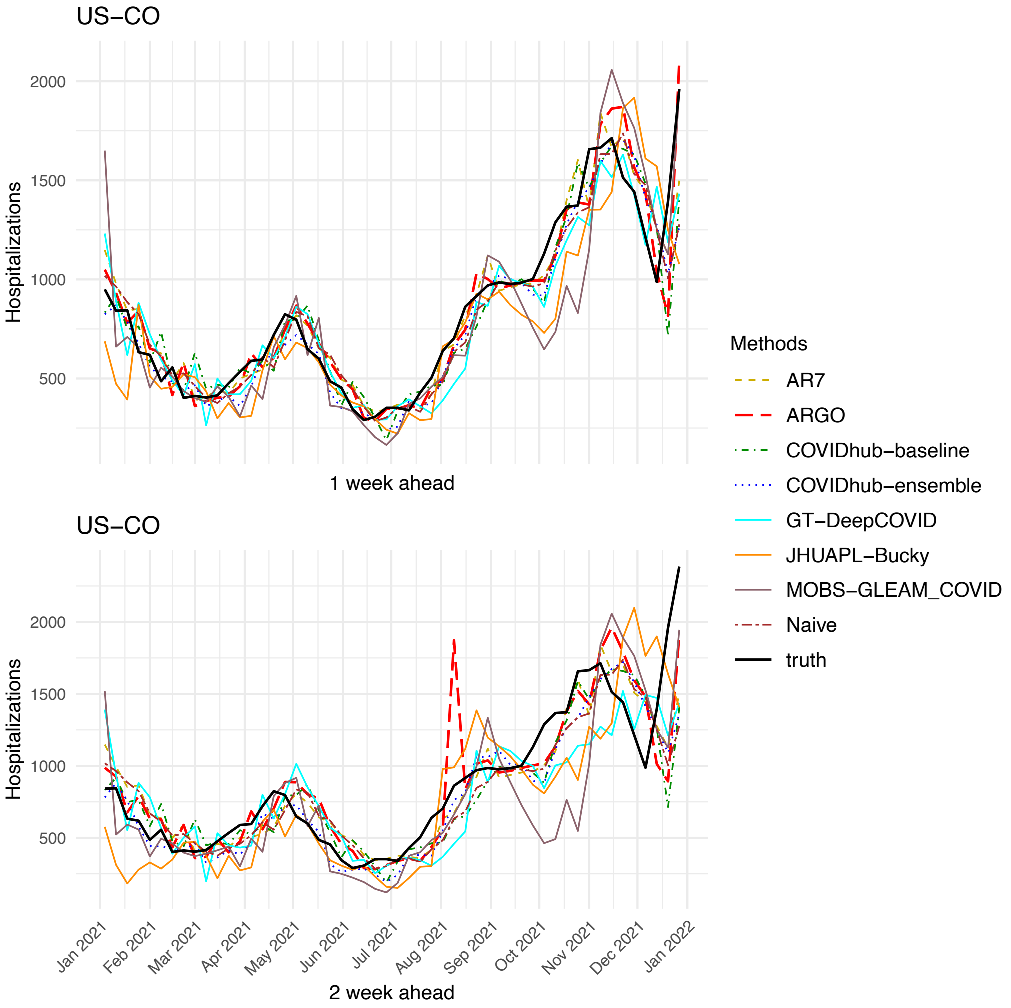

Figure S21. Plots of the COVID-19 hospitalizations 1 week (top), 2 weeks (bottom) ahead estimates of all compared models for New Colorado (CO).

Methods	RMSE		MAE		Cor	
	1 Week Ahead	2 Week Ahead	1 Week Ahead	2 Week Ahead	1 Week Ahead	2 Week Ahead
ARGO	**47.255**	83.070	**34.050**	61.010	**0.976**	0.927
AR7	51.519	95.348	38.094	74.136	0.972	0.896
COVIDhub-ensemble	58.557	**75.546**	42.577	**56.308**	0.965	**0.942**
Naive	60.159	96.335	45.096	76.173	0.957	0.890
MOBS-GLEAM_COVID	78.844	124.249	50.910	84.301	0.932	0.820
COVIDhub-baseline	75.745	107.546	54.673	80.058	0.936	0.871
GT-DeepCOVID	71.182	92.594	53.644	70.154	0.939	0.897
JHUAPL-Bucky	134.789	133.093	91.570	100.631	0.891	0.909

Table S25. Comparison of different methods for state-level COVID-19 1 to 2 weeks ahead hospitalizations predictions in Montana (MT). The MSE, MAE, and correlation are reported and best performed method is highlighted in boldface.

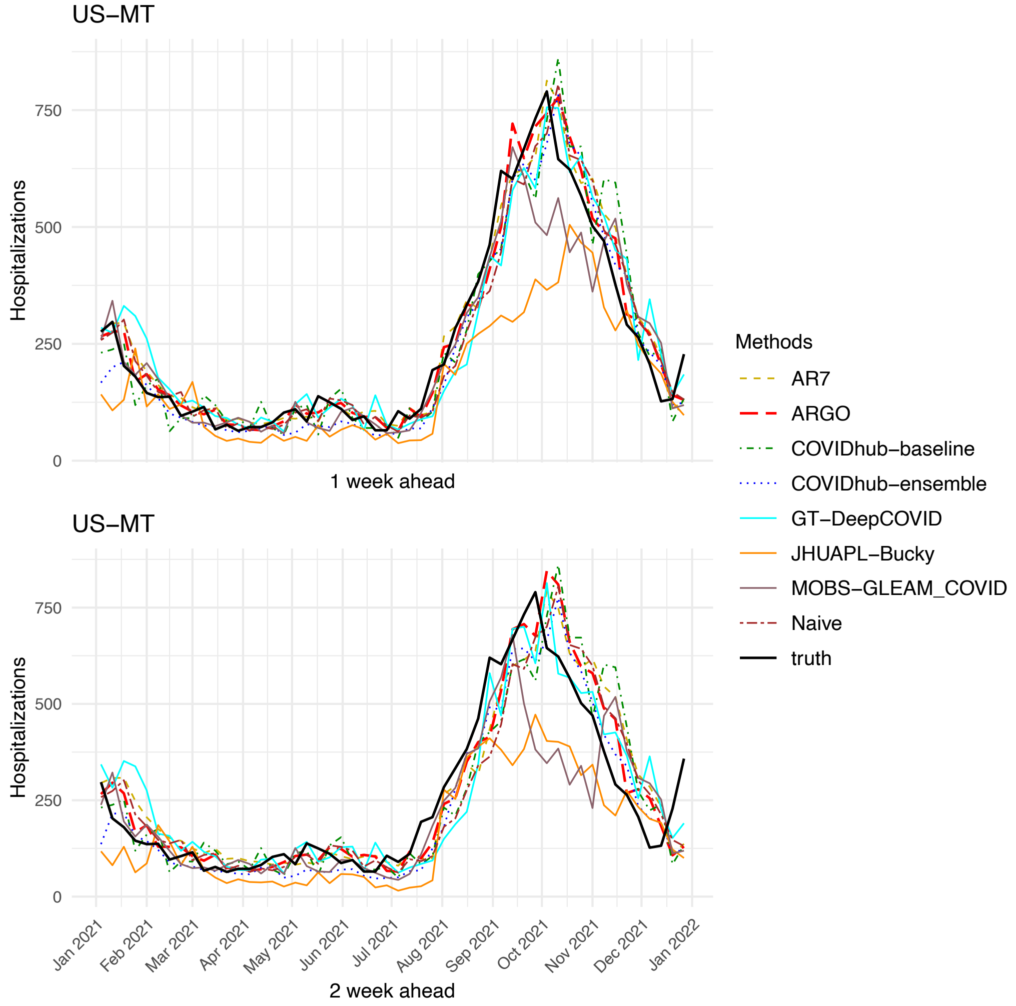

Figure S22. Plots of the COVID-19 hospitalizations 1 week (top), 2 weeks (bottom) ahead estimates of all compared models for New Montana (MT).

Methods	RMSE		MAE		Cor	
	1 Week Ahead	2 Week Ahead	1 Week Ahead	2 Week Ahead	1 Week Ahead	2 Week Ahead
ARGO	**86.307**	**165.545**	**62.981**	**106.442**	**0.950**	**0.822**
AR7	99.743	196.085	72.532	126.137	0.930	0.741
COVIDhub-ensemble	112.266	195.618	73.942	121.769	0.927	0.788
Naive	98.141	191.360	71.827	126.385	0.933	0.759
MOBS-GLEAM_COVID	126.663	216.742	101.806	163.029	0.927	0.788
COVIDhub-baseline	147.090	223.786	99.846	151.981	0.857	0.682
GT-DeepCOVID	130.237	225.229	96.337	165.418	0.920	0.748
JHUAPL-Bucky	262.864	381.213	178.968	251.930	0.894	0.762

Table S26. Comparison of different methods for state-level COVID-19 1 to 2 weeks ahead hospitalizations predictions in Nevada (NV). The MSE, MAE, and correlation are reported and best performed method is highlighted in boldface.

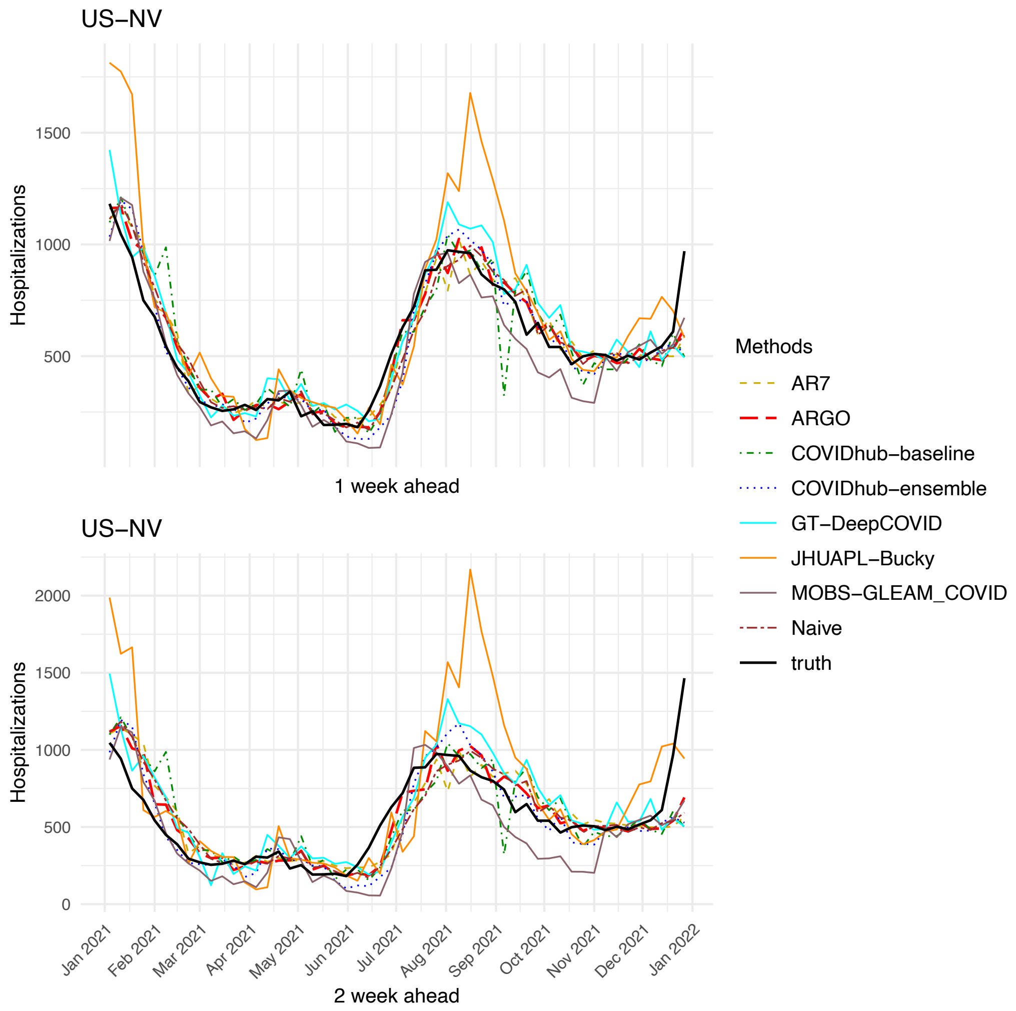

Figure S23. Plots of the COVID-19 hospitalizations 1 week (top), 2 weeks (bottom) ahead estimates of all compared models for New Nevada (NV).

Methods	RMSE		MAE		Cor	
	1 Week Ahead	2 Week Ahead	1 Week Ahead	2 Week Ahead	1 Week Ahead	2 Week Ahead
ARGO	325.881	499.206	183.123	355.240	0.930	0.841
AR7	**218.309**	**456.009**	**163.815**	**337.945**	**0.966**	0.843
COVIDhub-ensemble	302.950	512.788	220.942	356.865	0.937	0.845
Naive	299.536	558.426	219.904	401.288	0.930	0.762
MOBS-GLEAM_COVID	307.819	481.155	229.158	350.777	0.936	**0.860**
COVIDhub-baseline	334.135	575.388	243.308	413.692	0.909	0.739
GT-DeepCOVID	351.024	621.756	269.025	465.009	0.913	0.784
JHUAPL-Bucky	535.927	822.458	379.204	566.136	0.892	0.827

Table S27. Comparison of different methods for state-level COVID-19 1 to 2 weeks ahead hospitalizations predictions in North Carolina (NC). The MSE, MAE, and correlation are reported and best performed method is highlighted in boldface.

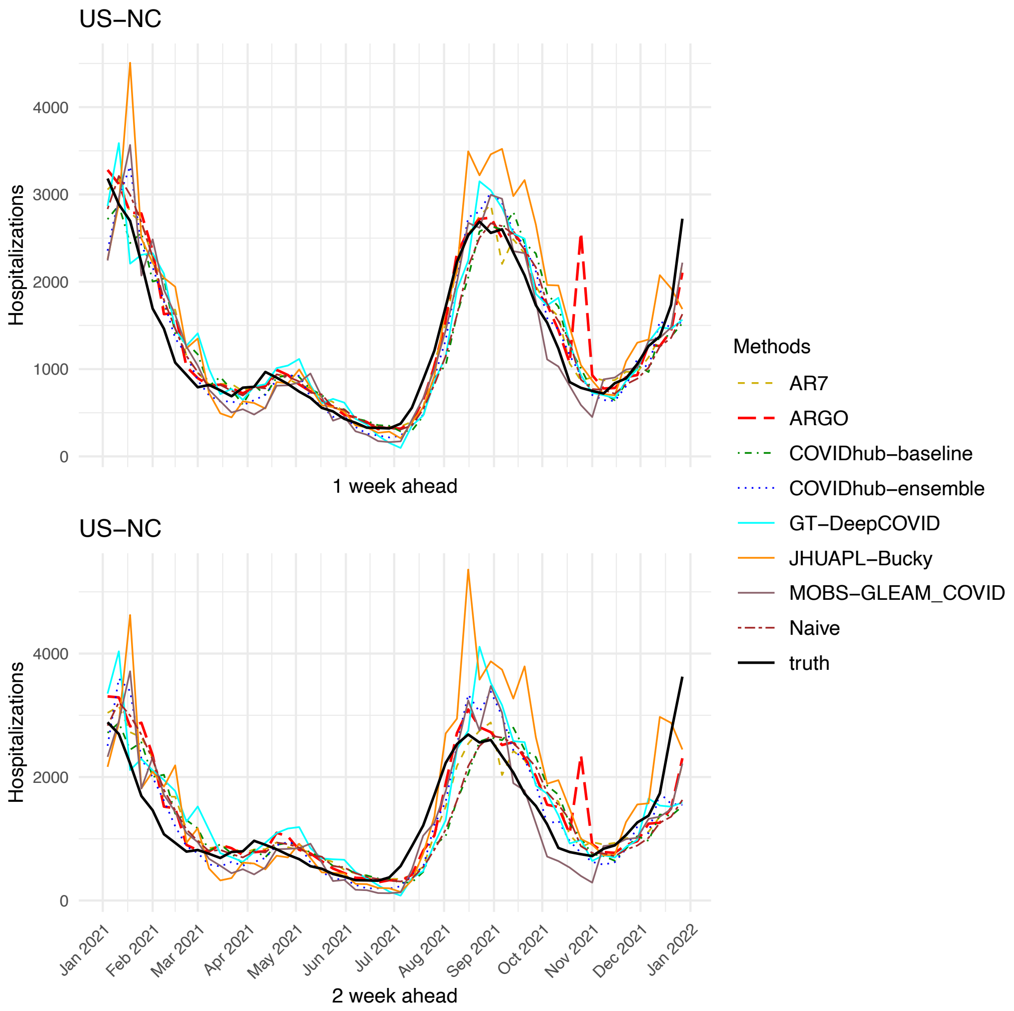

Figure S24. Plots of the COVID-19 hospitalizations 1 week (top), 2 weeks (bottom) ahead estimates of all compared models for New North Carolina (NC).

Methods	RMSE		MAE		Cor	
	1 Week Ahead	2 Week Ahead	1 Week Ahead	2 Week Ahead	1 Week Ahead	2 Week Ahead
ARGO	**42.379**	**72.701**	**32.991**	**53.479**	**0.965**	0.899
AR7	50.401	85.358	36.492	62.053	0.952	0.862
COVIDhub-ensemble	53.282	77.730	43.288	60.173	0.954	**0.909**
Naive	48.898	78.454	38.365	56.577	0.953	0.881
MOBS-GLEAM_COVID	112.130	155.510	81.188	111.434	0.795	0.641
COVIDhub-baseline	75.107	92.617	57.365	67.077	0.892	0.838
GT-DeepCOVID	64.564	79.838	48.014	62.257	0.920	0.887
JHUAPL-Bucky	107.763	154.243	80.663	123.174	0.839	0.762

Table S28. Comparison of different methods for state-level COVID-19 1 to 2 weeks ahead hospitalizations predictions in Utah (UT). The MSE, MAE, and correlation are reported and best performed method is highlighted in boldface.

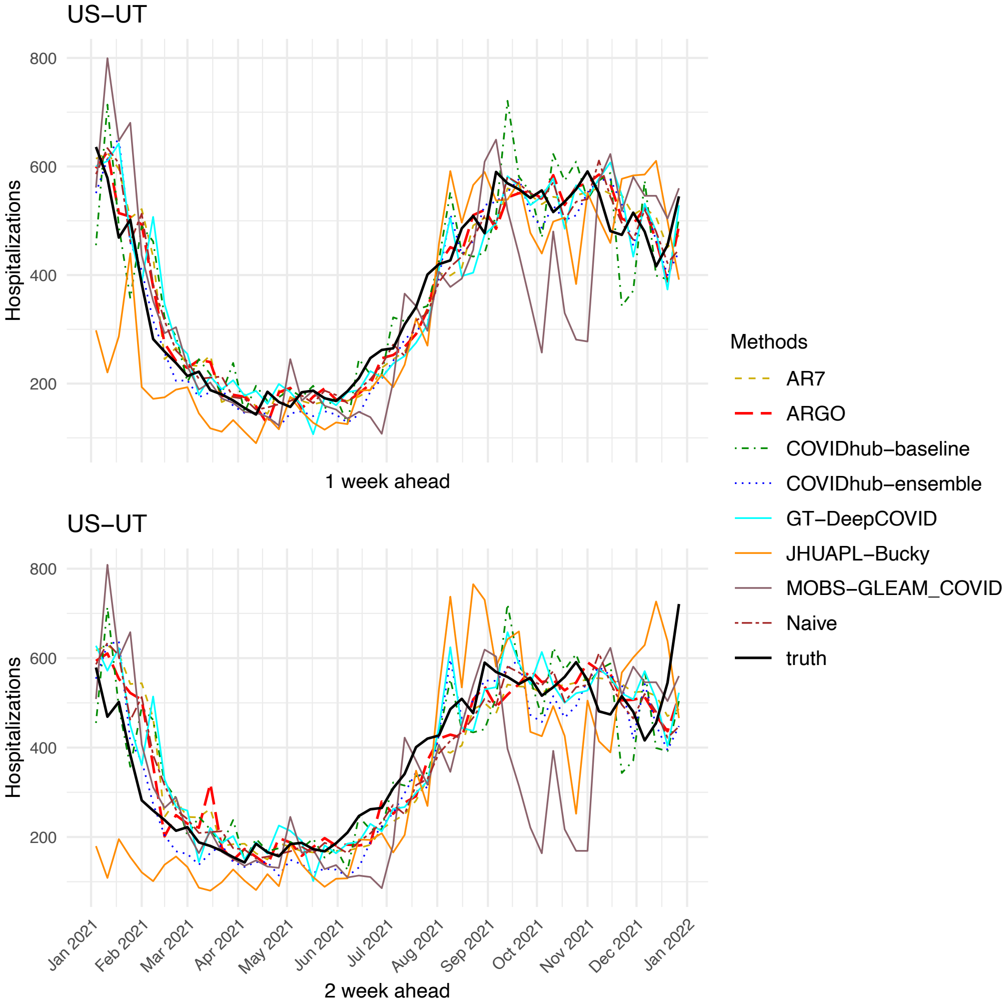

Figure S25. Plots of the COVID-19 hospitalizations 1 week (top), 2 weeks (bottom) ahead estimates of all compared models for New Utah (UT).

Methods	RMSE		MAE		Cor	
	1 Week Ahead	2 Week Ahead	1 Week Ahead	2 Week Ahead	1 Week Ahead	2 Week Ahead
ARGO	**42.130**	63.930	**30.963**	47.644	**0.954**	0.901
AR7	44.237	66.137	35.017	51.867	0.947	0.883
COVIDhub-ensemble	44.898	**63.320**	33.769	**44.596**	0.952	**0.910**
Naive	43.943	65.188	34.423	50.692	0.948	0.889
MOBS-GLEAM_COVID	60.825	91.525	42.365	65.689	0.922	0.822
COVIDhub-baseline	106.052	120.721	50.942	68.058	0.826	0.758
GT-DeepCOVID	62.474	87.314	46.571	66.467	0.897	0.819
JHUAPL-Bucky	100.201	192.865	65.111	112.632	0.861	0.711

Table S29. Comparison of different methods for state-level COVID-19 1 to 2 weeks ahead hospitalizations predictions in Nebraska (NE). The MSE, MAE, and correlation are reported and best performed method is highlighted in boldface.

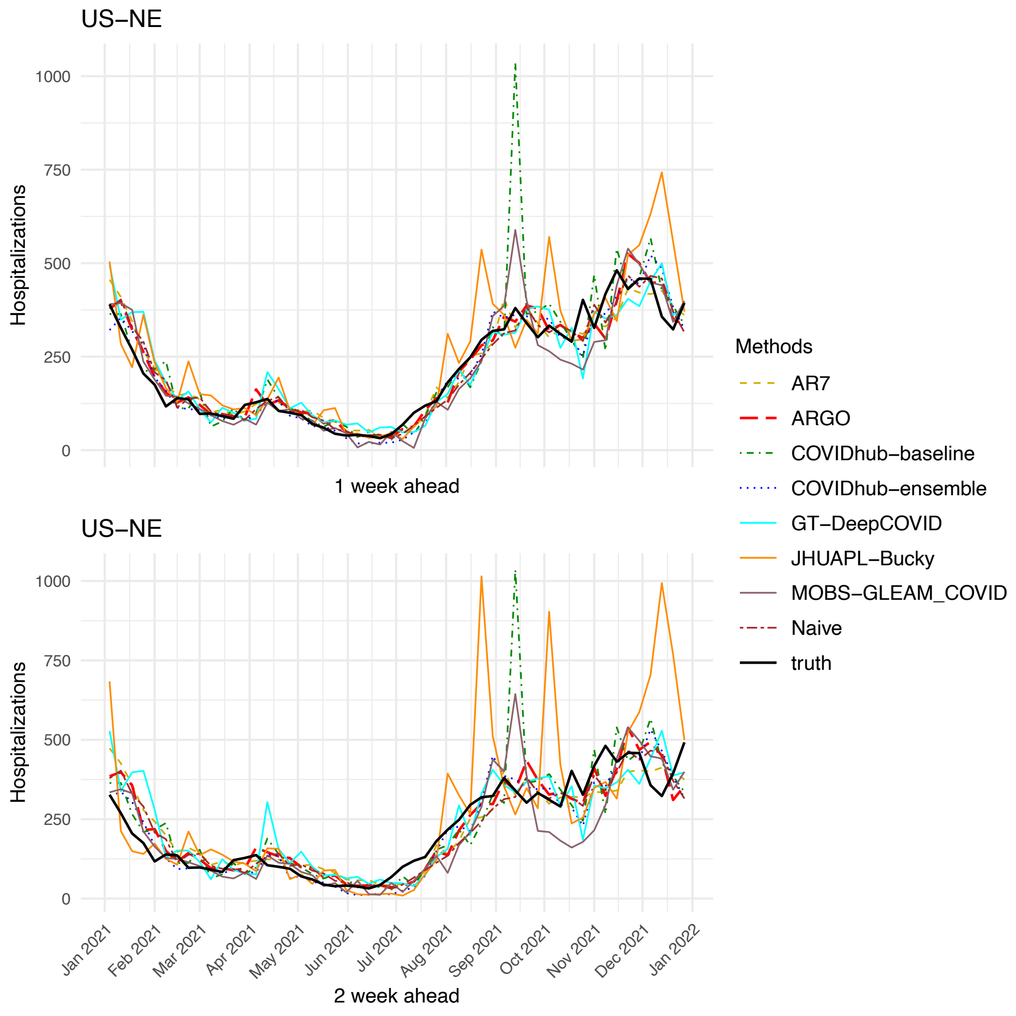

Figure S26. Plots of the COVID-19 hospitalizations 1 week (top), 2 weeks (bottom) ahead estimates of all compared models for New Nebraska (NE).

Methods	RMSE		MAE		Cor	
	1 Week Ahead	2 Week Ahead	1 Week Ahead	2 Week Ahead	1 Week Ahead	2 Week Ahead
ARGO	**480.564**	1253.041	**294.576**	**653.258**	**0.975**	0.850
AR7	484.782	**1176.086**	304.829	659.807	0.974	**0.869**
COVIDhub-ensemble	832.408	1338.327	396.269	702.096	0.922	0.833
Naive	876.339	1519.967	431.654	809.596	0.911	0.772
MOBS-GLEAM_COVID	688.959	1351.754	486.538	865.310	0.949	0.831
COVIDhub-baseline	971.889	1572.613	507.250	830.692	0.891	0.754
GT-DeepCOVID	660.281	1269.201	456.087	713.004	0.951	0.848
JHUAPL-Bucky	1127.248	1564.264	673.512	941.596	0.903	**0.869**

Table S30. Comparison of different methods for state-level COVID-19 1 to 2 weeks ahead hospitalizations predictions in New York (NY). The MSE, MAE, and correlation are reported and best performed method is highlighted in boldface.

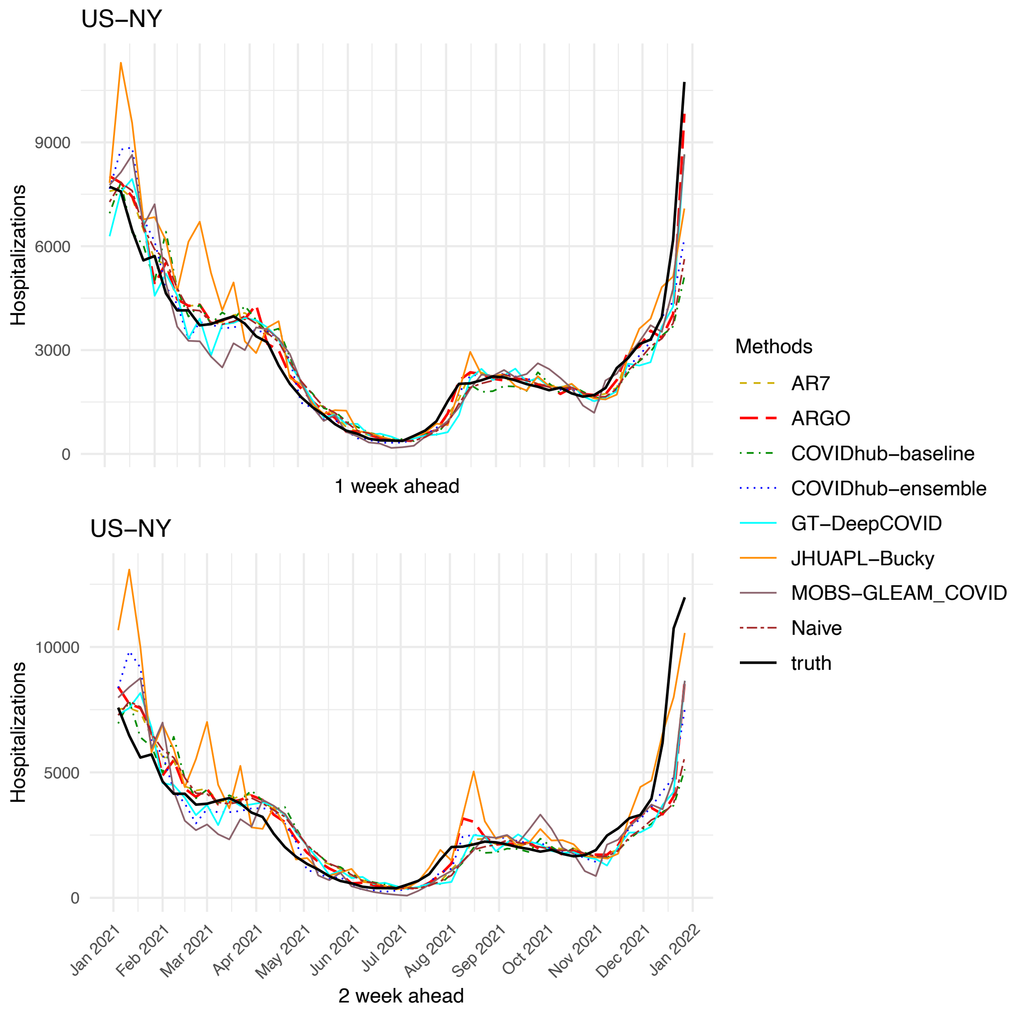

Figure S27. Plots of the COVID-19 hospitalizations 1 week (top), 2 weeks (bottom) ahead estimates of all compared models for New New York (NY).

Methods	RMSE		MAE		Cor	
	1 Week Ahead	2 Week Ahead	1 Week Ahead	2 Week Ahead	1 Week Ahead	2 Week Ahead
ARGO	70.956	114.018	49.118	82.550	0.945	0.859
AR7	**68.576**	121.906	49.623	89.501	0.945	0.823
COVIDhub-ensemble	68.721	**110.588**	**49.096**	**77.308**	**0.955**	0.892
Naive	78.339	136.946	57.019	95.731	0.925	0.774
MOBS-GLEAM_COVID	83.624	120.248	63.678	91.975	0.920	0.845
COVIDhub-baseline	82.469	139.727	59.058	101.000	0.916	0.763
GT-DeepCOVID	99.027	177.124	75.166	127.312	0.894	0.762
JHUAPL-Bucky	161.293	354.549	112.632	211.784	0.949	**0.901**

Table S31. Comparison of different methods for state-level COVID-19 1 to 2 weeks ahead hospitalizations predictions in Oregon (OR). The MSE, MAE, and correlation are reported and best performed method is highlighted in boldface.

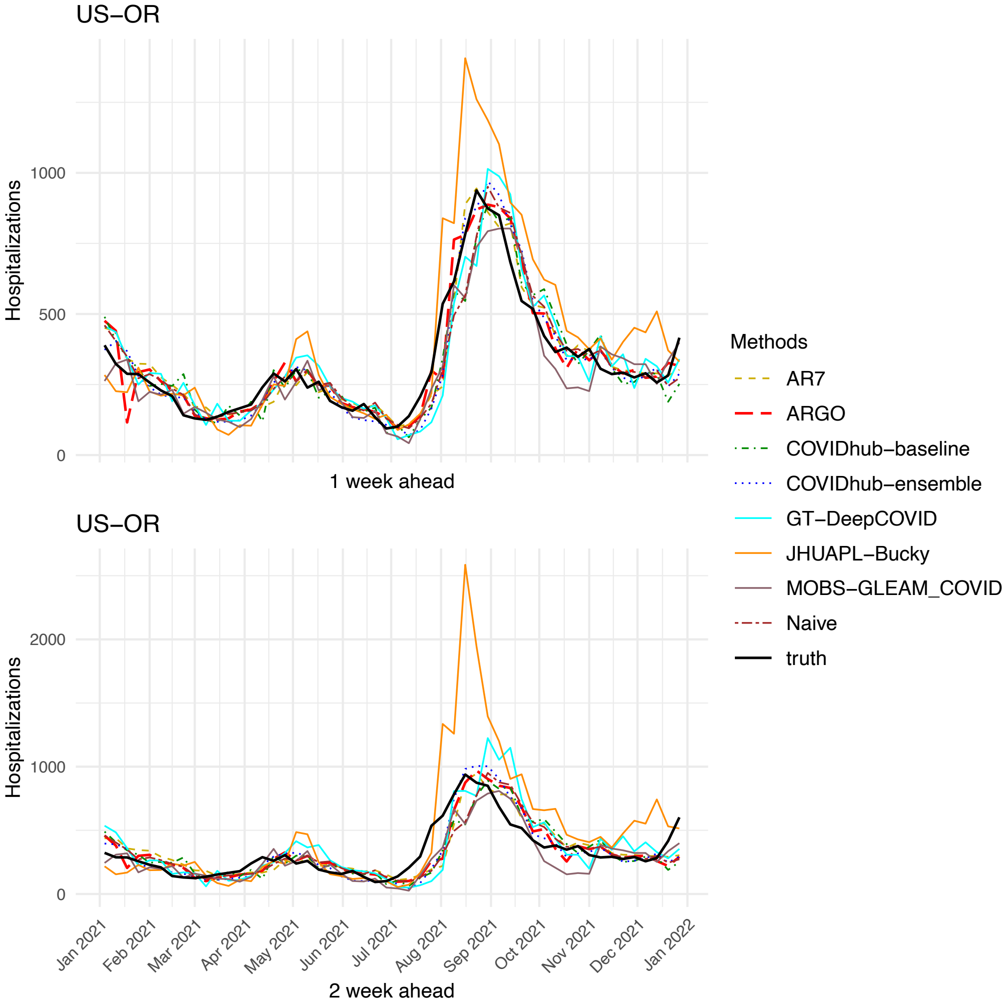

Figure S28. Plots of the COVID-19 hospitalizations 1 week (top), 2 weeks (bottom) ahead estimates of all compared models for New Oregon (OR).

Methods	RMSE		MAE		Cor	
	1 Week Ahead	2 Week Ahead	1 Week Ahead	2 Week Ahead	1 Week Ahead	2 Week Ahead
ARGO	140.275	253.954	84.862	158.993	0.959	0.889
AR7	145.483	270.938	82.568	159.024	0.955	0.870
COVIDhub-ensemble	**132.464**	**233.235**	**74.058**	**142.731**	**0.964**	**0.908**
Naive	175.455	321.447	105.115	197.462	0.935	0.814
MOBS-GLEAM_COVID	178.676	290.679	122.435	194.646	0.933	0.853
COVIDhub-baseline	169.419	300.656	104.519	191.904	0.938	0.837
GT-DeepCOVID	205.979	340.541	129.320	230.928	0.915	0.803
JHUAPL-Bucky	157.056	259.875	91.521	159.747	0.948	0.891

Table S32. Comparison of different methods for state-level COVID-19 1 to 2 weeks ahead hospitalizations predictions in Massachusetts (MA). The MSE, MAE, and correlation are reported and best performed method is highlighted in boldface.

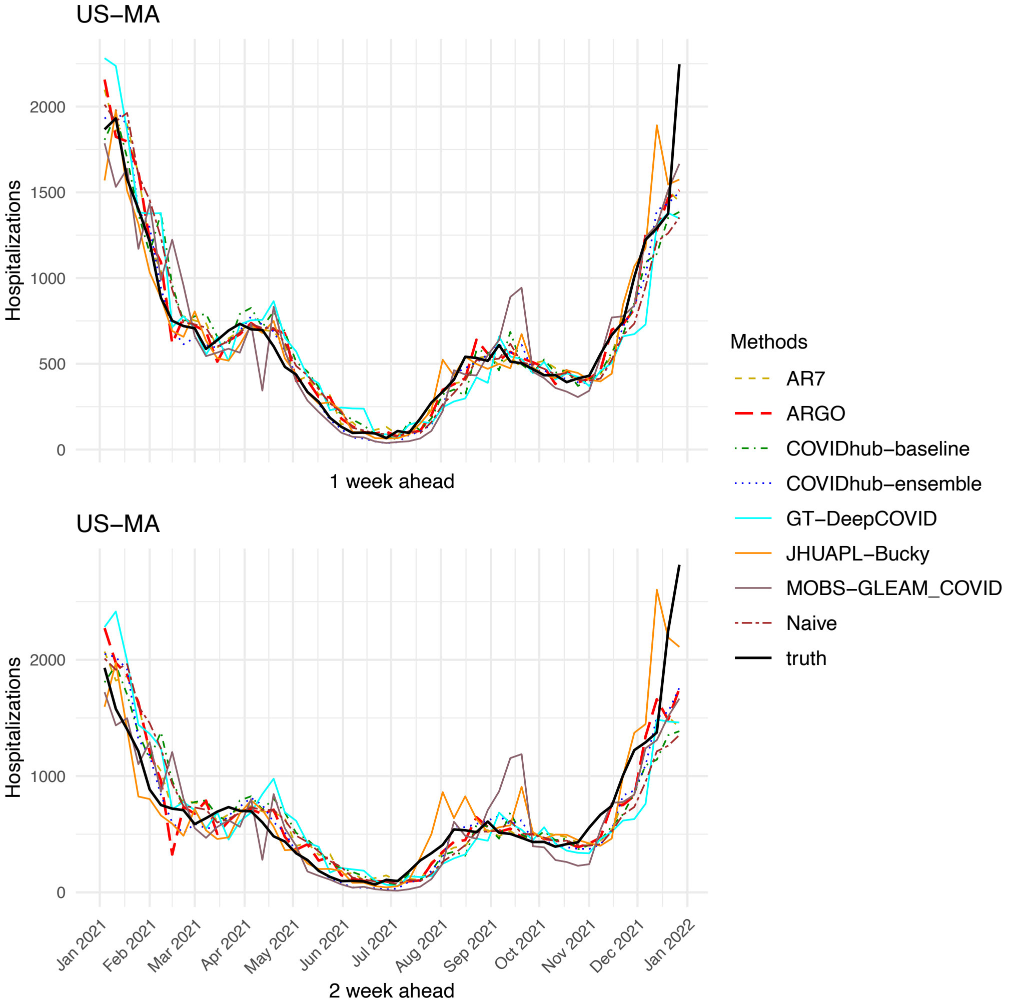

Figure S29. Plots of the COVID-19 hospitalizations 1 week (top), 2 weeks (bottom) ahead estimates of all compared models for New Massachusetts (MA).

Methods	RMSE		MAE		Cor	
	1 Week Ahead	2 Week Ahead	1 Week Ahead	2 Week Ahead	1 Week Ahead	2 Week Ahead
ARGO	**93.288**	**160.449**	**69.217**	**131.633**	**0.973**	**0.917**
AR7	102.632	198.858	81.008	150.721	0.964	0.855
COVIDhub-ensemble	116.690	191.548	85.654	132.365	0.960	0.892
Naive	119.640	220.201	89.846	169.481	0.946	0.816
MOBS-GLEAM_COVID	186.978	274.706	132.295	195.205	0.896	0.771
COVIDhub-baseline	152.599	239.035	113.769	186.442	0.920	0.796
GT-DeepCOVID	173.042	279.126	131.280	206.161	0.915	0.788
JHUAPL-Bucky	215.347	297.893	156.615	220.588	0.880	0.800

Table S33. Comparison of different methods for state-level COVID-19 1 to 2 weeks ahead hospitalizations predictions in Arkansas (AR). The MSE, MAE, and correlation are reported and best performed method is highlighted in boldface.

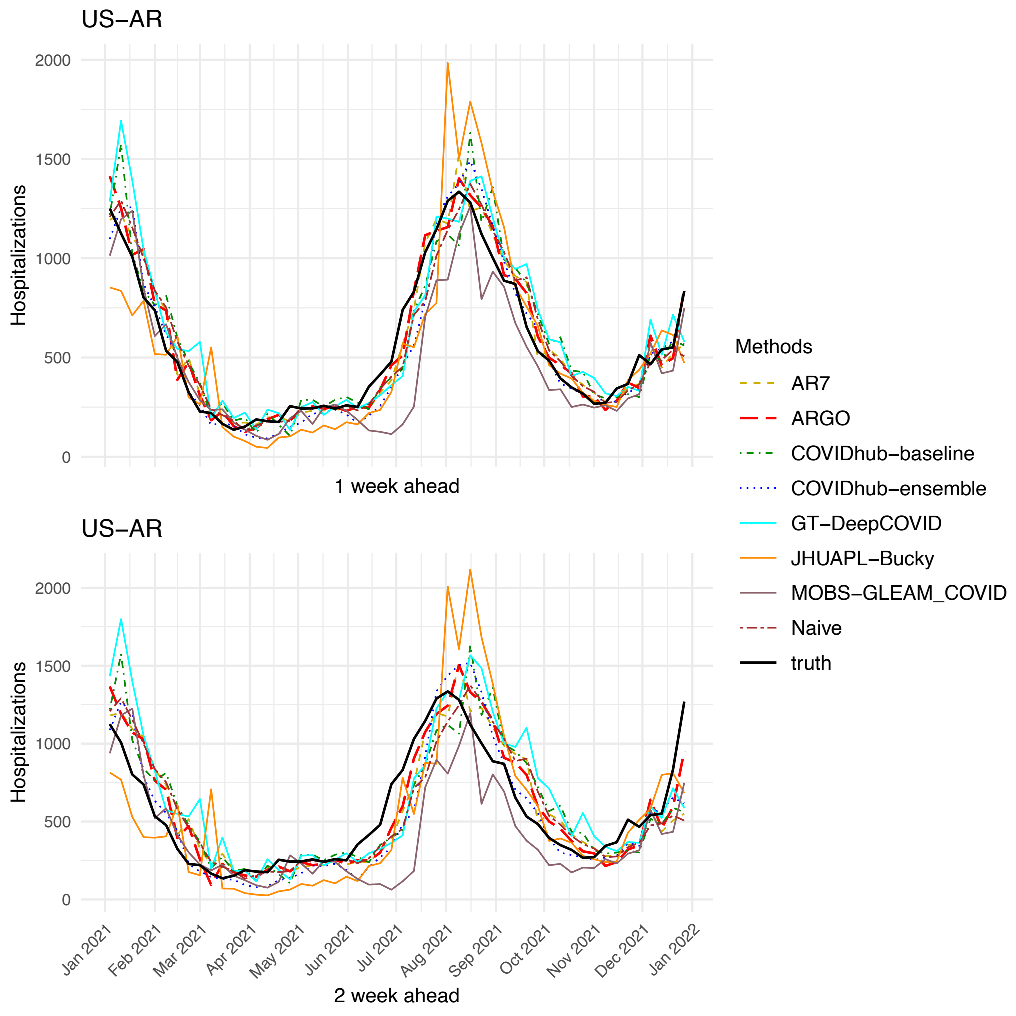

Figure S30. Plots of the COVID-19 hospitalizations 1 week (top), 2 weeks (bottom) ahead estimates of all compared models for New Arkansas (AR).

Methods	RMSE		MAE		Cor	
	1 Week Ahead	2 Week Ahead	1 Week Ahead	2 Week Ahead	1 Week Ahead	2 Week Ahead
ARGO	18.832	**25.268**	**13.442**	**19.389**	0.944	0.899
AR7	19.934	30.782	14.759	24.012	0.939	0.848
COVIDhub-ensemble	19.049	26.665	15.577	19.962	0.948	**0.907**
Naive	**17.975**	27.653	13.750	21.288	**0.949**	0.879
MOBS-GLEAM_COVID	36.580	48.489	27.378	35.389	0.773	0.598
COVIDhub-baseline	27.480	31.138	20.635	22.788	0.882	0.848
GT-DeepCOVID	33.029	45.123	24.355	34.253	0.833	0.737
JHUAPL-Bucky	40.895	62.014	26.846	40.007	0.916	0.853

Table S34. Comparison of different methods for state-level COVID-19 1 to 2 weeks ahead hospitalizations predictions in Alaska (AK). The MSE, MAE, and correlation are reported and best performed method is highlighted in boldface.

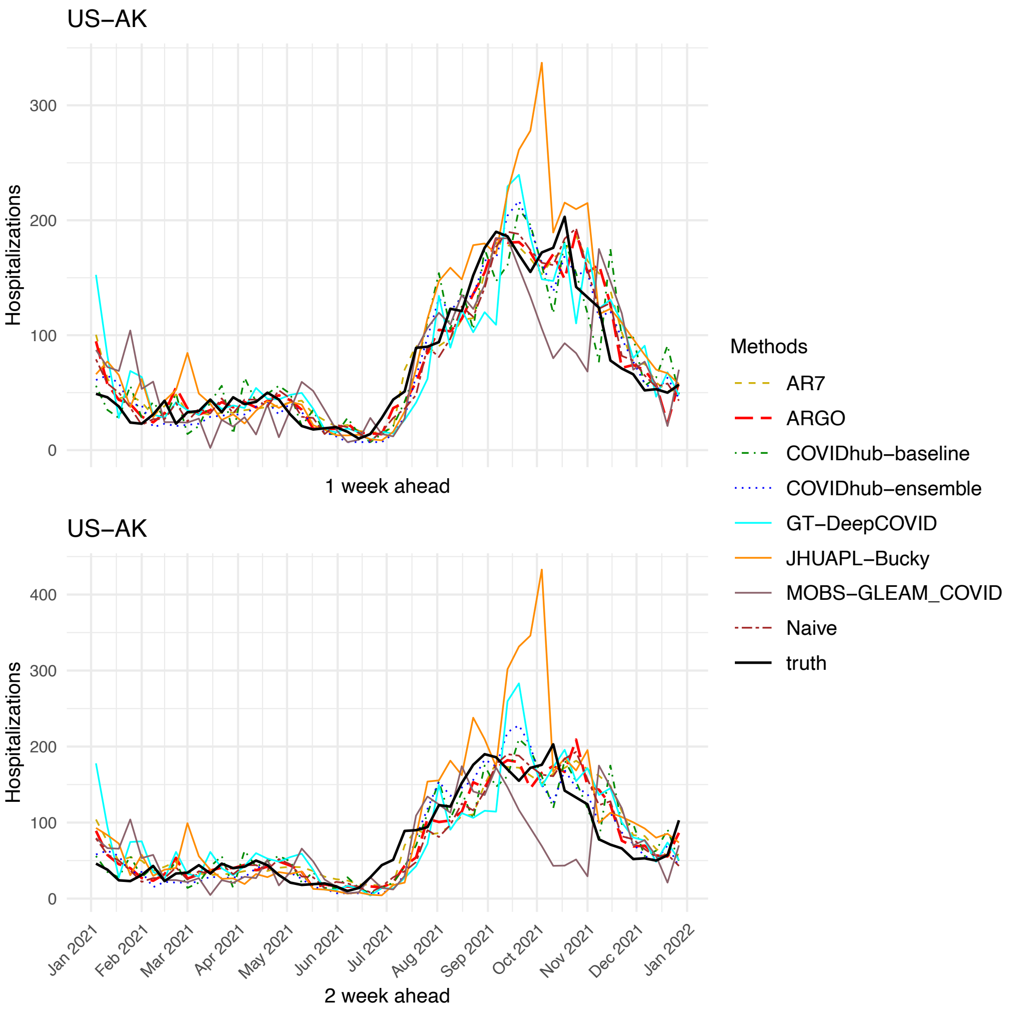

Figure S31. Plots of the COVID-19 hospitalizations 1 week (top), 2 weeks (bottom) ahead estimates of all compared models for New Alaska (AK).

Methods	RMSE		MAE		Cor	
	1 Week Ahead	2 Week Ahead	1 Week Ahead	2 Week Ahead	1 Week Ahead	2 Week Ahead
ARGO	**241.217**	**559.846**	**182.339**	**408.646**	**0.985**	**0.930**
AR7	301.667	640.456	214.845	470.570	0.976	0.910
COVIDhub-ensemble	408.214	634.251	255.462	419.615	0.958	0.917
Naive	422.981	747.374	306.115	564.692	0.953	0.876
MOBS-GLEAM_COVID	538.346	816.229	395.590	627.383	0.930	0.869
COVIDhub-baseline	473.253	769.653	334.769	555.308	0.941	0.871
GT-DeepCOVID	404.838	768.588	274.394	543.376	0.956	0.873
JHUAPL-Bucky	560.093	756.705	430.132	552.097	0.929	0.917

Table S35. Comparison of different methods for state-level COVID-19 1 to 2 weeks ahead hospitalizations predictions in Ohio (OH). The MSE, MAE, and correlation are reported and best performed method is highlighted in boldface.

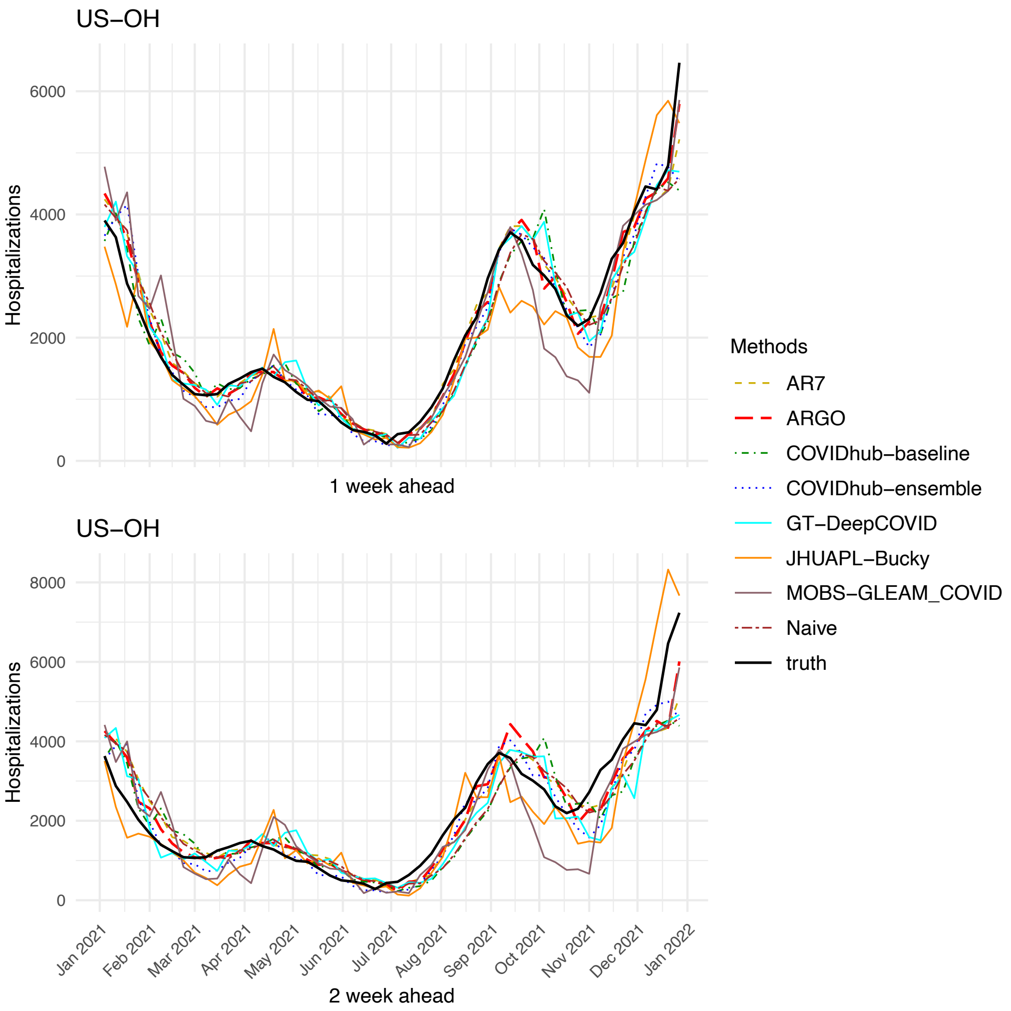

Figure S32. Plots of the COVID-19 hospitalizations 1 week (top), 2 weeks (bottom) ahead estimates of all compared models for New Ohio (OH).

Methods	RMSE		MAE		Cor	
	1 Week Ahead	2 Week Ahead	1 Week Ahead	2 Week Ahead	1 Week Ahead	2 Week Ahead
ARGO	**47.537**	**90.427**	**36.538**	**73.412**	**0.974**	0.909
AR7	55.714	100.311	45.173	78.422	0.960	0.871
COVIDhub-ensemble	65.042	107.526	46.923	77.712	0.965	**0.912**
Naive	67.355	114.326	52.981	90.750	0.943	0.839
MOBS-GLEAM_COVID	73.086	106.901	55.463	80.139	0.947	0.899
COVIDhub-baseline	89.307	130.608	65.962	101.923	0.923	0.823
GT-DeepCOVID	83.356	130.505	67.665	102.860	0.917	0.826
JHUAPL-Bucky	161.839	248.272	110.168	171.911	0.884	0.844

Table S36. Comparison of different methods for state-level COVID-19 1 to 2 weeks ahead hospitalizations predictions in West Virginia (WV). The MSE, MAE, and correlation are reported and best performed method is highlighted in boldface.

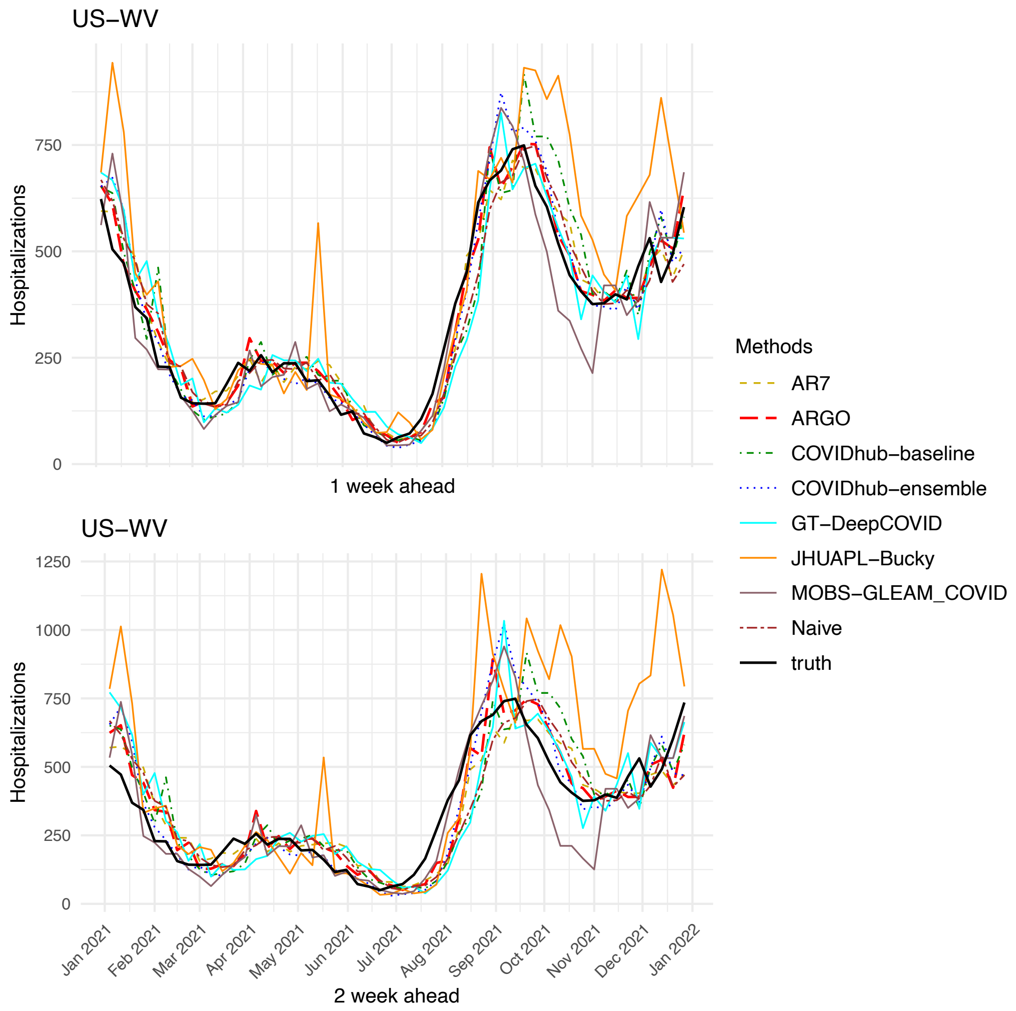

Figure S33. Plots of the COVID-19 hospitalizations 1 week (top), 2 weeks (bottom) ahead estimates of all compared models for New West Virginia (WV).

Methods	RMSE		MAE		Cor	
	1 Week Ahead	2 Week Ahead	1 Week Ahead	2 Week Ahead	1 Week Ahead	2 Week Ahead
ARGO	31.523	**39.868**	20.774	29.749	**0.928**	**0.874**
AR7	29.258	48.270	20.759	34.960	0.926	0.793
COVIDhub-ensemble	33.487	42.151	24.750	30.827	0.915	0.865
Naive	**28.912**	41.082	**19.481**	**29.577**	**0.928**	0.855
MOBS-GLEAM_COVID	42.033	56.486	30.655	41.229	0.844	0.719
COVIDhub-baseline	35.713	45.431	27.654	33.135	0.905	0.843
GT-DeepCOVID	45.661	61.359	33.846	47.373	0.836	0.735
JHUAPL-Bucky	44.127	53.392	33.299	39.225	0.854	0.769

Table S37. Comparison of different methods for state-level COVID-19 1 to 2 weeks ahead hospitalizations predictions in Wyoming (WY). The MSE, MAE, and correlation are reported and best performed method is highlighted in boldface.

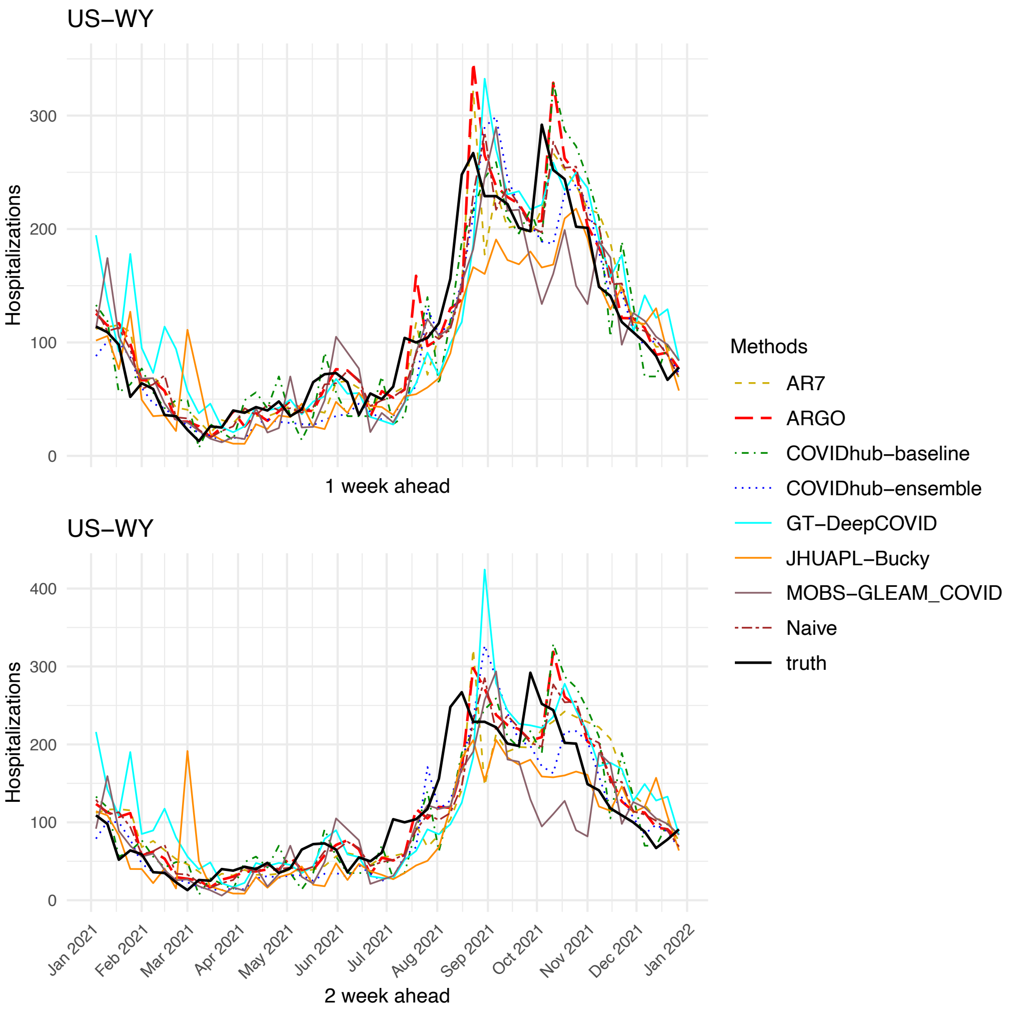

Figure S34. Plots of the COVID-19 hospitalizations 1 week (top), 2 weeks (bottom) ahead estimates of all compared models for New Wyoming (WY).

Methods	RMSE		MAE		Cor	
	1 Week Ahead	2 Week Ahead	1 Week Ahead	2 Week Ahead	1 Week Ahead	2 Week Ahead
ARGO	**95.493**	**170.424**	**51.479**	**107.810**	0.955	0.892
AR7	133.967	240.808	71.178	127.096	0.907	0.768
COVIDhub-ensemble	126.391	192.531	69.904	116.577	0.923	0.871
Naive	150.763	253.019	78.731	145.019	0.882	0.744
MOBS-GLEAM_COVID	95.790	175.794	75.205	113.623	**0.956**	**0.895**
COVIDhub-baseline	168.271	259.005	99.231	158.096	0.851	0.727
GT-DeepCOVID	174.089	266.976	99.549	158.615	0.842	0.715
JHUAPL-Bucky	139.367	188.020	85.753	129.321	0.904	0.882

Table S38. Comparison of different methods for state-level COVID-19 1 to 2 weeks ahead hospitalizations predictions in Connecticut (CT). The MSE, MAE, and correlation are reported and best performed method is highlighted in boldface.

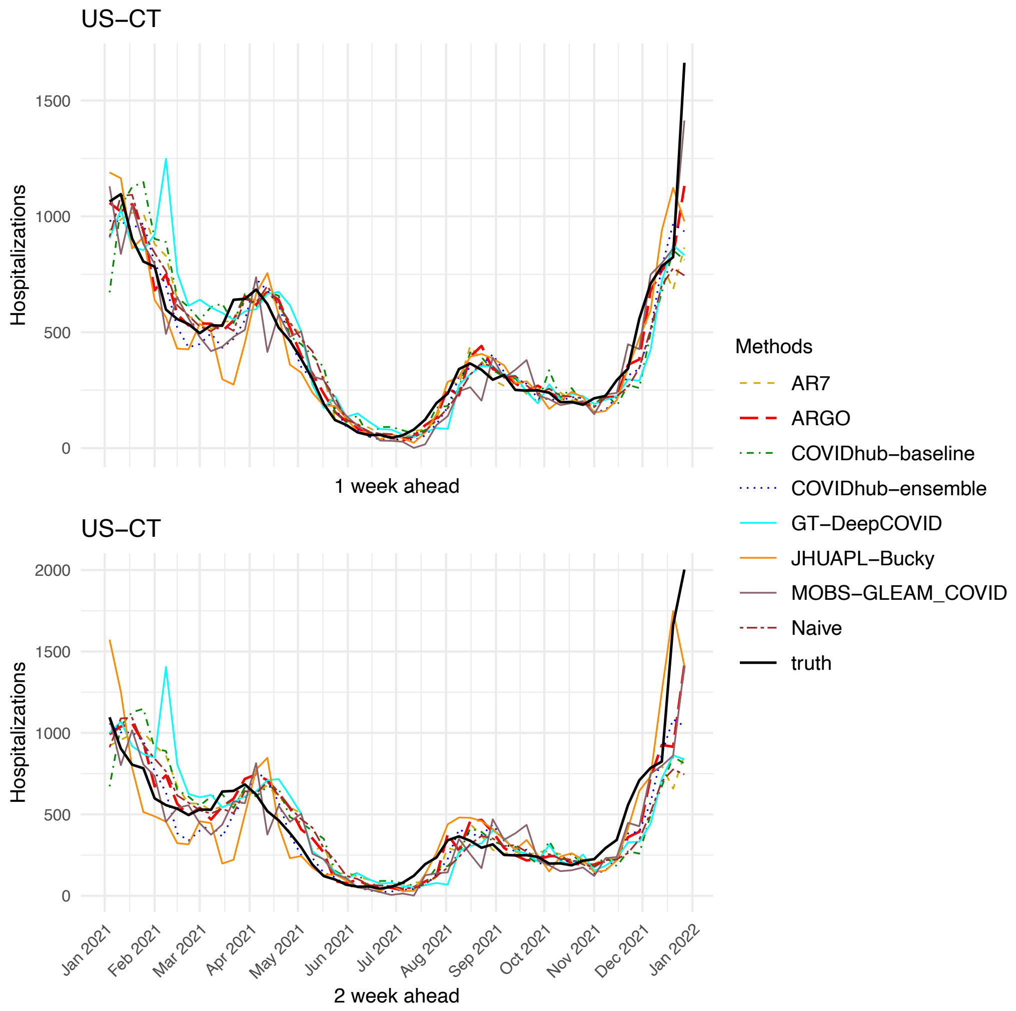

Figure S35. Plots of the COVID-19 hospitalizations 1 week (top), 2 weeks (bottom) ahead estimates of all compared models for New Connecticut (CT).

Methods	RMSE		MAE		Cor	
	1 Week Ahead	2 Week Ahead	1 Week Ahead	2 Week Ahead	1 Week Ahead	2 Week Ahead
ARGO	**294.078**	768.804	**201.508**	**480.730**	**0.974**	**0.875**
AR7	298.662	**650.740**	223.916	505.674	0.969	0.847
COVIDhub-ensemble	477.592	780.033	305.250	500.654	0.929	0.825
Naive	437.563	752.901	313.827	571.346	0.926	0.790
MOBS-GLEAM_COVID	685.047	1351.964	426.187	750.286	0.893	0.706
COVIDhub-baseline	485.242	794.124	335.038	580.865	0.910	0.768
GT-DeepCOVID	490.180	857.363	356.471	641.452	0.919	0.752
JHUAPL-Bucky	868.019	1386.654	525.806	786.233	0.868	0.769

Table S39. Comparison of different methods for state-level COVID-19 1 to 2 weeks ahead hospitalizations predictions in Michigan (MI). The MSE, MAE, and correlation are reported and best performed method is highlighted in boldface.

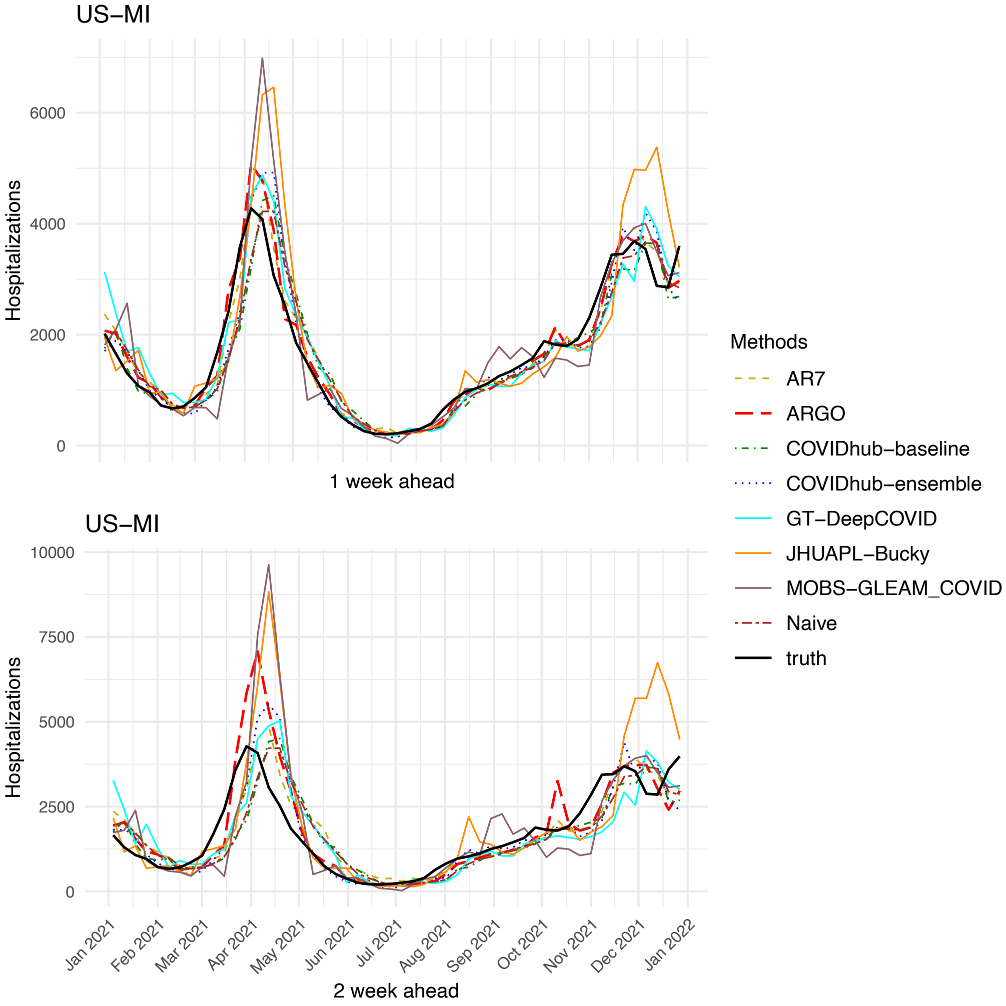

Figure S36. Plots of the COVID-19 hospitalizations 1 week (top), 2 weeks (bottom) ahead estimates of all compared models for New Michigan (MI).

Methods	RMSE		MAE		Cor	
	1 Week Ahead	2 Week Ahead	1 Week Ahead	2 Week Ahead	1 Week Ahead	2 Week Ahead
ARGO	**234.507**	558.415	**162.766**	**297.625**	**0.971**	0.881
AR7	260.853	628.973	165.250	348.374	0.965	0.849
COVIDhub-ensemble	409.691	625.172	208.250	344.827	0.913	0.858
Naive	464.845	804.344	231.269	432.423	0.886	0.739
MOBS-GLEAM_COVID	365.702	736.589	261.375	464.496	0.931	0.785
COVIDhub-baseline	497.509	813.902	266.000	451.769	0.866	0.730
GT-DeepCOVID	342.160	684.912	238.350	447.842	0.942	0.816
JHUAPL-Bucky	407.962	**546.555**	220.364	334.957	0.910	**0.886**

Table S40. Comparison of different methods for state-level COVID-19 1 to 2 weeks ahead hospitalizations predictions in New Jersey (NJ). The MSE, MAE, and correlation are reported and best performed method is highlighted in boldface.

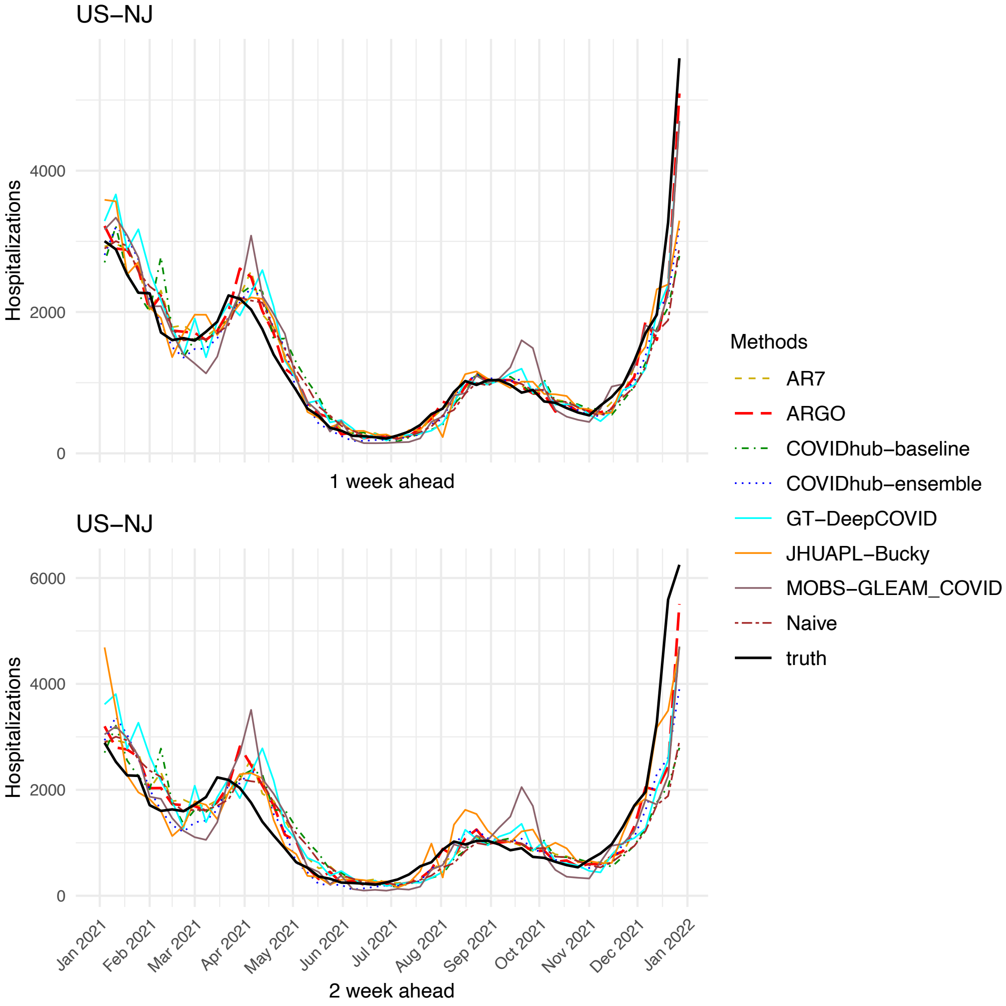

Figure S37. Plots of the COVID-19 hospitalizations 1 week (top), 2 weeks (bottom) ahead estimates of all compared models for New New Jersey (NJ).

Methods	RMSE		MAE		Cor	
	1 Week Ahead	2 Week Ahead	1 Week Ahead	2 Week Ahead	1 Week Ahead	2 Week Ahead
ARGO	205.911	432.673	**148.668**	310.243	**0.969**	0.862
AR7	**205.837**	435.616	156.040	333.226	**0.969**	0.832
COVIDhub-ensemble	259.060	429.272	175.692	**286.577**	0.951	**0.872**
Naive	292.678	525.504	216.058	387.519	0.919	0.740
MOBS-GLEAM_COVID	270.064	**406.380**	206.227	307.255	0.938	0.870
COVIDhub-baseline	572.630	600.462	329.173	394.788	0.661	0.622
GT-DeepCOVID	342.450	595.482	256.920	430.579	0.903	0.746
JHUAPL-Bucky	463.969	598.101	334.789	399.626	0.885	0.859

Table S41. Comparison of different methods for state-level COVID-19 1 to 2 weeks ahead hospitalizations predictions in Tennessee (TN). The MSE, MAE, and correlation are reported and best performed method is highlighted in boldface.

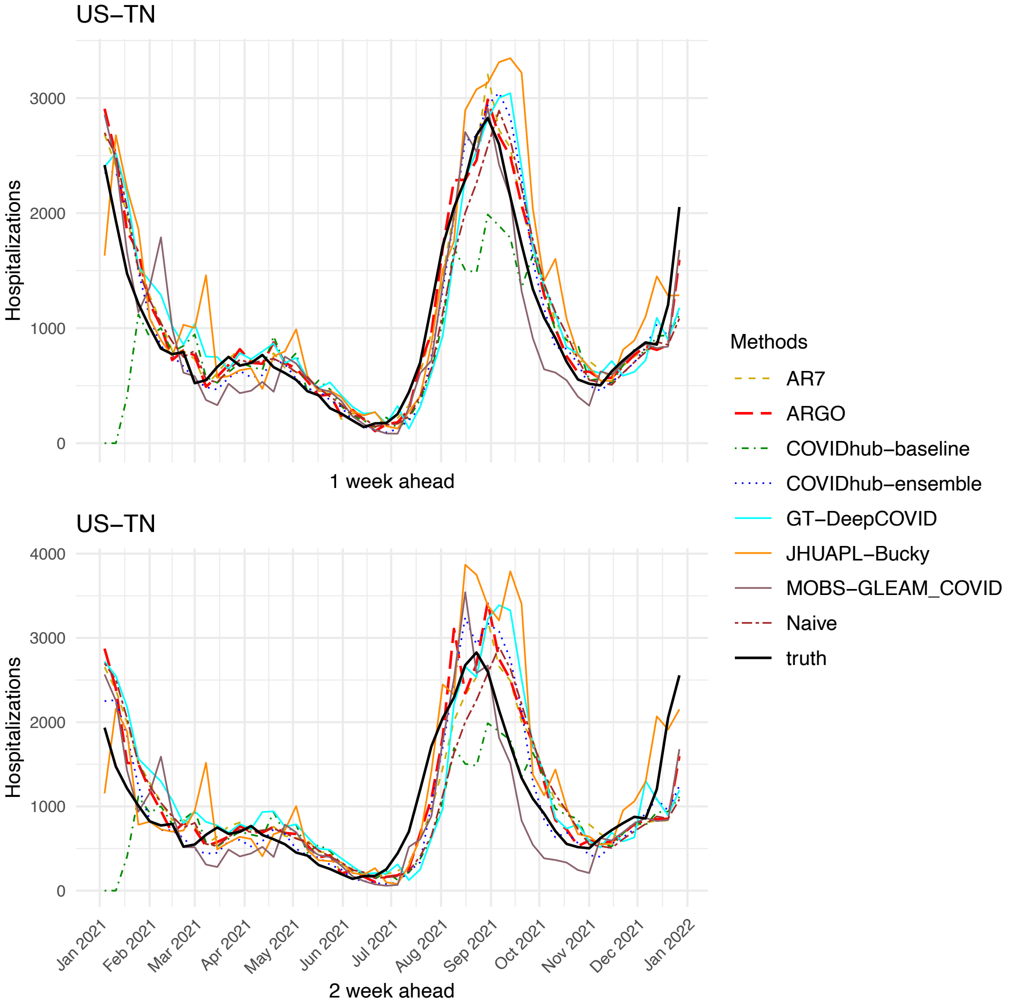

Figure S38. Plots of the COVID-19 hospitalizations 1 week (top), 2 weeks (bottom) ahead estimates of all compared models for New Tennessee (TN).

Methods	RMSE		MAE		Cor	
	1 Week Ahead	2 Week Ahead	1 Week Ahead	2 Week Ahead	1 Week Ahead	2 Week Ahead
ARGO	**164.989**	379.966	**109.513**	**238.075**	**0.987**	**0.927**
AR7	175.915	**375.124**	123.950	246.940	0.983	0.912
COVIDhub-ensemble	258.440	461.810	160.558	266.481	0.974	0.915
Naive	252.887	463.828	171.808	303.385	0.967	0.881
MOBS-GLEAM_COVID	360.336	586.952	257.392	417.970	0.949	0.864
COVIDhub-baseline	261.054	445.984	179.038	296.962	0.963	0.887
GT-DeepCOVID	228.603	435.147	183.521	332.371	0.971	0.900
JHUAPL-Bucky	440.951	693.221	286.634	437.574	0.947	0.901

Table S42. Comparison of different methods for state-level COVID-19 1 to 2 weeks ahead hospitalizations predictions in Arizona (AZ). The MSE, MAE, and correlation are reported and best performed method is highlighted in boldface.

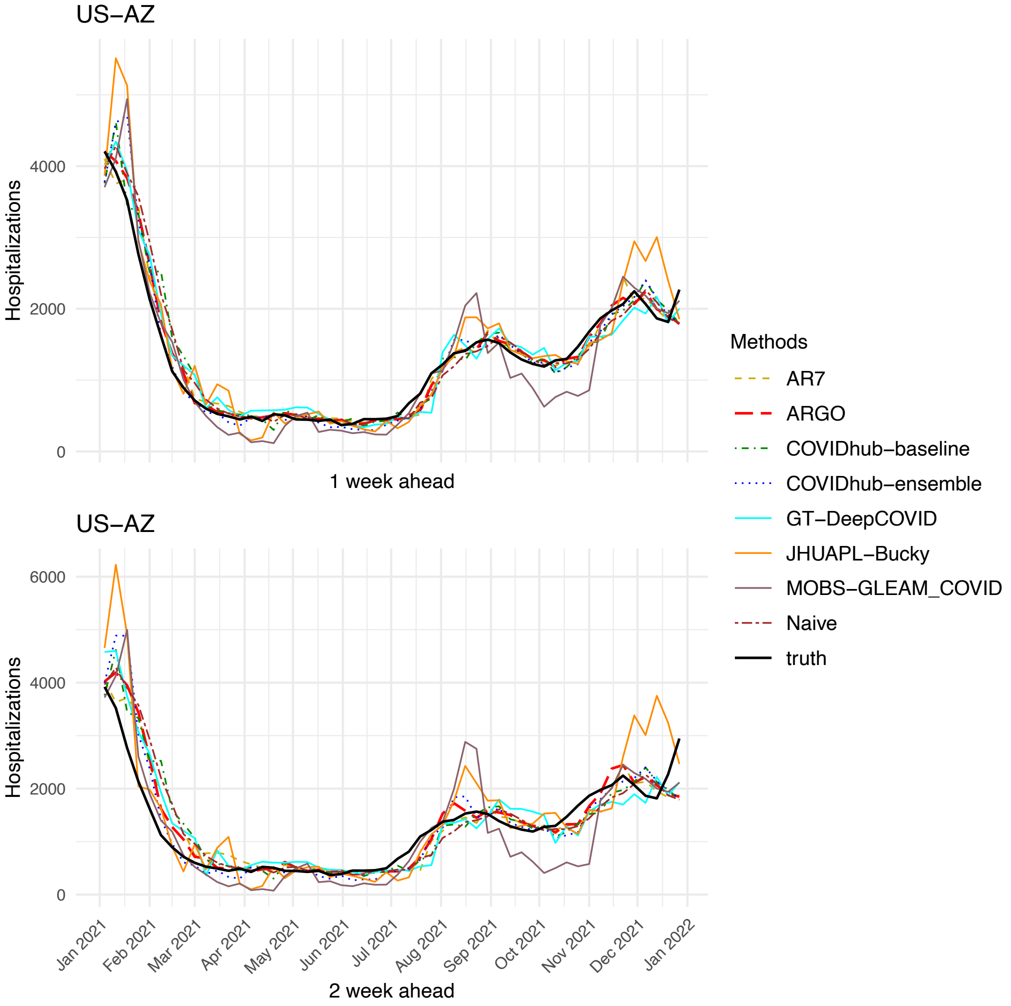

Figure S39. Plots of the COVID-19 hospitalizations 1 week (top), 2 weeks (bottom) ahead estimates of all compared models for New Arizona (AZ).

Methods	RMSE		MAE		Cor	
	1 Week Ahead	2 Week Ahead	1 Week Ahead	2 Week Ahead	1 Week Ahead	2 Week Ahead
ARGO	**694.280**	1926.407	**475.535**	**1275.348**	**0.989**	**0.893**
AR7	803.686	1936.963	610.086	1350.937	0.978	0.862
COVIDhub-ensemble	1420.437	2426.737	932.019	1570.846	0.941	0.844
Naive	1350.872	2510.225	924.769	1709.962	0.933	0.769
MOBS-GLEAM_COVID	1017.099	**1843.362**	749.273	1309.635	0.963	0.883
COVIDhub-baseline	1487.790	2609.230	1012.923	1809.846	0.920	0.752
GT-DeepCOVID	1409.907	2480.871	986.914	1726.428	0.927	0.792
JHUAPL-Bucky	2758.649	3793.091	1942.424	2685.922	0.836	0.770

Table S43. Comparison of different methods for state-level COVID-19 1 to 2 weeks ahead hospitalizations predictions in Texas (TX). The MSE, MAE, and correlation are reported and best performed method is highlighted in boldface.

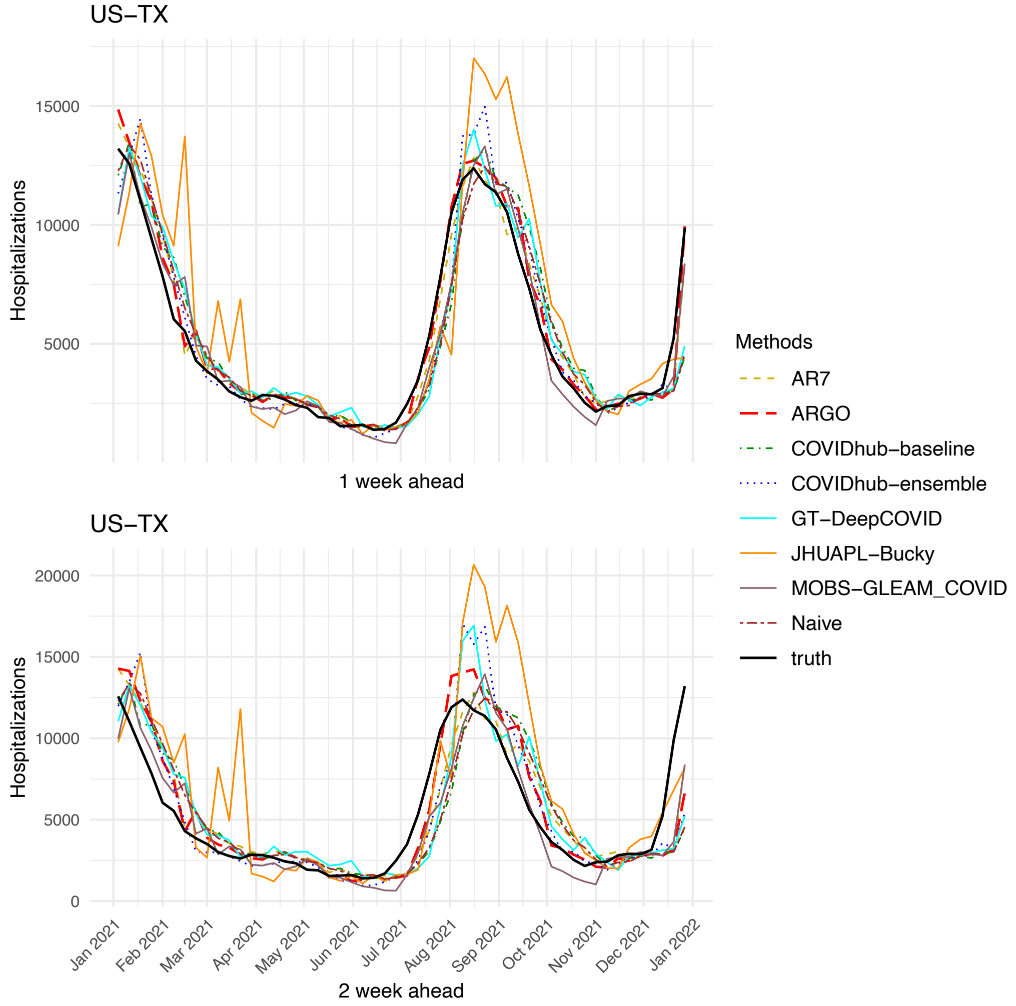

Figure S40. Plots of the COVID-19 hospitalizations 1 week (top), 2 weeks (bottom) ahead estimates of all compared models for New Texas (TX).

Methods	RMSE		MAE		Cor	
	1 Week Ahead	2 Week Ahead	1 Week Ahead	2 Week Ahead	1 Week Ahead	2 Week Ahead
ARGO	**558.546**	**1630.819**	**330.790**	**896.587**	**0.990**	**0.895**
AR7	713.487	1783.270	460.100	1099.391	0.982	0.857
COVIDhub-ensemble	1269.816	2349.889	725.038	1250.712	0.960	0.833
Naive	1117.565	2239.017	698.269	1366.692	0.956	0.794
MOBS-GLEAM_COVID	1430.195	2242.263	1001.432	1500.074	0.929	0.800
COVIDhub-baseline	1043.072	2146.711	667.115	1341.577	0.957	0.797
GT-DeepCOVID	1327.192	2632.167	776.532	1652.832	0.950	0.801
JHUAPL-Bucky	3425.902	4010.325	1636.756	2086.601	0.907	0.821

Table S44. Comparison of different methods for state-level COVID-19 1 to 2 weeks ahead hospitalizations predictions in California (CA). The MSE, MAE, and correlation are reported and best performed method is highlighted in boldface.

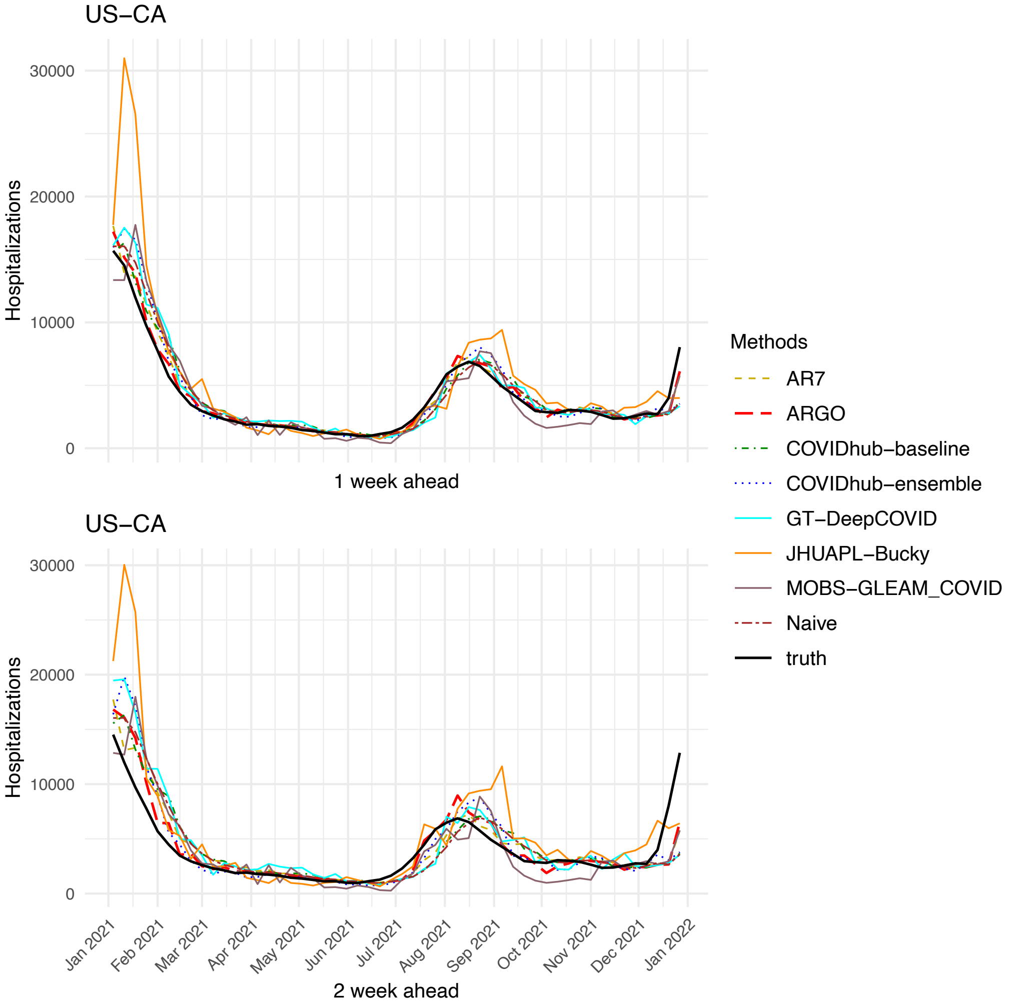

Figure S41. Plots of the COVID-19 hospitalizations 1 week (top), 2 weeks (bottom) ahead estimates of all compared models for New California (CA).

Methods	RMSE		MAE		Cor	
	1 Week Ahead	2 Week Ahead	1 Week Ahead	2 Week Ahead	1 Week Ahead	2 Week Ahead
ARGO	**259.858**	**564.317**	**191.719**	409.134	**0.974**	**0.894**
AR7	333.522	649.426	229.746	481.142	0.957	0.856
COVIDhub-ensemble	407.388	646.373	245.327	**394.269**	0.935	0.863
Naive	404.332	703.372	289.077	527.423	0.935	0.830
MOBS-GLEAM_COVID	392.792	721.467	298.344	518.342	0.947	0.844
COVIDhub-baseline	467.962	732.576	308.154	503.038	0.912	0.815
GT-DeepCOVID	560.011	874.646	365.580	586.065	0.881	0.752
JHUAPL-Bucky	647.529	965.349	433.610	648.434	0.910	0.881

Table S45. Comparison of different methods for state-level COVID-19 1 to 2 weeks ahead hospitalizations predictions in Pennsylvania (PA). The MSE, MAE, and correlation are reported and best performed method is highlighted in boldface.

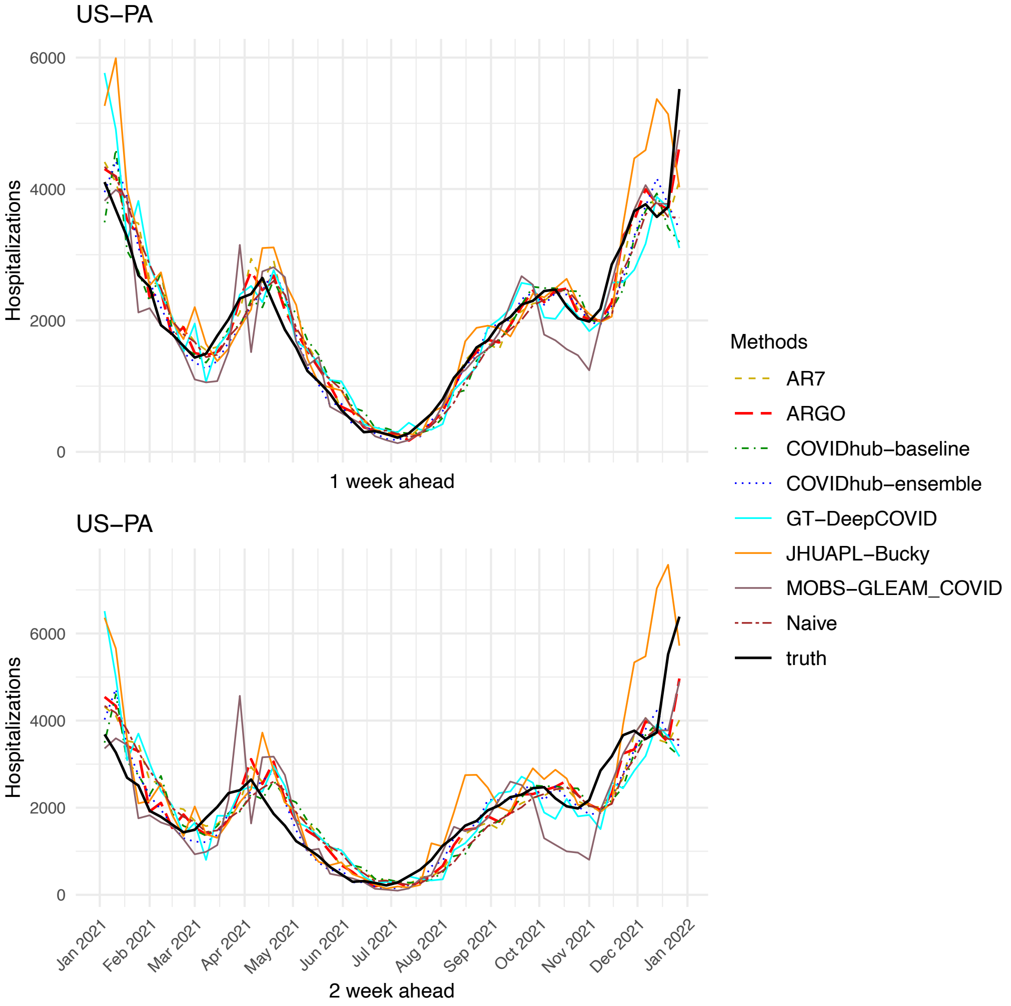

Figure S42. Plots of the COVID-19 hospitalizations 1 week (top), 2 weeks (bottom) ahead estimates of all compared models for New Pennsylvania (PA).

Methods	RMSE		MAE		Cor	
	1 Week Ahead	2 Week Ahead	1 Week Ahead	2 Week Ahead	1 Week Ahead	2 Week Ahead
ARGO	215.699	**434.108**	**159.364**	**330.164**	0.979	0.929
AR7	**204.355**	504.545	163.034	393.979	**0.980**	0.912
COVIDhub-ensemble	312.647	482.454	219.981	357.577	0.956	0.924
Naive	376.284	679.884	262.000	493.308	0.935	0.839
MOBS-GLEAM_COVID	303.902	564.891	247.513	439.540	0.963	0.913
COVIDhub-baseline	398.236	670.867	260.673	473.404	0.925	0.847
GT-DeepCOVID	446.429	718.916	338.989	576.896	0.891	0.790
JHUAPL-Bucky	325.060	537.848	258.863	424.967	0.948	**0.949**

Table S46. Comparison of different methods for state-level COVID-19 1 to 2 weeks ahead hospitalizations predictions in Illinois (IL). The MSE, MAE, and correlation are reported and best performed method is highlighted in boldface.

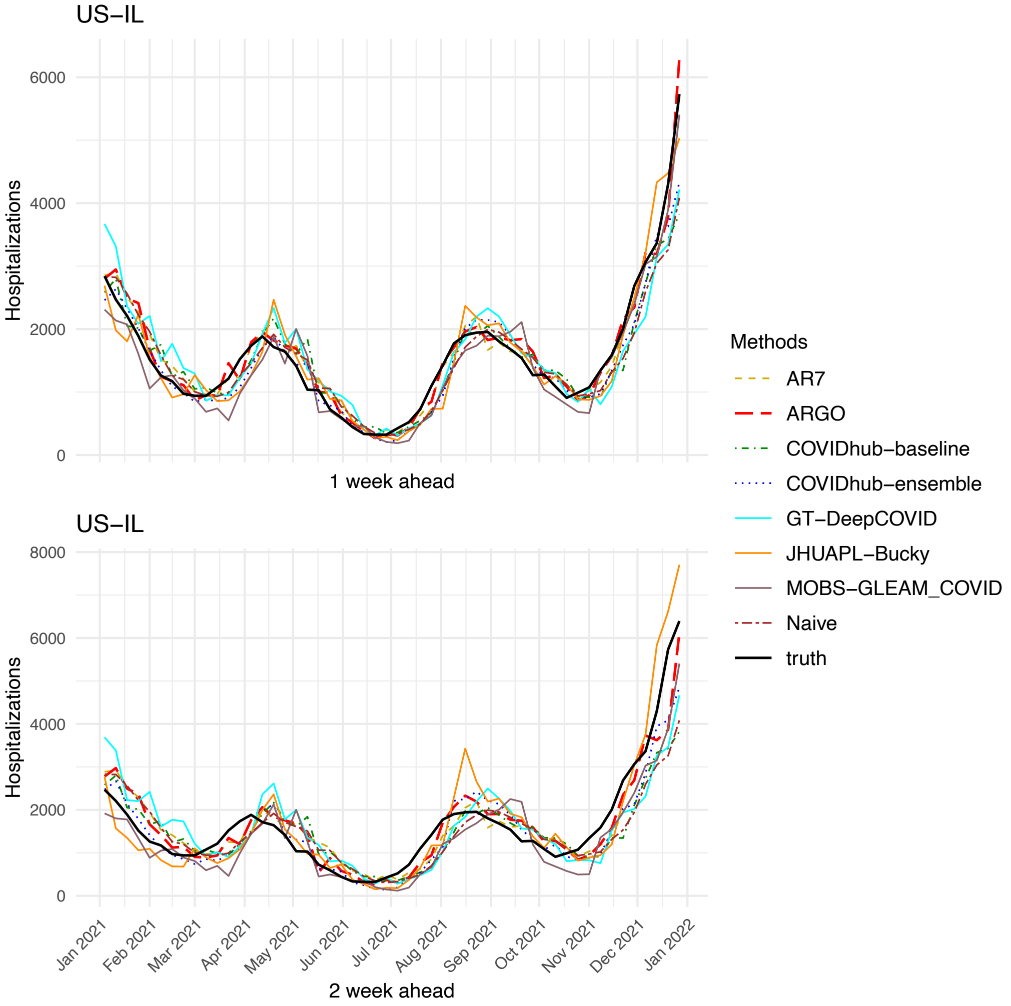

Figure S43. Plots of the COVID-19 hospitalizations 1 week (top), 2 weeks (bottom) ahead estimates of all compared models for New Illinois (IL).

Methods	RMSE		MAE		Cor	
	1 Week Ahead	2 Week Ahead	1 Week Ahead	2 Week Ahead	1 Week Ahead	2 Week Ahead
ARGO	199.557	487.760	132.192	307.975	0.971	0.814
AR7	**169.264**	**437.288**	**117.656**	**289.906**	**0.973**	0.812
COVIDhub-ensemble	355.698	595.532	209.231	325.673	0.908	0.774
Naive	312.898	567.201	198.788	360.615	0.905	0.695
MOBS-GLEAM_COVID	351.671	604.996	197.005	327.721	0.944	**0.845**
COVIDhub-baseline	334.153	571.009	219.173	363.423	0.889	0.684
GT-DeepCOVID	356.651	673.241	236.374	421.725	0.893	0.696
JHUAPL-Bucky	461.199	719.550	277.292	420.004	0.871	0.734

Table S47. Comparison of different methods for state-level COVID-19 1 to 2 weeks ahead hospitalizations predictions in Louisiana (LA). The MSE, MAE, and correlation are reported and best performed method is highlighted in boldface.

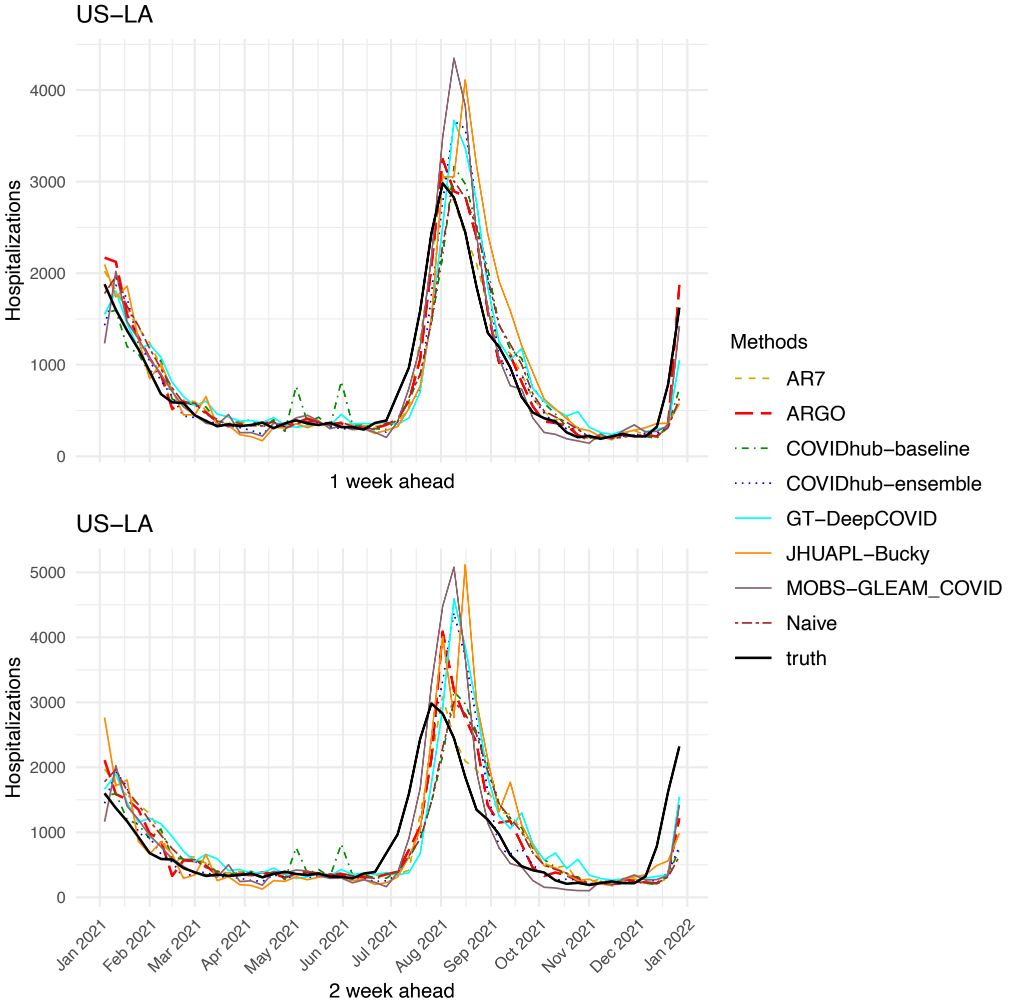

Figure S44. Plots of the COVID-19 hospitalizations 1 week (top), 2 weeks (bottom) ahead estimates of all compared models for New Louisiana (LA).

Methods	RMSE		MAE		Cor	
	1 Week Ahead	2 Week Ahead	1 Week Ahead	2 Week Ahead	1 Week Ahead	2 Week Ahead
ARGO	**158.348**	349.934	**113.063**	229.415	**0.973**	0.895
AR7	173.657	380.119	127.918	272.105	0.968	0.833
COVIDhub-ensemble	236.207	396.143	153.269	248.327	0.946	0.864
Naive	238.203	443.930	162.558	307.288	0.932	0.766
MOBS-GLEAM_COVID	198.951	**318.386**	134.317	**206.750**	0.956	**0.896**
COVIDhub-baseline	263.125	454.578	185.192	322.231	0.913	0.742
GT-DeepCOVID	297.477	519.232	207.885	352.984	0.917	0.788
JHUAPL-Bucky	340.942	508.695	229.046	341.596	0.916	0.852

Table S48. Comparison of different methods for state-level COVID-19 1 to 2 weeks ahead hospitalizations predictions in South Carolina (SC). The MSE, MAE, and correlation are reported and best performed method is highlighted in boldface.

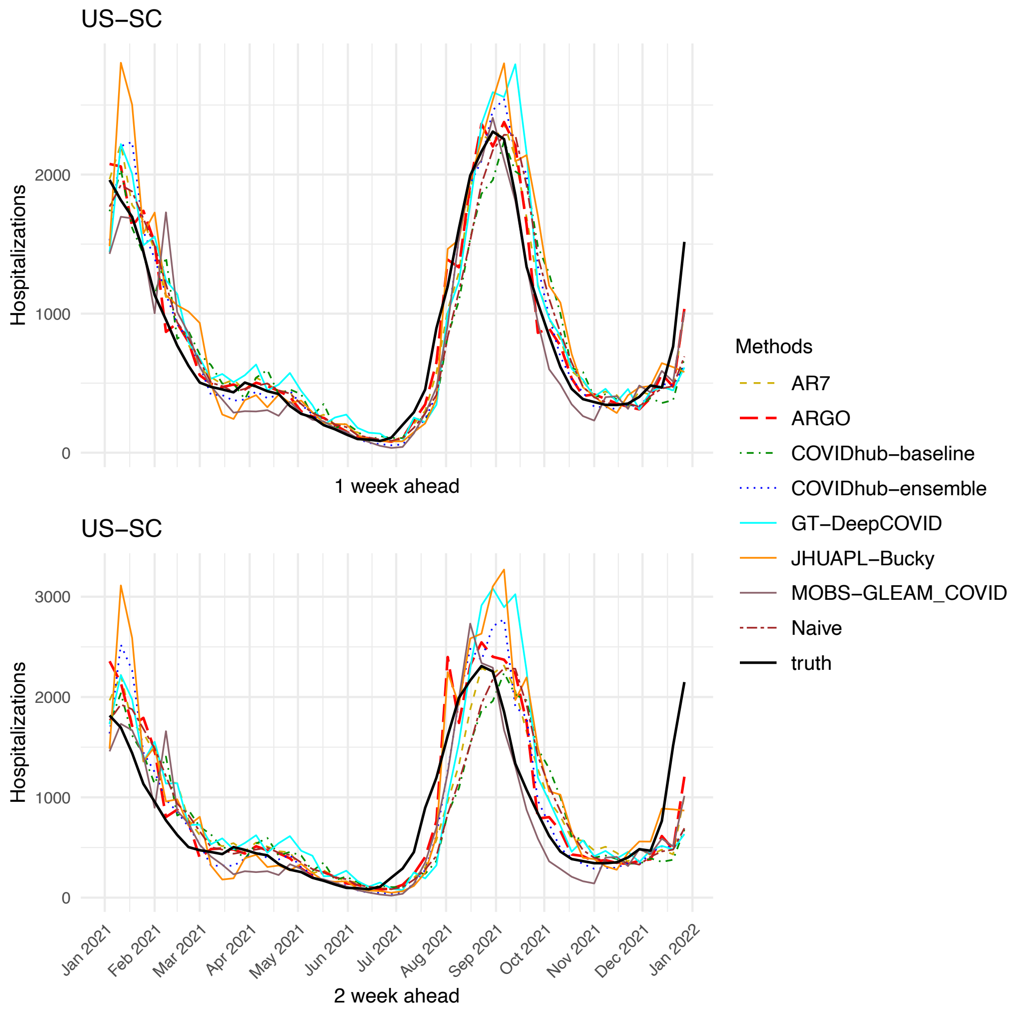

Figure S45. Plots of the COVID-19 hospitalizations 1 week (top), 2 weeks (bottom) ahead estimates of all compared models for New South Carolina (SC).

Methods	RMSE		MAE		Cor	
	1 Week Ahead	2 Week Ahead	1 Week Ahead	2 Week Ahead	1 Week Ahead	2 Week Ahead
ARGO	**100.711**	**177.897**	**68.874**	**121.079**	**0.928**	**0.824**
AR7	115.458	205.272	79.173	136.253	0.907	0.737
COVIDhub-ensemble	118.433	199.314	72.231	123.615	0.904	0.784
Naive	123.894	221.467	83.212	148.192	0.885	0.686
MOBS-GLEAM_COVID	200.373	287.254	148.837	205.179	0.702	0.500
COVIDhub-baseline	146.191	214.758	106.481	142.038	0.833	0.695
GT-DeepCOVID	138.220	243.580	103.731	180.514	0.877	0.710
JHUAPL-Bucky	281.425	593.154	207.012	373.554	0.887	0.781

Table S49. Comparison of different methods for state-level COVID-19 1 to 2 weeks ahead hospitalizations predictions in Washington (WA). The MSE, MAE, and correlation are reported and best performed method is highlighted in boldface.

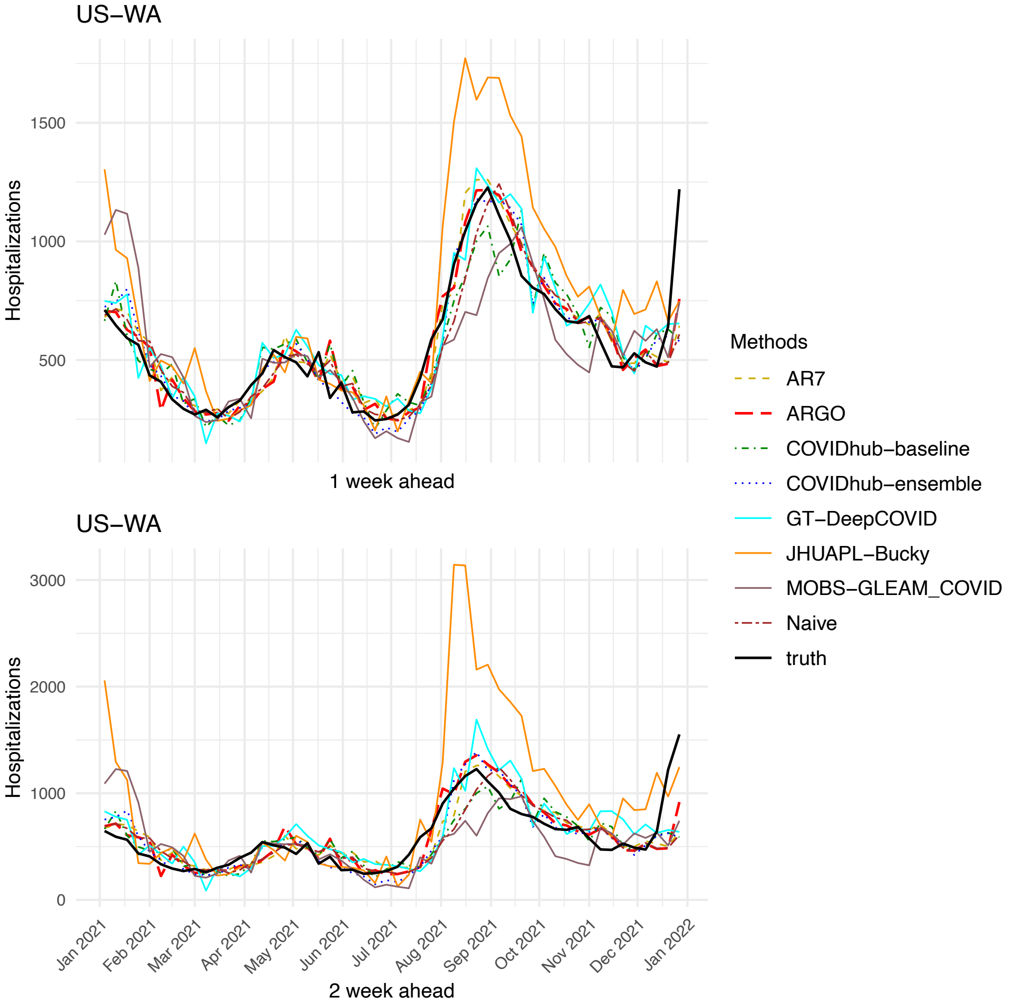

Figure S46. Plots of the COVID-19 hospitalizations 1 week (top), 2 weeks (bottom) ahead estimates of all compared models for New Washington (WA).

Methods	RMSE		MAE		Cor	
	1 Week Ahead	2 Week Ahead	1 Week Ahead	2 Week Ahead	1 Week Ahead	2 Week Ahead
ARGO	**123.723**	250.794	**78.795**	**151.552**	**0.975**	0.914
AR7	137.437	323.873	99.521	207.547	0.966	0.855
COVIDhub-ensemble	237.464	366.832	124.250	203.788	0.900	0.818
Naive	244.221	421.129	141.635	253.365	0.889	0.737
MOBS-GLEAM_COVID	195.585	359.708	151.764	250.017	0.927	0.819
COVIDhub-baseline	285.742	447.762	160.885	271.000	0.838	0.692
GT-DeepCOVID	288.326	447.215	160.238	284.163	0.834	0.690
JHUAPL-Bucky	223.484	**224.424**	137.416	165.112	0.914	**0.933**

Table S50. Comparison of different methods for state-level COVID-19 1 to 2 weeks ahead hospitalizations predictions in Maryland (MD). The MSE, MAE, and correlation are reported and best performed method is highlighted in boldface.

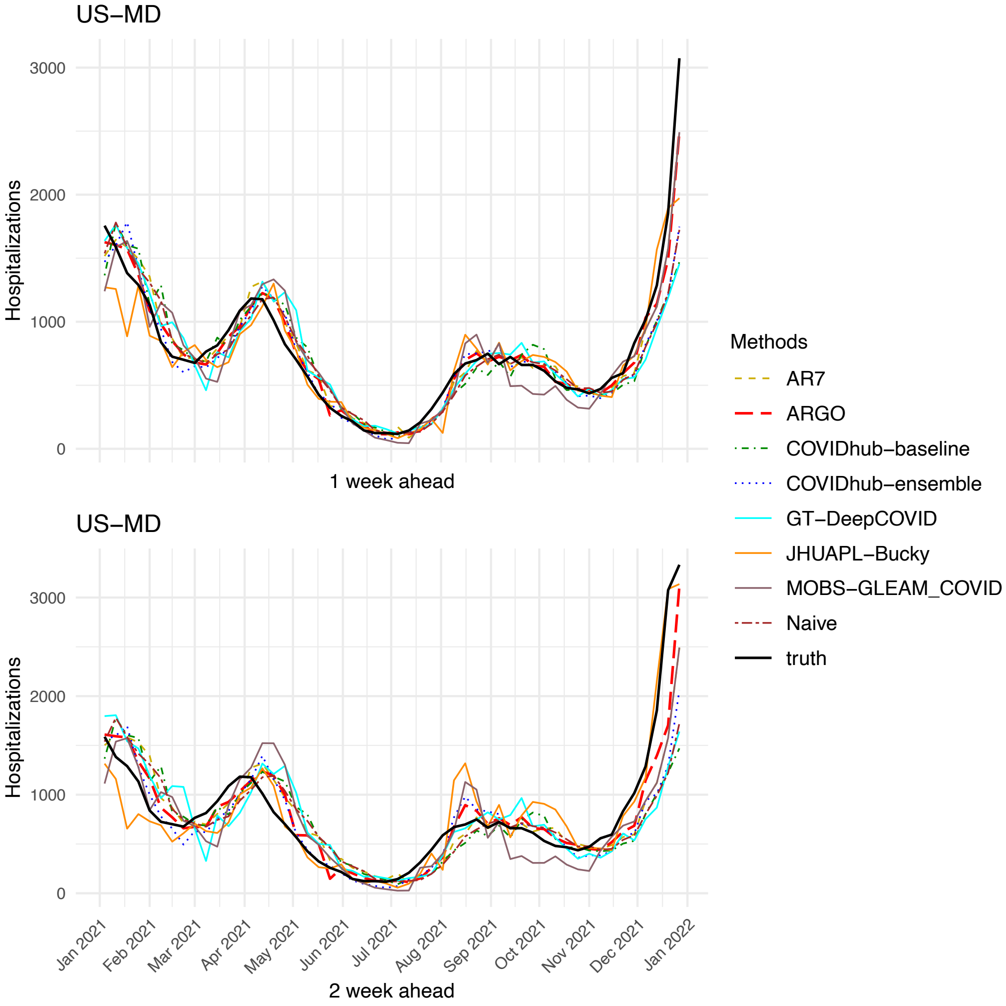

Figure S47. Plots of the COVID-19 hospitalizations 1 week (top), 2 weeks (bottom) ahead estimates of all compared models for New Maryland (MD).

Methods	RMSE		MAE		Cor	
	1 Week Ahead	2 Week Ahead	1 Week Ahead	2 Week Ahead	1 Week Ahead	2 Week Ahead
ARGO	**209.962**	**463.977**	**143.836**	**319.985**	**0.974**	0.862
AR7	223.781	477.016	156.253	346.971	0.968	0.835
COVIDhub-ensemble	386.932	603.366	252.827	396.173	0.951	**0.873**
Naive	305.156	551.819	209.654	375.365	0.939	0.788
MOBS-GLEAM_COVID	397.913	522.436	273.021	376.229	0.930	0.869
COVIDhub-baseline	296.298	545.556	199.038	360.673	0.945	0.801
GT-DeepCOVID	386.025	698.010	272.459	488.488	0.949	0.813
JHUAPL-Bucky	469.580	628.119	339.331	451.020	0.857	0.756

Table S51. Comparison of different methods for state-level COVID-19 1 to 2 weeks ahead hospitalizations predictions in Alabama (AL). The MSE, MAE, and correlation are reported and best performed method is highlighted in boldface.

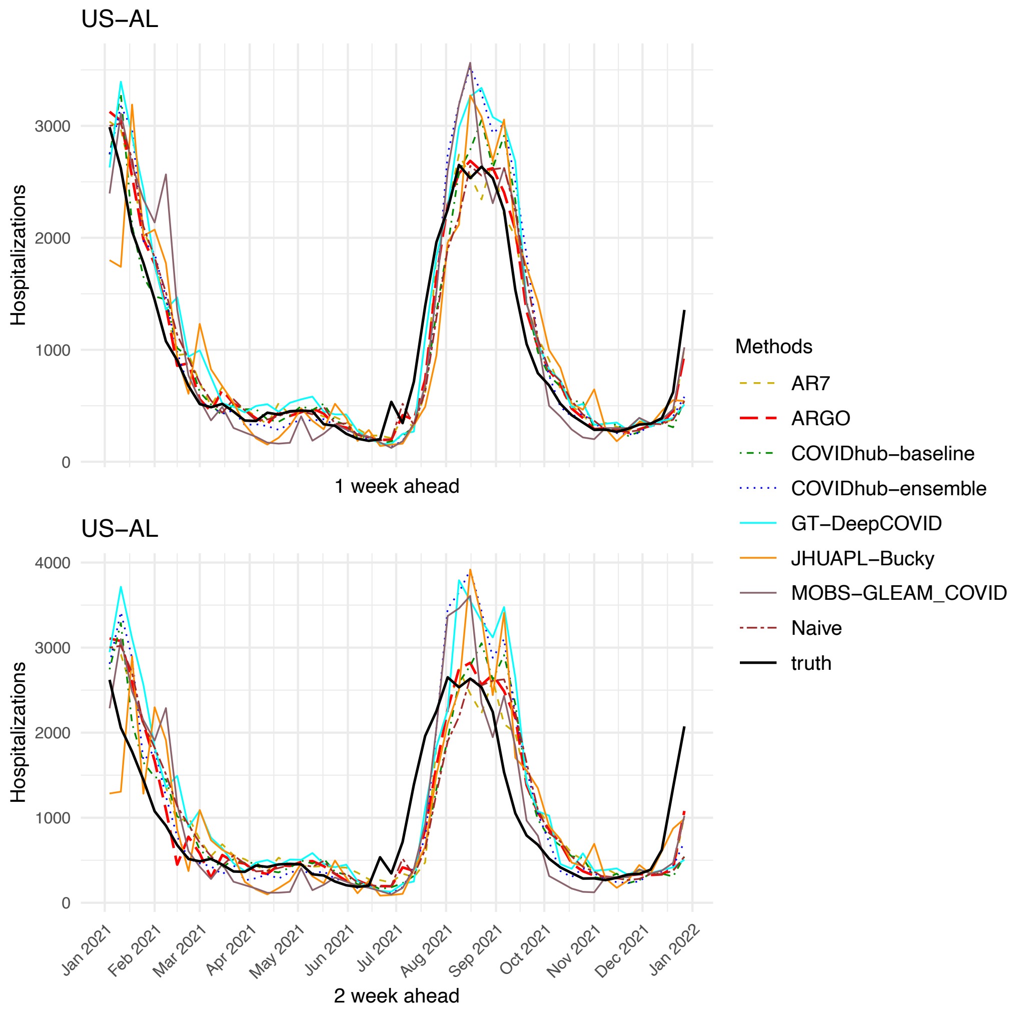

Figure S48. Plots of the COVID-19 hospitalizations 1 week (top), 2 weeks (bottom) ahead estimates of all compared models for New Alabama (AL).

Methods	RMSE		MAE		Cor	
	1 Week Ahead	2 Week Ahead	1 Week Ahead	2 Week Ahead	1 Week Ahead	2 Week Ahead
ARGO	**129.526**	**272.484**	**96.264**	**199.592**	**0.972**	**0.874**
AR7	163.866	309.442	113.818	225.714	0.954	0.835
COVIDhub-ensemble	192.947	289.086	137.173	206.500	0.936	0.871
Naive	185.534	336.480	141.596	257.365	0.932	0.796
MOBS-GLEAM_COVID	224.774	329.355	168.402	252.954	0.931	0.843
COVIDhub-baseline	225.626	365.762	159.519	269.327	0.902	0.762
GT-DeepCOVID	252.037	397.956	187.827	300.490	0.903	0.775
JHUAPL-Bucky	765.690	890.613	437.979	508.849	0.572	0.505

Table S52. Comparison of different methods for state-level COVID-19 1 to 2 weeks ahead hospitalizations predictions in Missouri (MO). The MSE, MAE, and correlation are reported and best performed method is highlighted in boldface.

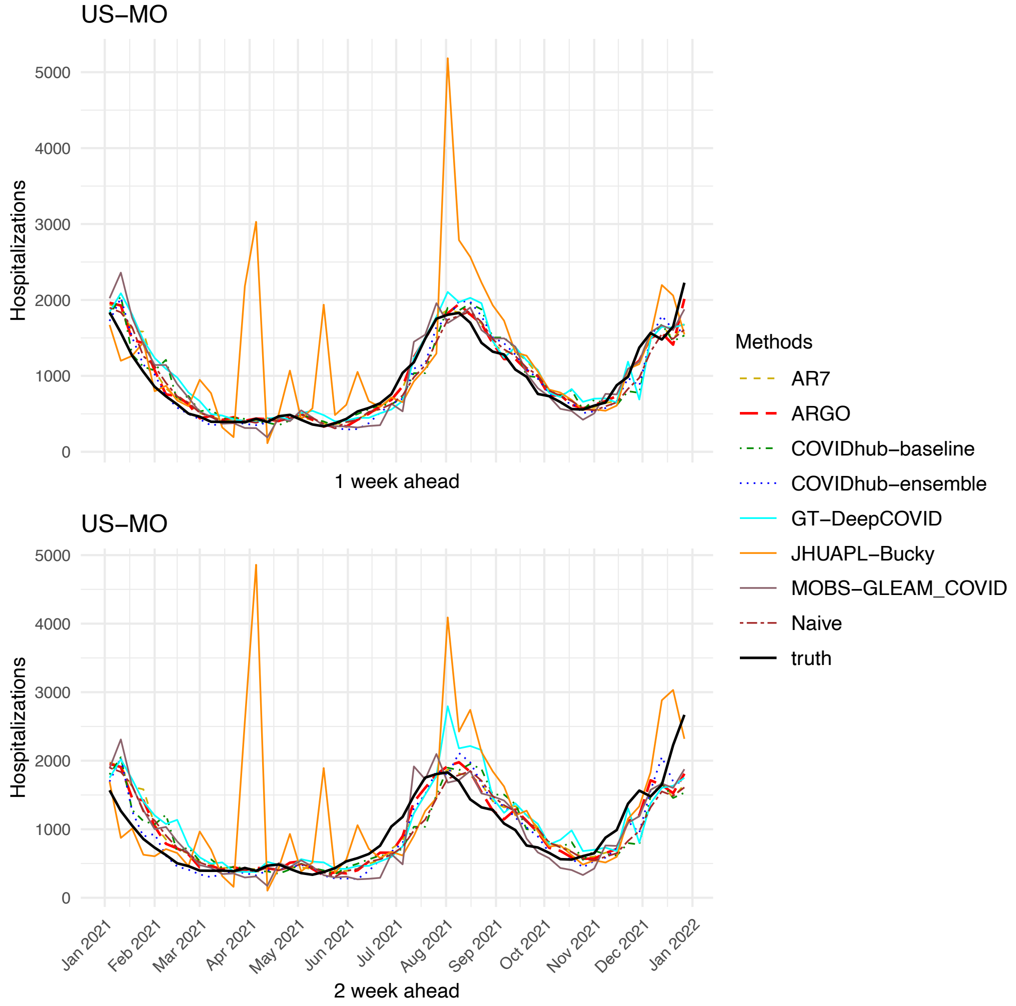

Figure S49. Plots of the COVID-19 hospitalizations 1 week (top), 2 weeks (bottom) ahead estimates of all compared models for New Missouri (MO).

Methods	RMSE		MAE		Cor	
	1 Week Ahead	2 Week Ahead	1 Week Ahead	2 Week Ahead	1 Week Ahead	2 Week Ahead
ARGO	**126.048**	**251.517**	**94.747**	190.974	**0.978**	**0.918**
AR7	133.824	273.208	106.503	213.514	0.977	0.902
COVIDhub-ensemble	160.870	267.971	120.615	**185.923**	0.967	0.916
Naive	168.777	293.922	130.192	228.673	0.958	0.885
MOBS-GLEAM_COVID	222.136	327.327	165.285	241.244	0.940	0.878
COVIDhub-baseline	154.440	270.920	129.731	220.288	0.966	0.903
GT-DeepCOVID	265.648	382.921	204.227	301.182	0.909	0.817
JHUAPL-Bucky	357.833	443.747	265.208	328.514	0.888	0.855

Table S53. Comparison of different methods for state-level COVID-19 1 to 2 weeks ahead hospitalizations predictions in Wisconsin (WI). The MSE, MAE, and correlation are reported and best performed method is highlighted in boldface.

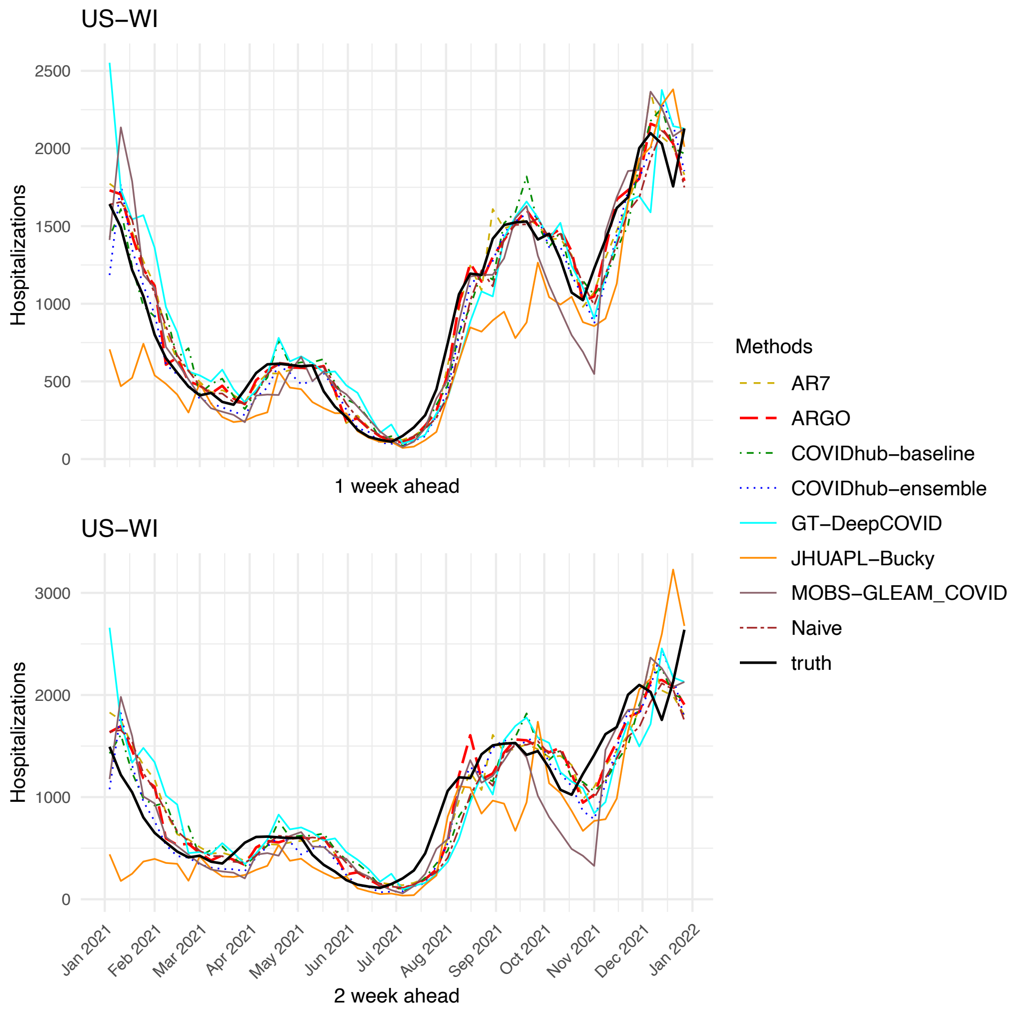

Figure S50. Plots of the COVID-19 hospitalizations 1 week (top), 2 weeks (bottom) ahead estimates of all compared models for New Wisconsin (WI).

Methods	RMSE		MAE		Cor	
	1 Week Ahead	2 Week Ahead	1 Week Ahead	2 Week Ahead	1 Week Ahead	2 Week Ahead
ARGO	**201.898**	**341.561**	**146.537**	272.739	**0.967**	0.921
AR7	248.998	414.342	173.771	311.518	0.956	0.862
COVIDhub-ensemble	216.647	364.337	156.692	**260.231**	0.963	0.910
Naive	252.979	428.509	192.692	339.538	0.940	0.837
MOBS-GLEAM_COVID	223.461	346.341	169.630	268.696	0.963	**0.924**
COVIDhub-baseline	246.917	412.754	186.269	321.423	0.942	0.848
GT-DeepCOVID	323.479	492.643	252.133	393.445	0.908	0.812
JHUAPL-Bucky	293.929	572.103	214.833	382.662	0.941	0.891

Table S54. Comparison of different methods for state-level COVID-19 1 to 2 weeks ahead hospitalizations predictions in Indiana (IN). The MSE, MAE, and correlation are reported and best performed method is highlighted in boldface.

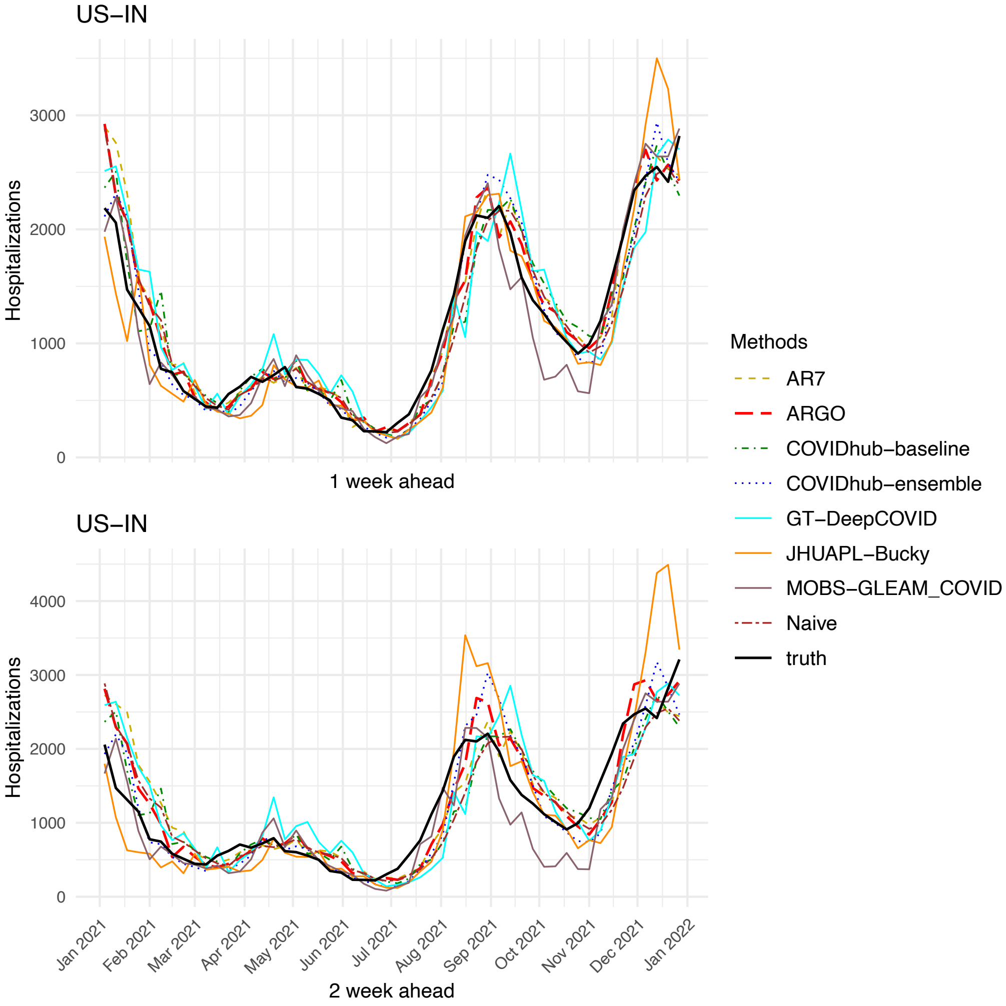

Figure S51. Plots of the COVID-19 hospitalizations 1 week (top), 2 weeks (bottom) ahead estimates of all compared models for New Indiana (IN).

Methods	RMSE		MAE		Cor	
	1 Week Ahead	2 Week Ahead	1 Week Ahead	2 Week Ahead	1 Week Ahead	2 Week Ahead
ARGO	**539.491**	**1870.584**	**371.383**	**1142.924**	**0.991**	**0.905**
AR7	799.802	2213.070	580.603	1510.918	0.979	0.842
COVIDhub-ensemble	1611.048	2737.881	948.596	1625.808	0.929	0.813
Naive	1602.244	2993.683	999.288	1888.058	0.913	0.712
MOBS-GLEAM_COVID	1695.726	3050.612	1123.527	1934.591	0.926	0.790
COVIDhub-baseline	1563.981	2946.363	945.750	1814.865	0.917	0.722
GT-DeepCOVID	1418.859	2834.502	988.366	1936.426	0.941	0.770
JHUAPL-Bucky	2678.431	3979.651	1681.721	2416.568	0.887	0.757

Table S55. Comparison of different methods for state-level COVID-19 1 to 2 weeks ahead hospitalizations predictions in Florida (FL). The MSE, MAE, and correlation are reported and best performed method is highlighted in boldface.

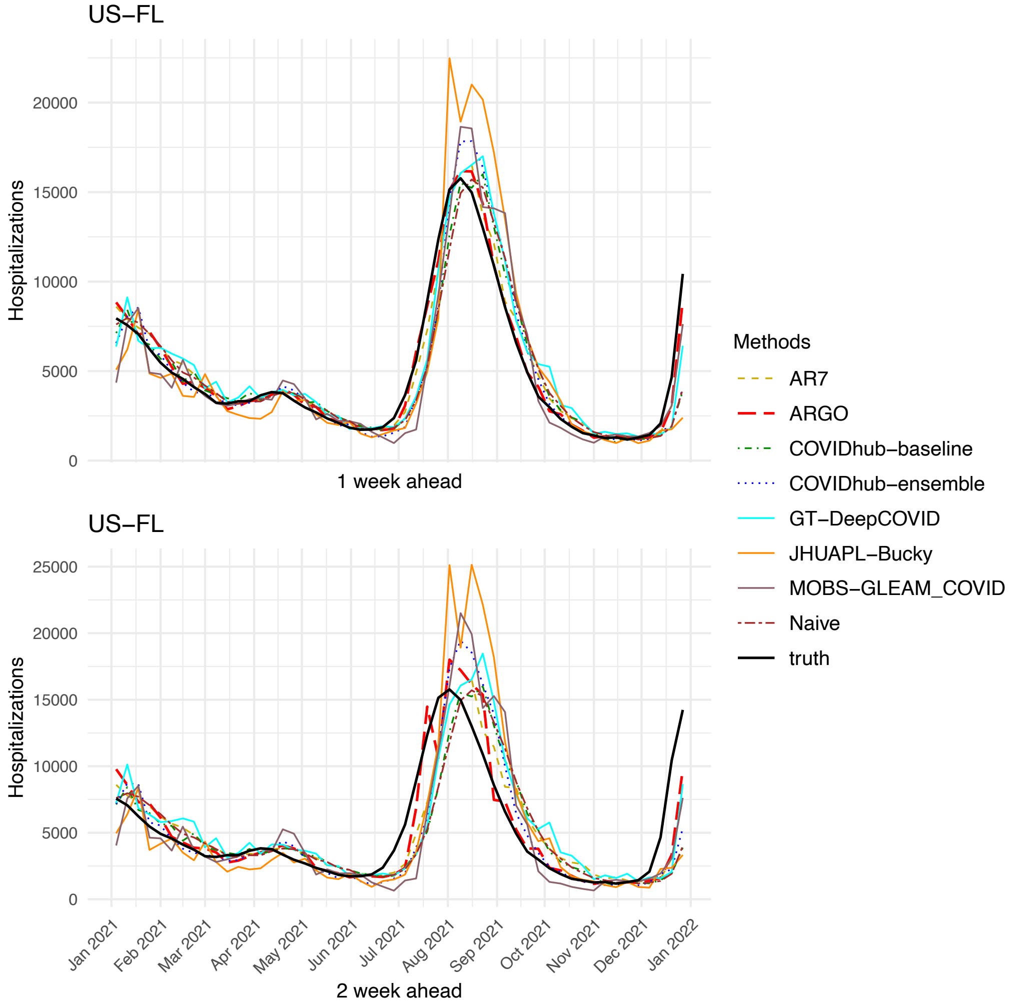

Figure S52. Plots of the COVID-19 hospitalizations 1 week (top), 2 weeks (bottom) ahead estimates of all compared models for New Florida (FL).

Methods	RMSE		MAE		Cor	
	1 Week Ahead	2 Week Ahead	1 Week Ahead	2 Week Ahead	1 Week Ahead	2 Week Ahead
ARGO	**133.820**	**330.992**	**100.462**	**209.788**	**0.971**	0.857
AR7	152.635	362.102	115.727	235.757	0.963	0.825
COVIDhub-ensemble	221.614	395.270	127.942	230.538	0.924	0.804
Naive	234.116	443.354	147.269	279.788	0.909	0.730
MOBS-GLEAM_COVID	175.217	363.410	136.032	249.937	0.959	0.851
COVIDhub-baseline	380.940	536.810	201.308	305.519	0.762	0.600
GT-DeepCOVID	250.643	466.511	159.137	299.153	0.898	0.723
JHUAPL-Bucky	317.474	484.717	205.560	338.362	0.900	**0.873**

Table S56. Comparison of different methods for state-level COVID-19 1 to 2 weeks ahead hospitalizations predictions in Virginia (VA). The MSE, MAE, and correlation are reported and best performed method is highlighted in boldface.

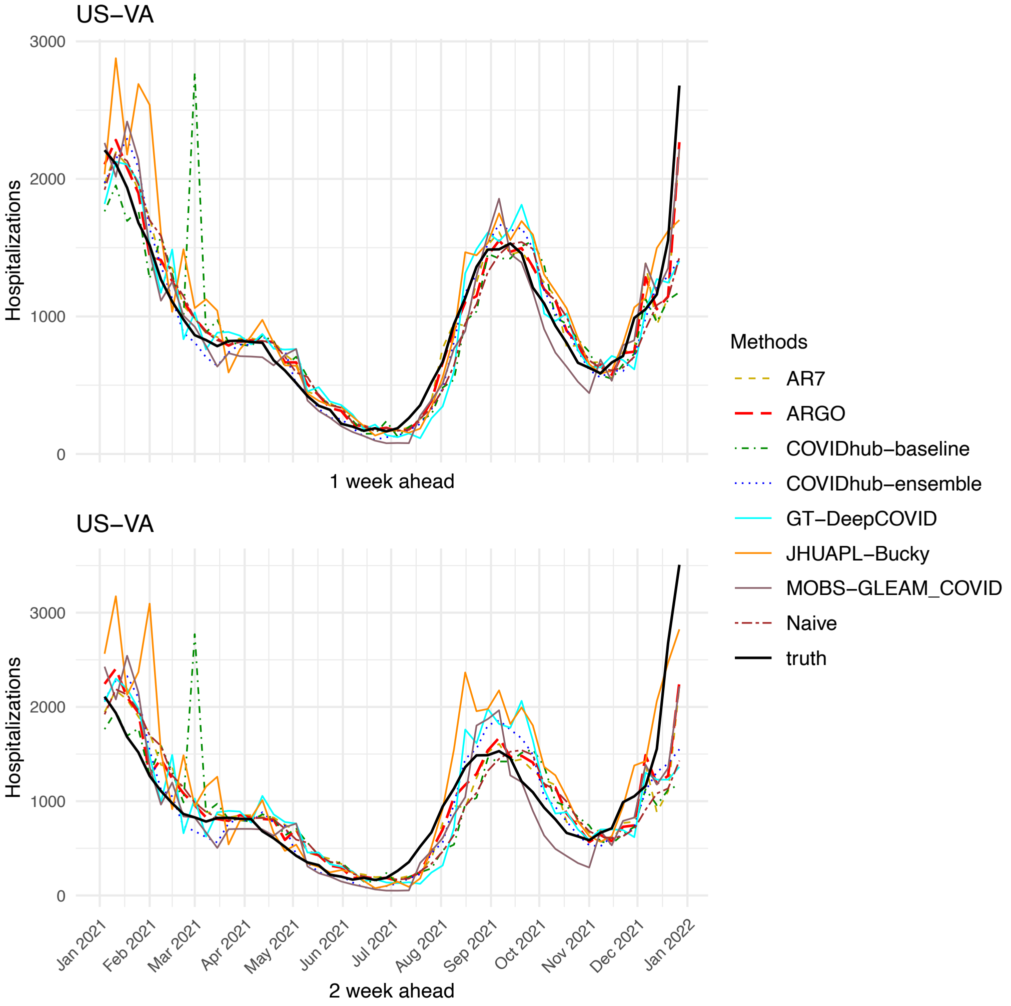

Figure S53. Plots of the COVID-19 hospitalizations 1 week (top), 2 weeks (bottom) ahead estimates of all compared models for New Virginia (VA).
